# Quantum-Chemical Study for Understanding the Low Incorporation of 2‑Methylen-1,3-Dioxepane (MDO) in Radical Ring-Opening Copolymerization with Vinyl Monomers

**DOI:** 10.1021/acs.macromol.6c00681

**Published:** 2026-07-16

**Authors:** Mikel Irigoyen, Alice Marchand, Xabier Lopez de Pariza, Timo Melchin, Aitor Barquero, Jon M. Matxain, Haritz Sardón, Jose R. Leiza, Fernando Ruipérez

**Affiliations:** † University of the Basque Country (EHU), POLYMAT and Department of Polymers and Advanced Materials: Physics, Chemistry and Technology, Faculty of Chemistry, Paseo Manuel de Lardizábal 3, 20018 Donostia−San Sebastián, Spain; ‡ 83067 Wacker Chemie AG, Johannes-Hess-Straße 24, 84489 Burghausen, Germany; § POLYMAT, Applied Chemistry Department, Faculty of Chemistry, Joxe Mari Korta Center, University of the Basque Country (EHU), 20018 Donostia−San Sebastián, Spain; ∥ Faculty of Chemistry, 39123 University of the Basque Country (EHU) and Donostia International Physics Center (DIPC), 20018 Donostia−San Sebastián, Spain; ⊥ POLYMAT and Physical Chemistry Department, Faculty of Pharmacy, 16402 University of the Basque Country (EHU), 01006 Vitoria−Gasteiz, Spain

## Abstract

Radical ring-opening polymerization (rROP) of cyclic ketene acetals (CKAs), particularly 2-methylene-1,3-dioxepane (MDO), offers a robust pathway to introduce degradable ester linkages into nondegradable vinyl backbones. However, the rational design of these materials is currently hindered by highly comonomer-dependent open-to-closed (ester vs acetal) incorporation ratios. In this study, a rigorous density functional theory (DFT) framework integrating systematic conformational sampling and implicit solvation is employed to establish a unified structure–reactivity map for MDO copolymerization across a diverse set of vinyl monomers. The computational results reveal that while MDO homopolymerization is thermodynamically driven toward quantitative ring opening due to the irreversibility of the open-chain radical, copolymerization is governed by a fine-tuned competition between the kinetic accessibility of vinyl addition and the stability of the resulting cyclic adduct. The closed-propagation barrier is primarily dictated by a SOMO–LUMO interaction, where the electronic nature and α-substitution of the comonomer modulate orbital overlap and energy. Three distinct reactivity regimes are identified: (1) high open incorporation (e.g., MDO, crotonates), where high vinyl barriers and adduct reversibility favor β-scission; (2) intermediate competition (e.g., vinyl acetate); and (3) ring-retention dominance (e.g., acrylates), where rapid vinyl addition and deep thermodynamic stabilization suppress the degradable pathway. This work provides a qualitative molecular basis for the rational selection of comonomers to maximize CKA incorporation in the open form for next-generation sustainable vinyl polymers.

## Graphical abstract

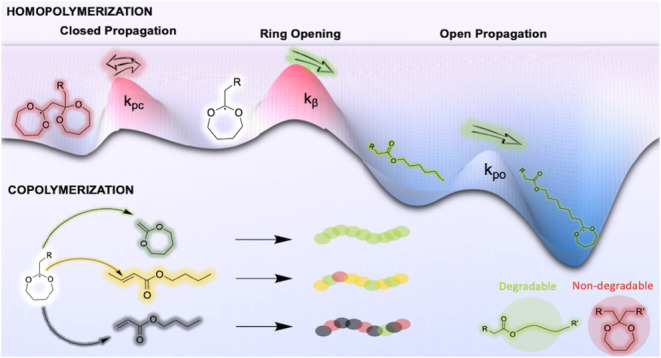

## Introduction

Plastics are indispensable in modern society, yet their persistence in the environment and their impact on human and planetary health highlight the urgent need for sustainable alternatives.^1^ Growing environmental pressures, finite fossil resources, and global commitments to carbon neutrality have intensified efforts toward developing recyclable and degradable polymers.^2,3^ Conventional free-radical polymerization (FRP) allows the production of common vinyl polymers, including polyethylene, polystyrene, and poly­(methyl methacrylate), owing to its robustness, scalability, and tolerance toward impurities.^4^ However, FRP produces polymer backbones composed mostly of C–C bonds, which resist degradation under realistic environmental or industrial conditions.^5,6^ As a result, end-of-life routes such as mechanical recycling often lead to property loss, while chemical recycling remains energetically demanding, highlighting the need for intrinsically degradable alternatives.^7,8^

Polymers containing cleavable heteroatom linkages in their backbone, such as polyesters, polythioesters, and poly­(phosphoesters), can undergo hydrolytic or enzymatic degradation.^9–14^ However, these materials are typically produced via condensation or ionic ring-opening polymerizations, which restrict monomer availability, limit tunability of mechanical properties, and lack the processing advantages of radical methods.^15^ Radical ring-opening polymerization (rROP) bridges this gap by enabling the selective insertion of cleavable heteroatom-containing bonds into otherwise nondegradable vinyl backbones through addition–fragmentation of cyclic comonomers.^16^ This hybrid mechanism preserves the operational simplicity, scalability, and compatibility of FRP while providing controlled degradability, thereby offering a promising route to recyclable or environmentally responsive vinyl materials.^17^

Among rROP monomers, cyclic ketene acetals (CKAs), such as 2-methylen-1,3-dioxepane (MDO), remain the most broadly explored. CKAs polymerize to yield polyesters and can be copolymerized with diverse vinyl monomers, including acrylates, methacrylates, crotonates, and vinyl esters, to introduce ester linkages directly into the polymer backbone.^18–23^

In the specific case of MDO, radical addition to the exocyclic double bond generates a cyclic acetal radical intermediate that can either propagate through ring-retaining vinyl addition or undergo unimolecular β-scission to form an open-chain ester radical (Figure 1). At the initiation stage, the primary radical denoted as R* may add to either MDO (M_1_) or the vinyl comonomer (M_2_), thereby producing two distinct types of propagating species. The addition to M_2_ yields a vinyl radical, whereas addition to MDO directly generates the cyclic acetal intermediate from which ring retention and ring opening compete. The competition between these pathways governs the fraction and distribution of degradable ester units incorporated into the backbone and, therefore, directly controls the material’s degradability.

**Figure 1:**
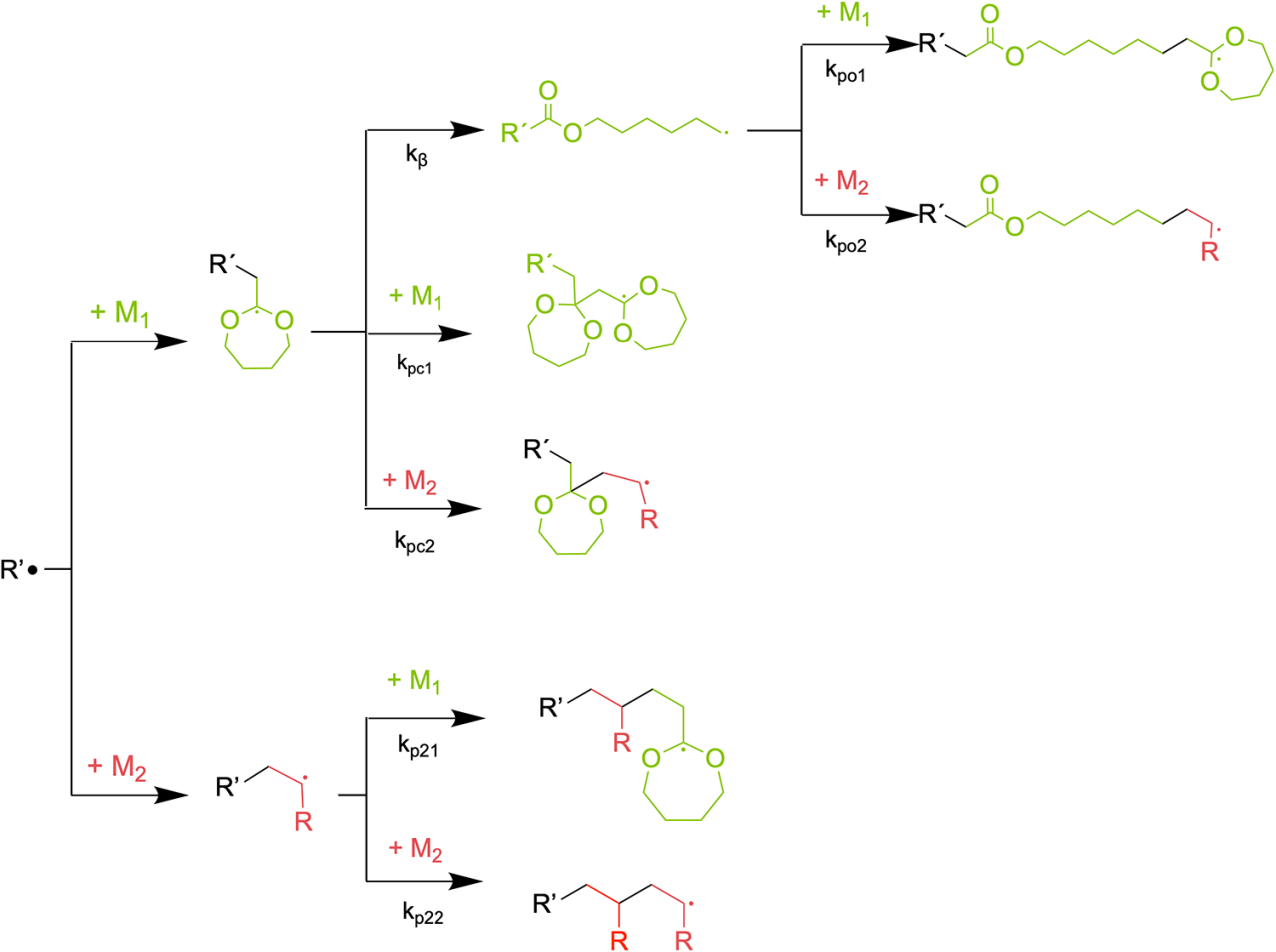
Reaction scheme for radical copolymerization of MDO (M_1_) with a generic vinyl comonomer (M_2_). Radical addition to the exocyclic double bond yields a cyclic acetal propagating radical, which can either undergo ring-retaining vinyl propagation (closed incorporation) or ring opening via β-scission to generate an ester-centered radical that propagates through the open pathway, thereby embedding degradable ester linkages in the polymer backbone.

Despite the synthetic versatility of CKAs, their apparent reactivity remains highly comonomer-dependent: many systems display low intrinsic reactivity toward electron-poor vinyl monomers, leading to strongly system-dependent open-to-closed incorporation ratios and complicating translation to predictable copolymer design.^24,25^

For efficient and uniform (bio)­degradability, two independent challenges must be addressed. MDO units must be incorporated in a sufficiently dispersed manner to avoid long nondegradable sequences, and the incorporation event itself must favor ring opening so that degradable ester linkages are formed rather than inert acetal motifs. Accordingly, considerable effort has been devoted to quantifying the open-to-closed incorporation ratio of MDO across different copolymer systems. Although additional processes such as chain transfer (to monomer, solvent, or polymer) and termination also occur during free-radical polymerization, the present work focuses specifically on the elementary propagation and ring-opening steps that most directly govern the open-to-closed incorporation balance of MDO.

Experiments have shown that MDO homopolymerization proceeds with nearly quantitative (∼100%) open incorporation,^26^ establishing the intrinsic preference of the monomer to undergo β-scission. In copolymerizations, however, the open-to-closed ratios vary significantly depending on the comonomer. High levels of open incorporation have been reported in several systems: Barquero et al. found up to ∼90% open MDO incorporation in *n*-butyl crotonate (nBCr) copolymers,^27^ while Carter et al. demonstrated substantial ester incorporation in vinyl acetate (VAc)/MDO latex copolymers produced by starved semibatch emulsion polymerization.^28^ Mothe et al. also reported successful incorporation of up to 9 mol % MDO in (meth)­acrylate/MDO latexes synthesized by batch emulsion polymerization, although the corresponding open-to-closed incorporation ratios were not disclosed.^29^ In contrast, Wenzel et al. observed much lower open incorporation for *n*-butyl acrylate (nBA)/MDO copolymer latexes synthesized under semibatch conditions,^30^ illustrating how comonomer electronic structure and steric effects critically modulate the competition between open and closed incorporation.

Despite these advances in synthesis and formulation, the underlying factors governing the reactivity of CKAs remain not fully understood, and comprehensive mechanistic frameworks and predictive structure–reactivity relationships are still lacking.^17^ The central challenge lies in understanding how the competition between ring-opening and direct vinyl propagation determines degradable-linkage incorporation, an interplay that ultimately dictates the polymer’s chemical structure and, thus, its degradability.^27–29,31–33^

Over the past two decades, density functional theory (DFT) has become integral to polymer chemistry, enabling qualitative insight into reaction mechanisms and reactivity trends,^34^ so it comes as no surprise that it has also been used to gain a deeper understanding of the aforementioned competing mechanisms. Early computational studies on CKAs elucidated how activation barriers, bond elongation, and ring strain control the competition between ring opening and vinyl propagation.

Reddy Mothe and co-workers compared radical ring-opening thermodynamics and kinetics across CKA ring sizes from five to eight members,^35^ providing a comparison of reactivity trends within this family. Their analysis also considered competing processes such as backbiting and related intramolecular reactions that can influence propagation pathways. These studies established important qualitative relationships; however, radical addition and fragmentation processes are highly sensitive to small variations in activation energies, such that achieving quantitative predictions requires a rigorous treatment of the underlying energy landscape. Further theoretical works extended these analyses to copolymerization, predicting reactivity ratios and sequence distributions consistent with experimental results, with Tardy and co-workers combining DFT and kinetic modeling to rationalize CKA ring-opening efficiencies across different systems.^25,26^

More recently, computational and experimental investigations have identified thionolactones as promising comonomers for introducing cleavable linkages into vinyl polymers, expanding the chemical scope of degradable materials.^36^ In parallel, new modeling frameworks have emerged: Nishimura and co-workers developed a kinetic model clarifying how structural asymmetry governs propagation in five-membered CKAs,^37^ and Li and co-workers integrated quantum-chemical calculations with numerical simulations to connect polymerization kinetics to degradation behavior in backbone deconstructable systems.^38^ Together, these studies demonstrate how modern computational approaches can now bridge detailed mechanistic understanding with qualitative, and in some cases even quantitative, predictions of polymerization and degradation behavior.

In this study, the radical reactivity of 2-methylen-1,3-dioxepane (MDO) toward representative vinyl comonomers is examined by modeling the elementary steps that govern incorporation, including ring-retaining vinyl propagation, unimolecular β-scission, and propagation of the opened radical. Particular attention is paid to the accurate construction of free-energy profiles for these competing pathways, which requires systematic exploration of the large conformational space associated with the molecular species participating in chain-growth polymerization. The computed energy landscapes reveal the steric and electronic factors controlling open incorporation and rationalize the experimentally observed variations in open-to-closed ratios across different copolymer systems.

## Computational Methods

The reaction mechanisms investigated in this work were explored using density functional theory (DFT). Achieving quantitatively reliable activation free energies for radical ring-opening polymerization requires particular care in the definition of the electronic-structure approach and molecular model. Radical reactions constitute a stringent test for DFT, and benchmark studies show that predicted barrier heights may vary by 4–8 kcal mol^–1^ across commonly used functionals, differences large enough to translate into rate constants that differ by several orders of magnitude.^39,40^ The situation is further complicated in open-shell systems, where spin contamination, long-range charge transfer, and latent multireference character can introduce additional uncertainty.^41,42^ Among the available approaches, hybrid meta-GGA functionals of the M06 family^43^ have been shown to provide improved performance over older hybrids such as B3LYP^44,45^ for radical addition and fragmentation processes.^46^

A faithful description of the addition–fragmentation landscape characteristic of rROP also demands accurate treatment of weak intermolecular interactions, basis-set effects, solvation, and exploration of the conformational space. Diffuse functions are essential for capturing the shallow radical–monomer encounter complexes that determine bimolecular barrier heights.^47,48^ Solvent polarization can shift polar transition states by several kcal mol^–1^, and careful selection and parametrization of continuum models are therefore required.^49,50^ Furthermore, rate predictions benefit from appropriate thermochemical treatments, particularly for bond-cleavage events such as β-scission, where improved handling of low-frequency modes can mitigate systematic errors.^51–54^ Taken together, these aspects delineate the practical requirements for obtaining physically meaningful barriers in radical polymerization chemistry.^55^

Within this framework, quantum-chemical calculations were carried out using the M06–2X functional. Conformer sampling was performed for all stationary points along the investigated reaction pathways, including reactant complexes, transition states, and products, using CREST^56^ with the GFN2-xTB Hamiltonian.^57^ For each species, the ten lowest-energy conformers were selected and reoptimized at the DFT level; the lowest-energy conformer was retained for further analysis. Geometry optimizations employed the 6–31+G­(d,p) basis set.^58^

Solvent effects were included through the CPCM implicit-solvation model,^49^ parametrized for *o*-xylene. The influence of solvent polarity was additionally assessed by recomputing the β-scission step and the vinyl-addition reactions at selected dielectric constants (ε = 0, 2.27, 10, 20, and 78). Unless otherwise specified, all reported results correspond to *o*-xylene (ε = 2.27).

Reaction free-energy profiles were constructed using a reactant-complex (RC) reference state, in which the MDO-derived radical and the comonomer form a prereactive weakly bound encounter complex. All activation free energies (Δ*G* ^‡^) are reported relative to their corresponding reactant complex, ensuring that the barriers represent intrinsic chemical transformations within a consistent reference framework.

The character of each stationary point was verified by harmonic vibrational frequency analysis with no imaginary frequencies for minima and a single imaginary frequency for transition states. These calculations provided zero-point vibrational energies, together with thermal corrections to enthalpies and Gibbs free energies.

Single-point electronic energies were subsequently refined at the M06–2*X*/6–311++G­(2df,2p) level.^59^ Final Gibbs free energies were obtained by combining these electronic energies with the corresponding thermodynamic contributions. All calculations were performed with Gaussian 16.^60^

## Results and Discussion

To systematically investigate the competing reaction pathways, reaction energy profiles (REPs) were constructed to visualize the relative energetics of all intermediates and transition states along each propagation route. Reaction energy profiles provide an intuitive representation of the underlying potential energy landscape, explicitly mapping the sequence of molecular states that connect reactants, transition states, and products. As shown schematically in Figure 2, a common reference complex can evolve through two distinct transition states, each associated with an alternative propagation mechanism and leading to products of different thermodynamic stabilities. One pathway is characterized by a lower activation barrier and is therefore kinetically favored, whereas the competing pathway involves a higher barrier but yields a more stable product, reflecting thermodynamic control. The coexistence of these pathways implies that the final polymer microstructure is not determined by a single dominant reaction coordinate but rather by a delicate balance between kinetic accessibility and thermodynamic driving forces.

**Figure 2:**
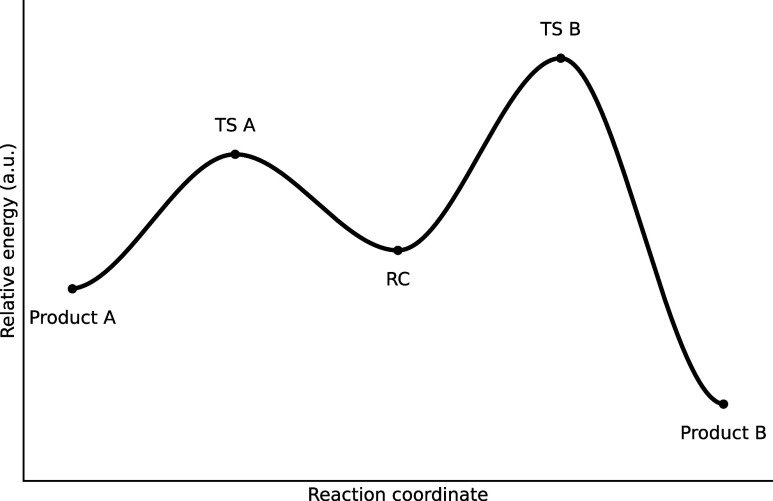
Qualitative reaction energy profile illustrating two competing polymerization propagation pathways. The reactant complex (RC) connects to two distinct transition states (TS A and TS B), leading to products of differing stability. Product B is thermodynamically favored, while Product A arises from a thermally accessible but less stable channel.

As a consequence, relatively subtle changes in the reaction conditions or molecular environment can shift the balance between competing propagation modes. This representation therefore provides a conceptual basis for understanding how molecular structure, local environment, and reaction energetics collectively govern pathway selection in polymerization and copolymerization processes involving multiple competing mechanisms.

The Results and Discussion section is organized as follows. First, the intrinsic ring-opening step of MDO (β-scission) is analyzed, and its sensitivity to the local chemical environment and solvent polarity is quantified. Next, MDO homopolymerization is examined as a mechanistic baseline, focusing on the competition between ring-retaining vinyl propagation and β-scission. Subsequently, MDO copolymerization is evaluated for experimentally benchmarked systems in terms of open-to-closed incorporation, and the analysis is later expanded to a broader set of vinyl comonomers with a focus on studying the structure–reactivity relationship. Note that all possible interactions besides vinyl propagation and ring opening of MDO have been ignored. Note that all possible interactions besides vinyl propagation and ring opening of MDO have been ignored.

The vinyl comonomers considered in the copolymerization with MDO are denoted generically as *M_n_* and comprise acrylates, methacrylates, vinyl esters, dienes, and nitriles, thereby providing a chemically diverse set of benchmarks. Their chemical structures are listed in Figure 3.

**Figure 3:**
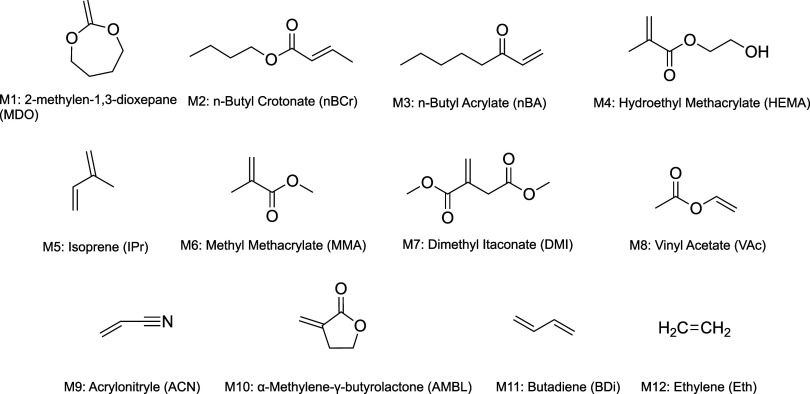
Monomers studied for the copolymerization with MDO.

### Intramolecular Ring Opening of the MDO Radical

As a first step toward rationalizing the open-to-closed incorporation behavior of MDO, the intrinsic ring-opening event (β-scission) was examined, and its sensitivity to the surrounding chemical environment was assessed (*k*_β_ in Figure 1).

This opening reaction corresponds to unimolecular C–O bond cleavage of the closed MDO radical (MDO_c_^*^), generating the open-chain radical (MDO_o_^*^) as schematically depicted in Figure 4a. The reaction free-energy profiles of the β-scission reaction were constructed in three representative local molecular environments defined by the neighboring comonomer (MDO, nBCr, and nBA), using *o*-xylene (ε ≈ 2) as solvent. The resulting activation barriers (Δ*G*_*k*_β__^‡^) show that the local chemical environment can modulate the accessibility of ring opening: while MDO and nBCr give comparable barriers (13.93 and 15.36 kcal mol^–1^, green and yellow lines in Figure 4a, respectively), nBA leads to a noticeably lower value (11.35 kcal mol^–1^, red line in Figure 4a). Thus, although β-scission is governed by the MDO C–O bond cleavage, its barrier can be shifted by specific local steric and/or electronic effects.

**Figure 4:**
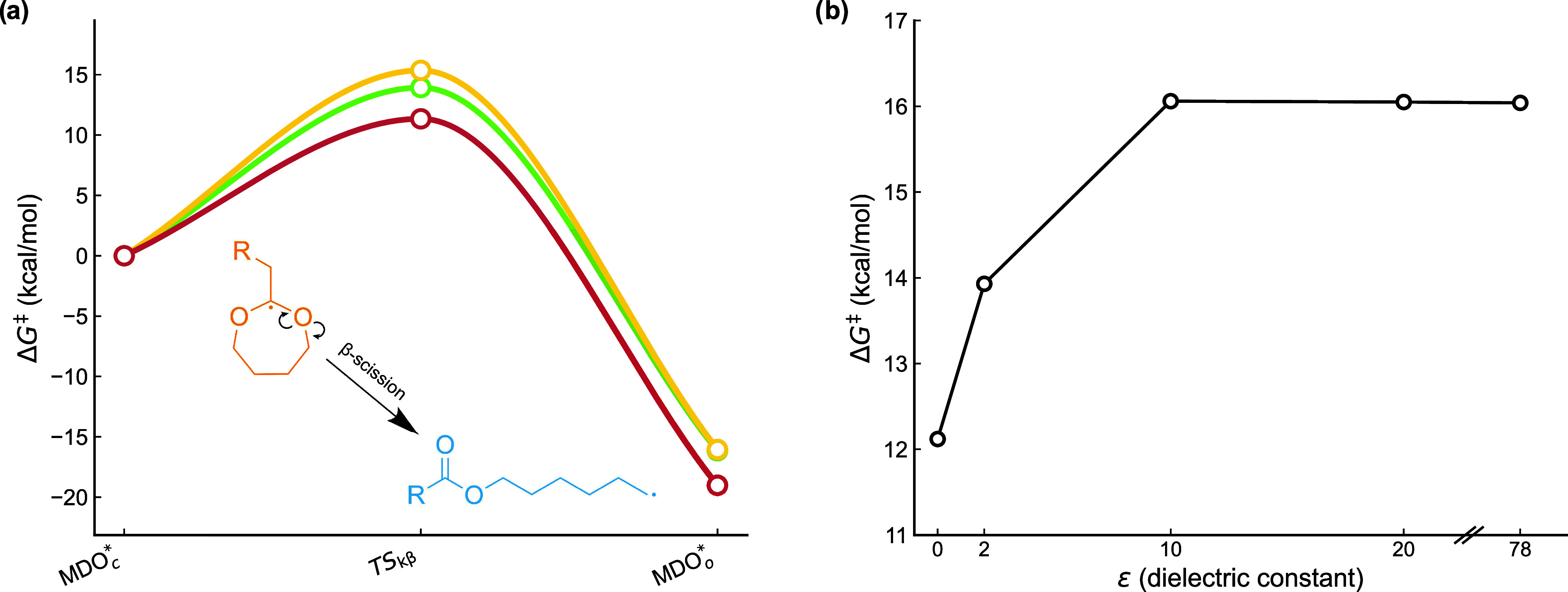
(a) β-scission activation free-energy profiles (Δ*G*_*k*_β__^‡^, kcal mol^–1^) for β-scission of MDO-derived radicals with different neighboring comonomers: MDO (green), nBCr (yellow), and nBA (red), referenced to the corresponding closed radical state (MDO_c_^*^). (b) Dependence of Δ*G*_*k*_β__^‡^ on the dielectric constant (ε) of the solvent, where ε = 0 corresponds to the gas phase, ε = 2 to *o*-xylene, and ε = 78 to water.

Given that Δ*G*_*k*_β__^‡^ falls in the ∼11–16 kcal mol^–1^ range, β-scission is expected to remain accessible under typical experimental conditions (e.g., 20–100 °C) and to become increasingly competitive as temperature is raised. This is consistent with the fact that copolymer systems exhibit a measurable, nonzero fraction of open MDO incorporation.^27–29,31–33,61^

Notably, in all cases, the open radical product (MDO_o_^*^) is substantially more stable than the corresponding closed radical, with Δ*G*_rxn_ values around −16 kcal mol^–1^ relative to the closed MDO radical (MDO_c_^*^). This indicates that ring opening is strongly exergonic across all environments considered and, once the open radical is formed, reversal to the closed state becomes highly unlikely.

In addition to varying the neighboring comonomer, solvent polarity was evaluated by recomputing the β-scission barrier as a function of the dielectric constant of the medium, ε (Table 1 and Figure 4b). To quantify solvent effects, the stabilization induced by the dielectric environment was quantified through the solvation free energy, Δ*G*_solv_, defined as

$$\Delta G_{\mathrm{solv}}=G\left(\varepsilon =0\right)-G\left(\varepsilon \right)$$

where *G*(ε = 0) and *G*(ε) denote the Gibbs free energies computed in the gas phase and in solution using the CPCM model, respectively. Negative values of Δ*G*_solv_ therefore indicate stabilization upon solvation. This quantity was computed independently for the closed MDO radical (MDO_c_^*^), in the presence of a comonomer molecule, and for their respective β-scission transition states (TS_*k*_β__), with all values referenced to their respective gas-phase energies.

**Table 1:** Relative Gibbs Free Energies (Δ*G*_solv_), in kcal mol^–1^, of the Closed MDO Radical (MDO_c_^*^) and the β-Scission Transition State (TS_*k*_β__) as a Function of the Dielectric Constant (ε), Referenced to the Gas-Phase Values (ε = 0), Together with the Corresponding β-Scission Activation Free Energies (Δ*G*_*k*_β__^‡^)

ε	Δ*G*_solv_(MDO_c_^*^)	Δ*G*_solv_(TS_*k*_β__)	Δ*G*_*k*_β__^‡^
0	0.00	0.00	12.12
2.27	–1.87	–1.24	13.93
10	–2.92	–1.04	16.06
20	–2.96	–1.25	16.06
78	–3.25	–1.39	16.04

The resulting solvation free energies, together with the corresponding β-scission activation free energies Δ*G*_*k*_β__^‡^, are summarized in Table 1. As solvent polarity increases, both the closed radical and the transition state are progressively stabilized relative to the gas phase; however, the stabilization is consistently stronger for MDO_c_^*^ than for TS_*k*_β__. This differential stabilization directly translates into an increase of the β-scission barrier, which rises from 12.1 kcal mol^–1^ in the gas phase (ε = 0) to 13.93 kcal mol^–1^ in *o*-xylene (ε = 2.27) and subsequently approaches a plateau of approximately 16 kcal mol^–1^ for ε ≥ 10.

Overall, these results show that increasing solvent polarity preferentially stabilizes the ground state of the closed radical relative to the transition state, thereby raising the activation free energy for ring opening. While neighboring comonomers introduce system-specific shifts in Δ*G*_*k*_β__^‡^, the dielectric scan reveals a clear and systematic solvent effect. The observed plateau in Δ*G*_*k*_β__^‡^ at higher dielectric constants indicates that the solvent-induced stabilization of the closed radical relative to the transition state becomes nearly constant beyond moderate polarity. The influence of the dielectric in the radical-addition reactions was also investigated, but no significant differences were observed with the varying dielectric constant (see Table S1) in the Supporting Information.

### MDO Homopolymerization: β-Scission versus Vinyl Propagation

In this section, the main focus is to assess whether the experimentally observed near-quantitative ring opening in MDO homopolymerization can be rationalized computationally when the β-scission pathway competes directly with ring-retaining vinyl propagation of the closed MDO radical.

To establish the notation used throughout the manuscript, the elementary kinetic steps considered are summarized in Figure 5, defining the competing closed-propagation (*k*_pc_) and β-scission (*k*_β_) pathways and open propagation (*k*_po_). As it was stated in the previous section, MDO_c_^*^ denotes the closed (acetal-retaining) MDO radical, whereas MDO_o_^*^ corresponds to the open radical formed after β-scission. The propagation products are labeled accordingly as MDO_*c*_MDO_c_^*^ (ring-retaining propagation product) and MDO_*o*_MDO_c_^*^ (open-propagation product after ring opening). Transition states corresponding to each elementary step are labeled as TS_*k*_pc__, TS_*k*_β__, and TS_*k*_po__, and the corresponding activation free-energy barriers are reported as Δ*G*_*k*_pc__^‡^, Δ*G*_*k*_β__^‡^, and Δ*G*_*k*_po__^‡^, respectively.

**Figure 5:**
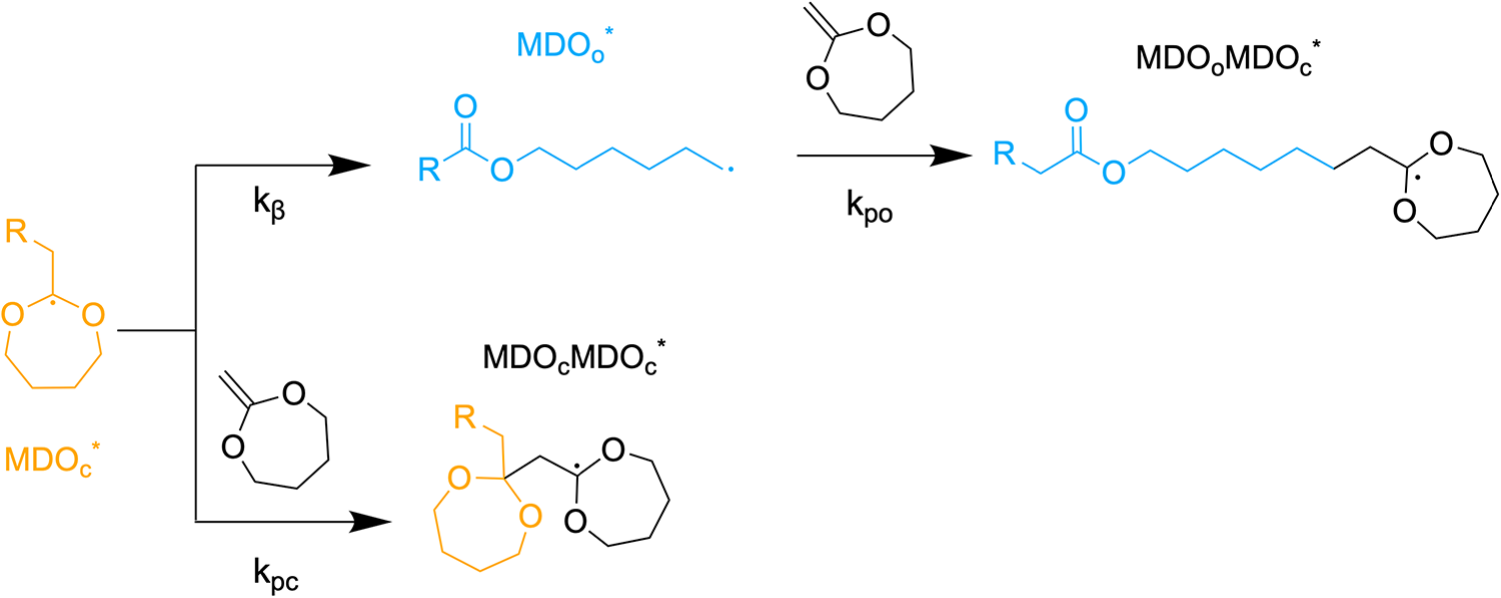
Kinetic scheme for MDO homopolymerization showing the competition between ring-retaining vinyl propagation and ring opening via β-scission. Closed acetal species are shown in orange, and open ester species in blue; all radical intermediates, propagation products, and rate constants are explicitly indicated.

The intrinsic competition between β-scission and direct vinyl propagation is the key determinant of whether MDO units are incorporated in an open (ester) or closed (acetal) form within the polymer backbone. As stated in the Introduction, experiments have shown that the open-to-closed incorporation ratio varies depending on the comonomer used as well as experimental conditions. These experimental benchmarks therefore provide essential validation points for the present computational study.

To directly connect with these observations, the computed reaction energy profile for MDO homopolymerization (Figure 6) was first constructed and established as a mechanistic baseline. This continuous profile explicitly maps all stationary points, radical and monomer encounter complexes, transition states, and propagation products, along both the β-scission (open) and ring-retaining (closed) pathways. The relative energies are reported with respect to the propagating MDO radical–monomer encounter complex (MDO_c_^*^+MDO), which is defined as the zero reference point.

**Figure 6:**
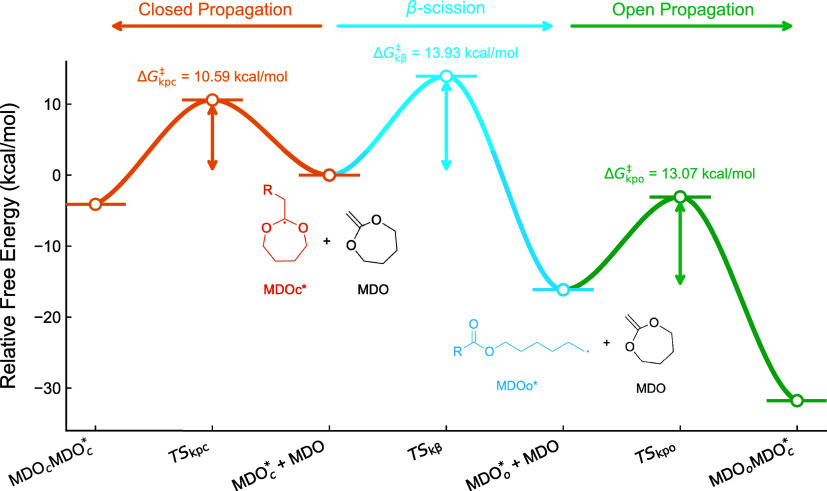
Computed reaction free-energy profile for MDO homopolymerization showing the competing pathways: closed propagation (*k*_pc_, orange), β-scission ring opening (*k*_β_, blue), and open propagation (*k*_po_, green). Energies are referenced to the MDO_c_^*^ + MDO reactant complex (0 kcal mol^–1^), and key stationary-point structures are shown.

From this species, two competing pathways can be followed: ring-retaining propagation (left) and ring opening via β-scission (right). When considering only the reaction barriers, the system exhibits a kinetic preference for the ring-retaining mechanism. The transition state for closed propagation, Δ*G*_*k*_pc__^‡^, lies at 10.59 kcal mol^–1^ above the reactant complex, whereas the β-scission transition state (Δ*G*_k_β__^‡^) is higher at 13.93 kcal mol^–1^. This difference of approximately 3.34 kcal mol^–1^ suggests that, at lower temperatures, the ring-retaining pathway should proceed somewhat faster, while the ring-opening channel remains energetically accessible.

Beyond the transition states, however, the energetic landscapes of the two channels diverge markedly. The ring-retaining pathway yields the cyclic radical MDO_*c*_MDO_c_^*^ (see Figure 5) at −4.12 kcal mol^–1^, providing only modest stabilization relative to the reactant complex. In contrast, β-scission produces the open radical MDO_o_^*^ + MDO, which is significantly more stable at −16.15 kcal mol^–1^, and subsequent open propagation, with a reaction barrier of 13.07 kcal mol^–1^, yields MDO_*o*_MDO_c_^*^ (see Figure 5) as a product at −31.77 kcal mol^–1^. These differences establish the ring-opening channel as the thermodynamically preferred route.

The reversibility of the ring-retaining channel further differentiates the two mechanisms. The forward barrier for closed propagation is Δ*G*_*k*_pc__^‡^ = 10.59 kcal mol^–1^, while the reverse barrier from the cyclic adduct back to the reactant complex is ∼14.71 kcal mol^–1^ (Figure 6, orange path), making depropagation possible. In contrast, reversal from the open radical would require overcoming a barrier exceeding 30 kcal mol^–1^ (Figure 6, blue path), rendering the ring-opening pathway effectively irreversible. Thus, once β-scission occurs, the system is driven downhill along the open-propagation channel.

As a consequence, the mechanism transitions from kinetic control to thermodynamic control. The ring-retaining channel is slightly favored kinetically, but its reversibility allows repeated equilibration and occasional β-scission events. Once the open radical is formed, the reaction becomes effectively irreversible and is thermodynamically driven toward the open-chain products as observed experimentally by Tardy and co-workers.^26^ It is noteworthy that, given the barriers obtained here and the known ∼1–2 kcal mol^–1^ uncertainty typically associated with the selected theory level for radical-addition barriers,^40,62^ the qualitative mechanistic trends remain unaffected, although such uncertainties should be considered if quantitatively derived rate constants or kinetic simulations are pursued.

### MDO Copolymerization

With the aim of understanding and rationalizing the experimentally characterized copolymer systems, the copolymerization of MDO with n-butyl crotonate (nBCr) and n-butyl acrylate (nBA) was analyzed (see Table 2 and Figure 7). These two monomers display significant differences in open-to-closed incorporation ratios, with nBCr yielding predominantly open MDO units (approximately 90%)^27^ and nBA showing substantially much lower open incorporation.^30^ These systems are therefore selected to evaluate how the comonomer structure modulates the competition between the ring-retaining and ring-opening pathways. Vinyl comonomers are denoted generically as M_*n*_ (*n* = 1, 2), and their corresponding chemical structures are listed in Figure 3.

**Table 2:** Computed Energetic and Molecular Descriptors for MDO, nBCr, and nBA^a^

monomer	Δ*G*_*k*_β__^‡^	Δ*G*_rxn,*k*_β__	Δ*G*_*k*_pc__^‡^	Δ*G*_rxn,*k*_pc__	Δ*G*_*k*_po__^‡^	Δ*G*_rxn,*k*_po__	*E*_LUMO_(eV)	ω(eV)	*V*_bur_ (%)	*r*_C–C_^TS^ (Å)	ρ_s_(*C* ^•^)
MDO	13.93	–16.15	10.59	–4.12	13.06	–15.62	–0.03	1.04	31.68	2.27	0.999
nBCr	15.36	–16.05	8.50	–12.12	11.36	–16.31	–0.32	1.26	45.66	2.33	0.925
nBA	11.35	–19.02	2.33	–21.15	6.68	–21.86	–0.59	1.43	26.45	2.46	0.902

a Activation and reaction free energies (Δ*G* ^‡^ and Δ*G*_rxn_) for the β-scission (*k*_β_), closed-propagation (*k*_pc_), and open-propagation (*k*_po_) pathways are reported in kcal mol^–1^. *E*_LUMO_, ω, and *V*_bur_ correspond to the isolated monomers, whereas *r*_C–C_^TS^ and ρ_s_(*C* ^•^) were obtained from the closed-propagation transition states and radical intermediates, respectively.

#### MDO Copolymerization with nBCr nBA

When comparing the computed energy profiles (Figure 7), both clear similarities and systematic differences across the three copolymer systems can be observed. The β-scission step shows comparable barriers (13.93, 15.36, and 11.34 kcal mol^–1^ for MDO, nBCr, and nBA, respectively; see Table 2), reflecting that the same MDO-centered C–O bond cleavage governs ring opening in all cases, with only minor perturbations from the local chemical environment. In contrast, the ring-retaining transition state (TS_*k*_pc__) varies substantially in energy because it corresponds to vinyl addition to a different comonomer.

**Figure 7:**
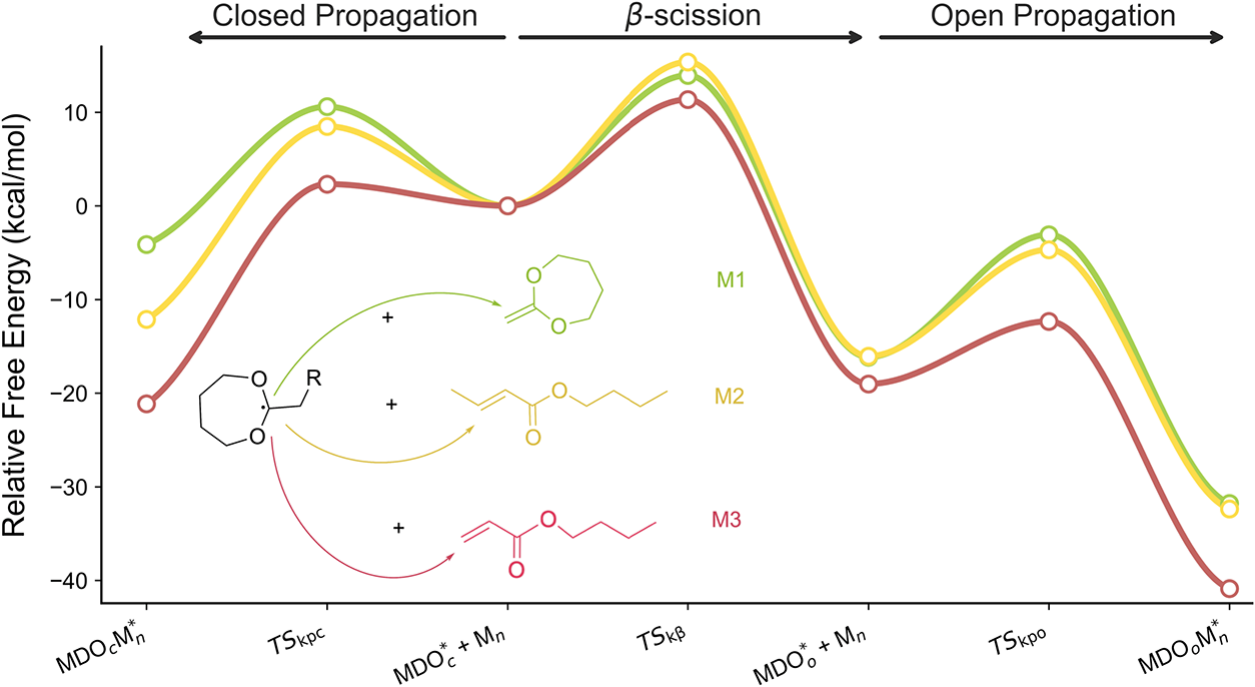
Computed reaction free-energy profiles for MDO vinyl addition and ring opening with representative monomers. Relative free energies are referenced to the corresponding reactant complex for each system. M_1_ = MDO (green), M_2_ = nBCr (orange), and M_3_ = nBA (red).

Vinyl addition proceeds through interaction between the singly occupied molecular orbital (SOMO) of the propagating MDO radical and the π* orbital of the comonomer (Figure 8). The SOMO of the closed MDO radical is localized on the carbon adjacent to the two ring oxygens, defining the nucleophilic center that initiates propagation.

**Figure 8:**
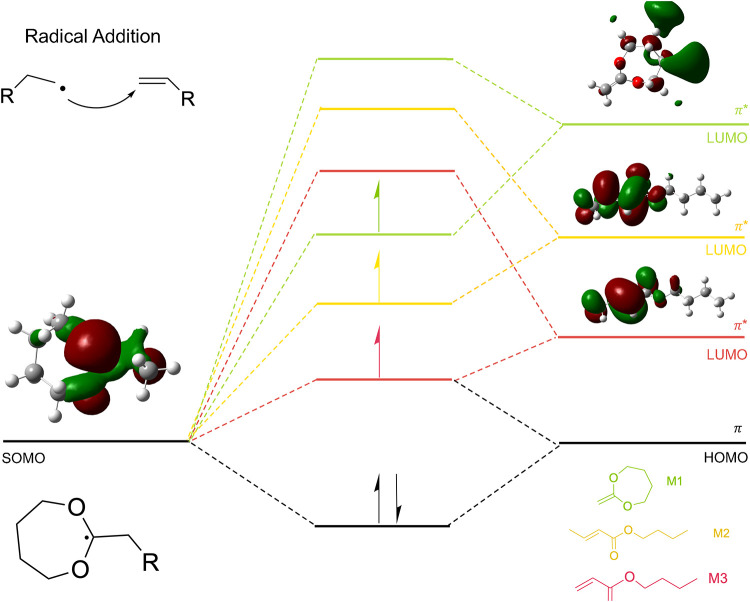
Frontier-orbital picture for the closed vinyl-addition step in MDO copolymerization. The SOMO of the propagating closed MDO radical is shown (left), together with the relevant π* LUMOs of the reacting vinyl monomers (right): MDO (green), nBCr (orange), and nBA (red).

However, the efficiency of this interaction cannot be rationalized solely in terms of frontier-orbital energies, as steric accessibility, polar effects, and transition-state stabilization also contribute significantly to the observed reactivity trends. To further analyze these factors, additional descriptors were evaluated for the benchmark systems (Table 2). The LUMO energy of the isolated monomer and the Parr electrophilicity index (ω)^63^ describe the electronic activation of the vinyl group toward radical attack, where lower LUMO energies and larger ω values favor stronger SOMO → LUMO interactions, while the buried volume descriptor (% *V*_bur_)^64^ quantifies the steric accessibility of the reacting double bond.

The SOMO → LUMO interaction differs across MDO, acrylates, and crotonates, and these differences map directly onto the observed reactivity trends. In MDO, the LUMO is relatively high in energy and shows little delocalization over the exocyclic C=C bond (Figure 8, green line). The orbital, therefore, extends only weakly into the reacting vinyl group, while the approach of two bulky MDO units introduces significant steric hindrance. Together, these effects make orbital overlap with the incoming radical both energetically and geometrically unfavorable, producing the highest vinyl-addition barrier in the series (10.59 kcal mol^–1^).

This behavior is reflected in the electronic descriptors of MDO, which exhibits the least stabilized LUMO and one of the lowest electrophilicity indices in the benchmark set (ω = 1.04 eV). The weak electrophilic activation of the vinyl group therefore limits the strength of the SOMO → LUMO interaction and contributes directly to the large propagation barrier.

Acrylates such as nBA represent the opposite limit. Strong π-conjugation between the carbonyl group and the C=C bond, together with the absence of α-substitution, substantially lowers the π* LUMO and localizes its lobes directly over the double bond (Figure 8, red line). The resulting orbital is both low-lying and ideally aligned for interaction with the radical SOMO, producing a very fast addition step and the lowest barrier observed (2.32 kcal mol^–1^).

Consistent with this picture, nBA displays both a strongly stabilized LUMO and the highest electrophilicity among the three benchmark systems (ω = 1.43 eV), indicating strong electronic activation toward radical attack. In addition, the relatively low buried volume around the reacting alkene (*V*_bur_ = 26.5%) allows the radical to approach with minimal steric distortion, further facilitating propagation.

Crotonates such as nBCr fall between these two extremes. Conjugation with the carbonyl group lowers the π* orbital relative to MDO, but the α-methyl substituent donates electron density through hyperconjugation, partially raising the LUMO compared with acrylates and shifting electron density toward the β-carbon (Figure 8, orange). This orbital is therefore intermediate in both energy and spatial distribution, which translates into the moderate vinyl-addition barrier of 8.49 kcal mol^–1^.

The influence of the α-methyl substituent is also evident in the steric descriptors. nBCr exhibits the highest buried volume in the benchmark series (*V*_bur_ = 45.7%), indicating substantial steric congestion around the reacting double bond. Thus, although crotonates benefit from electronic activation through carbonyl conjugation, the increased steric demand raises the degree of distortion required to reach the transition state and prevents propagation from becoming as facile as in acrylates.

The structural evolution of the transition states further supports this interpretation. The forming C–C bond distance increases systematically from MDO (2.27 Å) to nBCr (2.30 Å) and nBA (2.46 Å), while the activation barrier decreases in the opposite direction. This inverse relationship indicates a progressive shift toward earlier transition states across the series. In electronically activated systems such as nBA, the developing radical character becomes stabilized before substantial bond formation occurs, allowing propagation to proceed through a relatively loose transition state. In contrast, MDO requires a more advanced and geometrically constrained transition state before sufficient stabilization is achieved.

The thermodynamic stability of the closed propagation products increases from MDO to nBCr and nBA (−4.12, −12.12, and −21.15 kcal mol^–1^, relative to the corresponding reactant complexes), following the same trend as the vinyl-addition activation barriers. As the cyclic adduct becomes more stabilized, the barrier for reverse fragmentation increases, progressively suppressing depropagation. In MDO homopolymerization, the shallow minimum and steric strain of the closed adduct permit rapid equilibration, enabling almost quantitative ring opening as seen in the previous section. nBCr copolymerization remains partially reversible, particularly at elevated temperatures (e.g., 70–100 °C), maintaining high open-incorporation levels. For nBA, however, the combination of a very low addition barrier and a deeply stabilized close-propagation product heavily favors ring retention, in agreement with experimentally observed negligible open fractions.^30^

Analysis of the spin density distribution in the optimized closed adducts provides further insight into these thermodynamic trends. In nBCr, the unpaired electron remains strongly localized on the backbone radical center (ρ_s_ = 0.925), indicating limited delocalization into the crotonate framework and therefore only moderate stabilization of the closed intermediate. In contrast, nBA exhibits greater spin delocalization (ρ_s_ = 0.902), consistent with stronger conjugative stabilization by the acrylate group and the significantly deeper thermodynamic well of the corresponding adduct. MDO represents the limiting case of radical localization, with the spin density remaining almost entirely confined to the radical carbon (ρ_s_ = 0.999). This highly localized radical is only weakly stabilized in the closed adduct, which is consistent with its strong tendency to undergo reverse fragmentation and subsequent β-scission.

Together, the discussed features form a unified picture that qualitatively explains the experimentally observed sequence of open incorporation, MDO > nBCr ≫ nBA.

#### Structure–Reactivity Map and Comonomer Regimes

Having established that the computed reaction energy profiles together with the associated electronic and steric descriptors can qualitatively rationalize the experimentally observed open-to-closed incorporation ratios for MDO, nBCr, and nBA, the analysis was extended to a broader series of comonomers (Figure 3).

As discussed above, the degree of open incorporation in MDO-based copolymers arises from the interplay between the kinetic accessibility of ring-retaining propagation and the thermodynamic stability of the resulting closed radical intermediate. These effects are summarized in Figure 9, which correlates the propagation barrier (Δ*G*_*k*_pc__^‡^) with the stabilization energy of the closed adduct (Δ*G*_rxn_).

**Figure 9:**
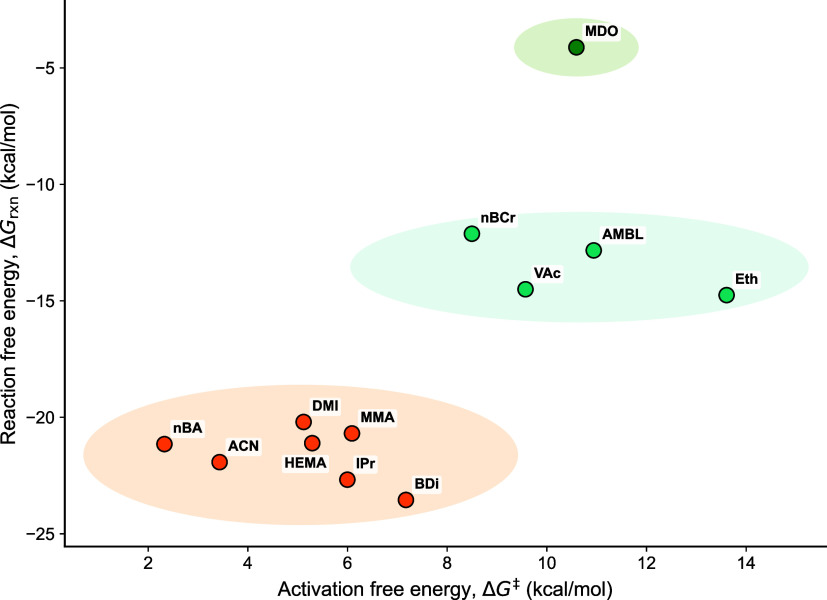
Map of activation free energies (Δ*G* ^‡^) and reaction free energies (Δ*G*_rxn_) for the investigated comonomers. Colored backgrounds highlight groups of monomers located in similar regions of the diagram.

A clear structure–reactivity pattern emerges from Figure 9, with three distinct reactivity regimes becoming apparent.

MDO (green) occupies a unique high-barrier, weakly stabilized region of the diagram. As discussed previously, its relatively high-lying LUMO, poor orbital localization over the exocyclic double bond, and only modest stabilization of the closed radical intermediate collectively favor reversible propagation and efficient ring opening.

A first intermediate regime (blue) includes butyl crotonate (BCr, M_2_), vinyl acetate (VAc, M_8_), and α-methylene-γ-butyrolactone (AMBL, M_10_), with ethylene lying near the upper edge of this region. These monomers exhibit moderate propagation barriers (approximately 8–11 kcal mol^–1^, and 13–14 kcal mol^–1^ for ethylene) together with intermediate stabilization energies of approximately −12 to −15 kcal mol^–1^.

As observed previously for the benchmark nBCr system, this intermediate regime arises from a balance between moderate electronic activation and increasing steric restriction around the reacting double bond. Crotonates benefit from ester–alkene conjugation, which lowers the π* orbital relative to MDO, but the α-methyl substituent partially offsets this activation while simultaneously increasing steric congestion. Vinyl acetate, in contrast, combines comparatively weak electrophilic activation (ω = 1.09 eV, *E*_LUMO_ = −0.00 eV) with one of the least hindered reacting double bonds in the series (*V*_bur_ = 23.3%), whereas AMBL displays intermediate values in both descriptors (ω = 1.11 eV, *E*_LUMO_ = −0.11 eV, *V*_bur_ = 27.8%). Ethylene represents the limiting case of minimal steric congestion (*V*_bur_ = 18.4%) but extremely poor electronic activation, reflected in its high-lying LUMO (+0.22 eV) and low electrophilicity (ω = 1.07 eV), which together maintain a comparatively large propagation barrier despite the absence of steric hindrance.

In this regime, the propagation and β-scission barriers remain sufficiently close that both channels are kinetically accessible, while the closed radical intermediates are only moderately stabilized. Consequently, ring opening continues to compete effectively with propagation, and significant fractions of open MDO incorporation are predicted even though ring-retaining propagation remains kinetically preferred at low temperatures. At elevated temperatures, increased accessibility of the β-scission barrier further enhances reversibility and shifts the balance toward open propagation.

A second, strongly activated regime (orange) includes *n*-butyl acrylate (BA, M_3_), acrylonitrile (ACN, M_9_), methyl methacrylate (MMA, M_6_), hydroxyethyl methacrylate (HEMA, M_4_), isoprene (IPr, M_5_), and butadiene (BDi, M_11_). These monomers combine low propagation barriers (2–8 kcal mol^–1^) with strongly stabilized closed products (−19 to −23 kcal mol^–1^). As discussed for nBA, strong electronic activation of the vinyl group substantially lowers the propagation barrier and promotes rapid formation of the closed radical intermediate. This trend is reflected in the descriptor analysis (Table S2), where ACN, nBA, HEMA, and DMI exhibit the lowest LUMO energies (−0.49 to −0.80 eV) and highest electrophilicity indices (ω = 1.33–1.54 eV) in the series, producing highly favorable SOMO → LUMO interactions and therefore very small propagation barriers.

At the same time, the resulting closed radical intermediates become strongly stabilized through delocalization of the unpaired electron away from the backbone radical center. This effect is particularly evident for the conjugated dienes IPr and BDi, which, despite only moderate electrophilicity values (ω ≈ 1.06–1.07 eV), display very low spin densities on the radical carbon (ρ_s_ = 0.66–0.72), consistent with extensive π-delocalization and some of the deepest thermodynamic wells in the series. MMA illustrates the simultaneous contribution of steric effects, where the additional α-methyl substituent increases the buried volume from 26.5% in nBA to 32.1%, raising the propagation barrier from 2.33 to 6.09 kcal mol^–1^ despite their otherwise similar electronic activation (ω = 1.43 for nBA and 1.32 eV for MMA).

In this strongly activated regime, ring-retaining propagation becomes markedly favored. The low propagation barriers enable rapid formation of the closed adduct, while the deep thermodynamic wells strongly suppress reverse fragmentation. As a result, the kinetic products become effectively trapped, and the thermodynamically favored open pathway competes only weakly. Experimentally, small but nonzero open fractions are still observed for several systems, indicating that β-scission remains accessible as a minor competing pathway.

In conclusion, these results unify the different reactivity regimes within a common mechanistic framework in which the competition between ring-retaining propagation and β-scission is governed by the coupled influence of electronic activation, steric accessibility, and radical stabilization. Electronic descriptors such as *E*_LUMO_ and ω primarily control the kinetic accessibility of vinyl addition through the strength of the SOMO → LUMO interaction, whereas steric congestion (% *V*_bur_) and spin delocalization (ρ_s_) modulate transition-state distortion and the reversibility of the closed pathway. The markedly different incorporation behaviors observed across the investigated comonomer families therefore emerge directly from their underlying molecular structure, providing a coherent structure–reactivity framework for rationally tuning open incorporation and the degradable ester content of the resulting copolymers.

## Conclusions and Future Work

A DFT-based framework was established to study the radical ring-opening copolymerization (rROP) of MDO. The protocol combines systematic conformational sampling, implicit solvation using the CPCM model parametrized for *o*-xylene, and corrected free-energy profiling to calculate, on a consistent energetic scale, the three key elementary steps controlling incorporation: unimolecular β-scission (*k*_β_), ring-retaining vinyl propagation (*k*_pc_), and propagation of the opened radical (*k*_po_).

The calculations show that β-scission is energetically accessible at room temperature and produces an open radical that is substantially stabilized relative to the closed intermediate. Solvent polarity provides a means to tune this process by preferentially stabilizing the closed radical and increasing Δ*G*_*k*_β__^‡^, whereas increasing the temperature facilitates barrier crossing and promotes ring opening.

Placing these energetics within the full propagation scheme rationalizes the behavior observed in MDO homopolymerization. Although closed propagation is kinetically favored, the resulting acetal-retaining intermediate is only moderately stabilized and remains reversible. Repeated equilibration of this species enables occasional β-scission events, which redirect the system toward the strongly exergonic open pathway. Once generated, the open radical propagates essentially irreversibly, consistent with the experimentally observed near-quantitative ring opening in MDO homopolymerization.

In copolymerization, the balance shifts from the intrinsic accessibility of β-scission to the nature of the comonomer. Variations in comonomer electronic structure and steric environment modify the propagation barrier through changes in the SOMO → LUMO interaction between the propagating radical and the vinyl monomer, thereby determining the extent to which ring-retaining propagation suppresses ring opening. More broadly, the results show that the open-to-closed incorporation balance arises from the combined influence of electronic activation of the vinyl group, steric accessibility of the transition state, and stabilization of the resulting radical intermediate. Electronic descriptors such as *E*_LUMO_ and ω primarily influence the kinetic accessibility of propagation, whereas steric congestion (% *V*_bur_) and radical delocalization (ρ_s_) affect transition-state distortion and the reversibility of the closed pathway, respectively. Importantly, the calculated incorporation trends are consistent with the experimentally observed open-to-closed ratios across the benchmark comonomer series. Together, these results provide a structure–reactivity framework linking comonomer structure and polymerization conditions to the extent of open incorporation and, consequently, to the ester content of the resulting copolymers.

Nonetheless, the present study considers only the elementary propagation and ring-opening steps directly governing the competition between open and closed incorporation. Other processes, including chain transfer, backbiting, and secondary radical rearrangements, were not explicitly examined. Although such reactions may influence molecular weight distributions and polymer microstructure under certain conditions, the agreement between the calculated energetic trends and the experimentally observed incorporation behavior suggests that the propagation/β-scission competition constitutes the primary mechanistic factor controlling open incorporation in these systems. Extension of the present framework to include transfer and secondary radical processes or kinetic studies to determine the reactivity ratios of MDO/comonomer systems (that are key to define the copolymer microstructure) therefore represents logical directions for future work.

## Supplementary Material



## Notes

Solvent dielectric effects on Gibbs free energies and activation barriers for closed- and open-propagation pathways (Table S1); computed energetic, electronic, and steric descriptors for all studied comonomers (Table S2); computed rate constants for propagation, β-scission, and reverse fragmentation reactions (Table S3); Cartesian coordinates of the optimized geometries, electronic energies, vibrational data, for monomers, radicals, reactant complexes, transition states, and intermediates (PDF)
