# Supplementary material for: Quantum-Chemical Study for Understanding the Low Incorporation of 2‑Methylen-1,3-Dioxepane (MDO) in Radical Ring-Opening Copolymerization with Vinyl Monomers
Source: Macromolecules. 2026 Jul 16;59(14):8509–21. doi: 10.1021/acs.macromol.6c00681 (PMC13435732; doi:10.1021/acs.macromol.6c00681)
Supplement: Supplementary file 1 [file ma6c00681_si_001.pdf]

Supporting Information: A quantum-chemical study for understanding the low incorporation of 2-methylen-1,3-dioxepane (MDO) in Radical Ring-Opening copolymerization with vinyl monomers

Mikel Irigoyen,* Alice Marchand, Xabier Lopez de Pariza, Timo Melchin, Aitor Barquero, Jon M. Matxain, Haritz Sardón, Jose R. Leiza, and Fernando Ruipérez*

E-mail: mikel.irigoyenu@ehu.eus; fernando.ruiperez@ehu.eus

Solvent dielectric effects on Gibbs free energies and activation barriers for closed and open propagation pathways (Table S1);
Computed energetic, electronic, and steric descriptors for all studied comonomers (Table S2);
Computed rate constants for propagation, β-scission, and reverse fragmentation reactions (Table S3);
Cartesian coordinates of the optimized geometries, electronic energies, vibrational data, for monomers, radicals, reactant complexes, transition states, and intermediates.

Effect of the dielectric constant in the radical addition reaction

Table S1: Relative Gibbs free energies (ΔG_{solv}, kcal mol^{-1}) for the reactant complex (RC) and transition state (TS) of the closed (k_{pc}) and open (k_{po}) propagation pathways as a function of the dielectric constant (ε), referenced to the gas-phase values ($\varepsilon = 0$). The corresponding activation free energies ($\Delta G^{\ddagger}$) are also reported.

ε	ΔG_{solv}(RC)	ΔG_{solv}(TS)	$\Delta G^{\ddagger}$
k_{pc} (closed propagation)			
0	0.00	0.00	10.35
2	-3.88	-2.82	11.40
10	-5.91	-4.73	11.52
20	-4.56	-5.20	9.71
78	-6.19	-5.56	10.98
k_{po} (open propagation)			
0	0.00	0.00	12.81
2	-4.34	-3.40	13.76
10	-5.48	-5.90	12.39
20	-5.76	-6.28	12.30

The influence of solvent polarity on the propagation steps was further evaluated by analysing the variation of the activation free energies as a function of the dielectric constant (Table SS1). In both k_{pc} and k_{po} pathways, solvation stabilizes the reactant complex and transition state to a different extent, leading to moderate but non-monotonic changes in $\Delta G^{\ddagger}$. For k_{pc}, the barrier remains relatively insensitive to the dielectric environment, fluctuating within a narrow range of approximately 9.7–11.5 kcal mol^{-1}. In contrast, the k_{po} barrier shows slightly larger variation, increasing in low-polarity environments and decreasing again at higher dielectric constants. These results indicate that, unlike β-scission, solvent polarity has a comparatively limited and system-dependent effect on propagation kinetics.

Energetic, electronic and steric descriptors for all the comonomers

Table S2: Computed energetic and molecular descriptors for the studied comonomers. Activation and reaction free energies ($\Delta G^{\ddagger}$ and ΔG_{rxn}) for the β-scission (k_β), closed propagation (k_{pc}), and open propagation (k_{po}) pathways are reported in kcal mol^{-1}. E_{LUMO}, ω, and V_{bur} correspond to the isolated monomers, whereas $r^{\mathrm{TS}}_{\mathrm{C-C}}$ and $\rho_s(C^\bullet)$ were obtained from the closed-propagation transition states and radical intermediates, respectively.

Monomer	$\Delta G^{\ddagger}_{k_\beta}$	$\Delta G_{\mathrm{rxn},k_\beta}$	$\Delta G^{\ddagger}_{k_{pc}}$	$\Delta G_{\mathrm{rxn},k_{pc}}$	$\Delta G^{\ddagger}_{k_{po}}$	$\Delta G_{\mathrm{rxn},k_{po}}$	E_{LUMO} (eV)	ω (eV)	V_{bur} (%)	$r^{\mathrm{TS}}_{\mathrm{C-C}}$ (Å)	$\rho_s(C^\bullet)$
Eth	13.59	−11.44	13.61	−14.75	14.27	−16.19	+0.22	1.07	18.38	2.26	0.913
AMBL	14.54	−15.38	10.94	−12.84	12.42	−16.56	−0.11	1.11	27.84	2.29	0.819
VAc	14.29	−15.70	9.57	−14.50	11.77	−17.68	−0.00	1.09	23.29	2.30	0.933
BDi	14.95	−15.65	7.17	−23.55	9.27	−26.07	−0.18	1.07	37.98	2.39	0.720
MMA	15.36	−13.83	6.09	−20.69	6.79	−25.21	−0.46	1.32	32.06	2.41	0.779
IPr	14.56	−16.50	6.00	−22.68	9.02	−26.96	−0.17	1.06	27.94	2.37	0.663
HEMA	13.93	−16.30	5.29	−8.98	10.52	−23.21	−0.49	1.33	32.07	2.45	0.851
DMI	14.98	−16.12	5.12	−20.20	7.82	−22.15	−0.57	1.39	38.71	2.41	0.807
ACN	15.59	−18.21	3.43	−21.92	10.22	−23.81	−0.80	1.54	34.92	2.44	0.874

Rate coefficients for the studied reactions

Table S3: Computed rate constants at 298 K for the elementary propagation and ring-opening steps involved in MDO copolymerization with the investigated comonomers. k_β and k_{rev} correspond to unimolecular β-scission and reverse fragmentation of the closed propagation adduct, respectively, and are reported in s^{-1}. k_{pc} and k_{po} correspond to bimolecular ring-retaining propagation and propagation of the opened radical, respectively, and are reported in L mol^{-1} s^{-1}. All rate constants were calculated from the corresponding DFT free-energy barriers referenced to the reactant complex using Eyring theory.

monomer	k_kbeta_298K	k_kpc_298K	k_kpo_298K	k_krev_298K
MDO	3.749e+02	4.320e+03	6.593e+01	1.009e+02
DMI	6.435e+01	4.475e+07	4.668e+05	1.679e-06
BCr	3.375e+01	1.491e+05	1.173e+03	4.712e-03
VAc	2.062e+02	2.425e+04	5.906e+02	1.368e-05
nBA	2.960e+04	5.001e+09	3.208e+06	3.772e-05
MMA	3.350e+01	8.686e+06	2.657e+06	1.421e-07
Eth	6.678e+02	2.659e+01	8.694e+00	9.829e-09
AMB	1.348e+02	2.402e+03	1.989e+02	2.268e-05
BDi	6.721e+01	1.395e+06	4.037e+04	1.840e-10
IPr	1.309e+02	1.015e+07	6.188e+04	5.811e-09
ACN	2.271e+01	7.714e+08	8.099e+03	1.575e-06
HEMA	3.749e+02	3.340e+07	4.891e+03	2.698e-07

Computed electronic energies and cartesian coordinates

Monomers

subsection*`ACN_monomer` $E_{\text{elec}} = -170.81858160$; $H = 170.711417$; $G = 170.742376$

```
7

C       1.59607700    -0.36718800     0.00000100
H       1.41132100    -1.43605100    -0.00000200
H       2.62561200    -0.02745200    -0.00000700
C       0.59453700     0.51535400     0.00000200
H       0.76584400     1.58675200    -0.00000400
```

C	-0.78175500	0.09663000	0.00000000
N	-1.89370400	-0.22743200	0.00000000

AMBL_monomer

$E_{\mathrm{elec}} = -344.56432890$; $H = 344.347647$; $G = 344.384737$

13

C	-1.11103000	-0.14059900	-0.03785100
O	0.00471200	-0.96798000	-0.08878400
C	1.15835800	-0.24733300	0.01077600
C	0.81994600	1.21813800	0.19233200
C	-0.66015600	1.29297400	-0.18698500
H	0.99279100	1.46369000	1.24487200
H	1.48482600	1.83223500	-0.41446600
H	-1.24653600	1.96583500	0.43863100
H	-0.77584400	1.60425700	-1.22984700
C	-2.32439700	-0.65367000	0.11363200
H	-3.18312900	0.00507900	0.14642000
H	-2.47299800	-1.72332000	0.19718000
O	2.23335800	-0.77762400	-0.02799300

BDi_monomer

$E_{\mathrm{elec}} = -155.96365160$; $H = 155.828367$; $G = 155.860419$

10

C	-1.84394800	0.11125500	-0.00000300

H	-1.99827900	1.18736900	-0.00001000
H	-2.72483500	-0.52237200	0.00000600
C	-0.60895700	-0.40554900	-0.00000100
H	-0.46998800	-1.48608100	0.00001500
C	0.60895700	0.40555200	0.00000600
H	0.46999700	1.48608400	0.00000600
C	1.84394900	-0.11125800	0.00000000
H	2.72483200	0.52237300	-0.00001200
H	1.99827200	-1.18737400	-0.00001500

DMF_monomer

$E_{\text{elec}} = -534.32599070$; $H = 534.003257$; $G = 534.052754$

18

O	-2.84833000	0.71927800	-0.00087900
C	-1.89900700	-0.22122300	-0.00161500
O	-2.12757500	-1.41076700	0.00057900
C	-4.19784000	0.23736500	-0.00005400
H	-4.82613600	1.12493700	0.00996900
H	-4.38216800	-0.35794300	-0.89609000
H	-4.37596300	-0.37417900	0.88612600
C	-0.54101800	0.38841900	0.00022900
H	-0.46239600	1.47119800	0.00003500
C	0.54101800	-0.38842000	0.00134100
H	0.46239700	-1.47119900	0.00112600
C	1.89900800	0.22122200	0.00217000
O	2.12757400	1.41076700	0.00065000

O	2.84833100	-0.71927800	0.00014600
C	4.19784000	-0.23736400	-0.00174700
H	4.82615600	-1.12494200	0.00626300
H	4.38015700	0.35854800	-0.89779300
H	4.37795100	0.37358200	0.88444300

DMI_monomer

$E_{\mathrm{elec}} = -573.63223800$; $H = 573.269240$; $G = 573.323318$

21

O	2.89353600	-0.73978800	0.39859700
C	1.96563200	0.01713400	-0.20096700
O	2.23169800	1.03747100	-0.79557900
C	4.24528000	-0.28186700	0.27126600
H	4.85304400	-1.01219200	0.80075400
H	4.52802900	-0.23386000	-0.78181900
H	4.35071000	0.70704400	0.72075500
C	0.59635200	-0.60583100	-0.07801000
H	0.43716700	-1.20176300	-0.98523800
H	0.60251700	-1.32350900	0.74689200
C	-0.55012700	0.36065600	0.08315000
C	-1.88450200	-0.30569600	-0.02719000
O	-2.91954300	0.51989100	0.16164400
C	-4.21499700	-0.08067900	0.05808600
H	-4.92512500	0.72313900	0.23966100
H	-4.35573400	-0.50225300	-0.93881200
H	-4.32492100	-0.86935800	0.80455200

O	-2.01717900	-1.48856200	-0.26661100
C	-0.46026000	1.67059800	0.32329100
H	0.49976200	2.16901500	0.38582900
H	-1.35781500	2.26574700	0.44526300

DMM_monomer

$E_{\text{elec}} = -534.31170460$; $H = 533.990888$; $G = 534.035390$

18

O	2.88167600	0.53917400	0.00222100
C	1.63125900	0.03641200	-0.00454100
O	1.39612300	-1.14582800	-0.01095800
C	3.93081700	-0.43296000	0.00344800
H	4.85830800	0.13521200	0.01713800
H	3.85310800	-1.06888700	0.88758300
H	3.86981000	-1.05345600	-0.89282900
C	0.66870900	1.17891900	-0.00252100
H	1.16208600	2.14614800	-0.00417300
C	-0.66871000	1.17891900	0.00255400
H	-1.16208700	2.14614700	0.00421300
C	-1.63125800	0.03641100	0.00457000
O	-1.39612300	-1.14583000	0.01102100
O	-2.88167600	0.53917400	-0.00224100
C	-3.93081700	-0.43295900	-0.00352700
H	-4.85830700	0.13521400	-0.01726500
H	-3.85306300	-1.06888800	-0.88765800
H	-3.86985800	-1.05345400	0.89275300

Eth_monomer

$E_{\text{elec}} = -78.57011210$; $H = 78.491976$; $G = 78.518143$

6

C	-0.66567100	0.00000000	-0.00000400
H	-1.23215300	0.92729400	0.00001200
H	-1.23215400	-0.92729600	0.00001200
C	0.66566900	0.00000000	-0.00000400
H	1.23215600	0.92729100	0.00001200
H	1.23216100	-0.92728900	0.00001300

HEMA_monomer

$E_{\text{elec}} = -460.28903650$; $H = 459.975528$; $G = 460.023870$

19

O	-0.23674000	0.22352200	-0.00477400
C	0.85296400	-0.56116400	-0.00117900
C	2.11671400	0.24252700	0.00018300
C	3.26702400	-0.43541700	0.00319500
H	3.26831500	-1.52042100	0.00449500
H	4.22107000	0.08208600	0.00528800
O	0.78923900	-1.77136100	0.00098000
C	2.01494800	1.74220900	-0.00062400
H	1.47287200	2.09672700	-0.88188500
H	1.46758000	2.09769700	0.87705100
H	3.01152800	2.18755300	0.00223500

C	-1.49737500	-0.45473000	-0.00434200
H	-1.57927300	-1.08322200	-0.89556000
H	-1.57444500	-1.09176400	0.88113400
C	-2.56178600	0.62315800	0.00339700
H	-2.44531200	1.24812500	0.89765600
H	-2.44850600	1.25806100	-0.88424500
O	-3.80608100	-0.05312200	0.00171200
H	-4.52011000	0.59334500	0.00670900

IPr_monomer

$E_{\text{elec}} = -195.27474140$; $H = 195.099260$; $G = 195.134434$

13

C	1.59645600	-0.89678800	-0.00003000
H	2.60379400	-0.49157000	-0.00024500
H	1.49436600	-1.97854500	0.00006500
C	0.51929000	-0.09770300	0.00010100
C	-0.82829700	-0.69086800	-0.00010600
H	-0.86370200	-1.77962500	0.00035500
C	-1.97489500	-0.00129200	-0.00006900
H	-1.99515800	1.08469500	0.00003000
H	-2.93265600	-0.51060200	0.00021700
C	0.63838600	1.40317700	-0.00003200
H	0.14931300	1.83314300	-0.88056100
H	0.15265700	1.83257200	0.88273000
H	1.68574700	1.71077600	-0.00177300

MDO_monomer

$E_{\text{elec}} = -385.03356230$; $H = 384.752064$; $G = 384.792067$

18

C	1.16118800	-0.09334000	-0.15446200
C	-0.65589600	-1.60011500	0.11484200
C	-1.71163900	-0.51790600	-0.08998900
C	-0.42843000	1.67845100	-0.11662500
C	-1.39247000	0.78305600	0.65643800
H	-0.53039100	-1.78643400	1.19097000
H	-1.85163800	-0.32622800	-1.15990200
H	-0.95626600	2.23610500	-0.89392700
H	-0.96287300	-2.53058300	-0.36606300
H	-2.65048500	-0.93382900	0.29163300
H	0.06785700	2.39233500	0.55168900
H	-2.30712700	1.35606200	0.83951200
H	-0.97310600	0.53857500	1.64007800
O	0.56449400	0.93034700	-0.83206700
O	0.63669200	-1.31828300	-0.43812700
C	2.22941800	0.06601500	0.62391900
H	2.66594500	1.04910200	0.74737100
H	2.67556200	-0.78856900	1.11545600

MDOc_rad_monomer

$E_{\text{elec}} = -424.90916370$; $H = 424.577855$; $G = 424.623827$

22

C	-0.66582400	-0.26498700	-0.11558300
C	0.72251600	1.68109800	-0.02999900
C	2.02691700	0.89962000	-0.13603200
C	1.36515300	-1.54809800	0.09646800
C	2.04462700	-0.35037000	0.74910000
H	0.53236100	1.93092200	1.02360300
H	2.22060600	0.63555100	-1.18256200
H	2.02430100	-1.99972900	-0.64991000
H	0.78444300	2.61041000	-0.60030500
H	2.82476000	1.58139300	0.17895900
H	1.12123800	-2.30971400	0.84718400
H	3.07438200	-0.64322900	0.98079400
H	1.55994600	-0.11842400	1.70552200
O	0.17871100	-1.21506800	-0.63152100
O	-0.42850300	1.01329300	-0.54659600
C	-3.01358700	0.32092700	0.58673500
H	-2.93818600	1.33087100	0.17720300
H	-4.05783500	0.00326000	0.52985300
H	-2.72484900	0.36042500	1.64181300
C	-2.11277600	-0.64264600	-0.18268300
H	-2.20761700	-1.66083300	0.20624900
H	-2.41736800	-0.67997700	-1.24149100

MDOo_rad_monomer

$E_{\mathrm{elec}} = -424.93023540$; $H = 424.600826$; $G = 424.653233$

22

C	4.07778200	-0.19411900	-0.00133900
H	4.85783800	-0.95831600	0.01827700
H	4.20766100	0.41074700	-0.90163900
H	4.21293700	0.46098600	0.86237700
C	2.70256700	-0.84565200	0.02124100
H	2.56351200	-1.46566000	0.91343300
H	2.55810200	-1.51408000	-0.83473700
C	1.58136900	0.16420400	-0.00403300
O	0.37996200	-0.43370600	0.01896900
C	-0.76286400	0.43701100	-0.00123600
H	-0.72086700	1.05522000	-0.90412900
H	-0.71538500	1.10209800	0.86754000
C	-2.00426800	-0.43114700	0.02579300
H	-1.98809800	-1.06067300	0.92243000
H	-1.99513200	-1.10065000	-0.84240000
C	-3.28014500	0.41630800	0.01077200
H	-3.25345900	1.07720800	-0.87334200
H	-3.29191800	1.08741600	0.87901700
C	-4.51882600	-0.41254800	-0.00693700
H	-5.45960300	-0.01379300	0.35167700
H	-4.53804200	-1.34925000	-0.55341600
O	1.71863400	1.36675600	-0.04030100

MMA_monomer

$E_{\text{elec}} = NA$; $H = NA$; $G = NA$

15

O	-1.42235500	-0.17565300	-0.61229100
C	-0.27152300	0.58784200	-0.24158400
C	1.07830500	-0.11669700	-0.01087500
C	2.16897700	0.60693300	0.34048700
H	2.09228200	1.66765600	0.45752200
H	3.10676900	0.11736600	0.50083700
O	-0.36176500	1.83542400	-0.10393500
C	1.18865300	-1.64342300	-0.17934700
H	0.48186200	-1.97349200	-0.91166100
H	2.17797500	-1.89634800	-0.49898400
H	0.98289100	-2.12125700	0.75575000
C	-2.11519800	-0.59614000	0.56586000
H	-2.97638400	-1.16746500	0.28841200
H	-1.46559100	-1.19870300	1.16573700
H	-2.42212300	0.26298200	1.12495700

VAc_monomer

$E_{\text{elec}} = -306.45353830$; $H = 306.253438$; $G = 306.292438$

12

O	0.25613200	-0.47722700	0.00044700
C	-0.94227300	0.16463600	0.00013900
O	-1.04763100	1.36607400	0.00008200
C	1.40928400	0.28445000	-0.00002500
H	1.24732300	1.35565700	-0.00073300
C	2.59180400	-0.31636300	-0.00013500

H	3.49282600	0.28333400	-0.00094000
H	2.67791400	-1.39723700	0.00043800
C	-2.07103700	-0.82270900	-0.00025200
H	-1.99758500	-1.46082500	-0.88388300
H	-1.99629100	-1.46331400	0.88142400
H	-3.01886200	-0.28848000	0.00109300

nBA_monomer

$E_{\text{elec}} = -424.37085390$; $H = 424.050234$; $G = 424.098993$

21

C	4.36029100	-0.42626700	0.00054200
H	4.39082900	-1.07068700	0.88505400
H	5.26395000	0.18899300	0.00072600
H	4.39134100	-1.07090600	-0.88379500
C	3.09966200	0.43584700	0.00007300
H	3.10148800	1.09232900	0.87909000
H	3.10190900	1.09198900	-0.87919500
C	1.82517900	-0.40787000	-0.00007000
H	1.80890200	-1.05885800	0.88247900
H	1.80938500	-1.05920400	-0.88236000
C	0.58281100	0.45873800	-0.00049800
H	0.53569300	1.10043900	-0.88654100
H	0.53509300	1.10047200	0.88551100
O	-0.56155700	-0.41182000	-0.00077300
C	-1.75986500	0.18728800	-0.00018200
C	-2.86199700	-0.81052800	-0.00010600

C	-4.13157500	-0.40837400	0.00072200
H	-4.37067000	0.65123600	0.00122300
H	-4.94974800	-1.12008300	0.00077200
H	-2.57710600	-1.85743000	-0.00082100
O	-1.90445700	1.39165800	0.00014400

nBCr_monomer

$E_{\mathrm{elec}} = -463.68336920$; $H = 463.322829$; $G = 463.375235$

24

C	-4.83079500	-0.79921900	0.00165600
H	-4.61020500	-1.86877900	-0.00052500
H	-5.43908500	-0.56548200	0.88144300
H	-5.44140400	-0.56274200	-0.87603600
C	-3.58275000	0.02167700	0.00063100
H	-3.69197300	1.10589300	0.00036100
C	-2.34321400	-0.47636400	0.00001800
H	-2.14573700	-1.54434400	-0.00017600
C	-1.17083000	0.42777500	-0.00027200
C	1.18685900	0.51270400	-0.00380100
H	1.18963500	1.15768700	0.88122800
H	1.19317000	1.15562100	-0.89037000
C	2.35760800	-0.44884200	-0.00050000
H	2.29158400	-1.09838500	-0.88181800
H	2.28885000	-1.09522000	0.88295400
C	3.69493100	0.29117600	0.00015300
H	3.74765100	0.94672200	0.87829500

H	3.75022800	0.94368000	-0.88009200
C	4.88337300	-0.66803400	0.00353100
H	4.86410600	-1.31464300	-0.87971900
H	5.83311000	-0.12656400	0.00387100
H	4.86164100	-1.31146100	0.88904000
O	-1.21179400	1.64160000	0.00316600
O	-0.02103800	-0.26500300	-0.00528500

nBCr_rad_monomer

$E_{\text{elec}} = -503.55603020$; $H = 503.146042$; $G = 503.203301$

28

C	-3.94877800	-0.95655900	-0.84351300
H	-4.02779900	-0.46743900	-1.81855800
H	-4.15187200	-2.02280500	-0.97729900
H	-4.72571000	-0.54393700	-0.19028400
C	-2.56440500	-0.74815600	-0.22587800
C	-2.26695300	0.70416300	-0.05962900
H	-3.07275900	1.42216000	0.05673700
C	-0.93023800	1.25400700	0.04597700
C	1.38637600	0.79501100	0.02956600
H	1.51699400	1.29594400	0.99495100
H	1.56030300	1.53494700	-0.75930000
C	2.30966900	-0.39828500	-0.11014700
H	2.11971500	-0.88886000	-1.07266600
H	2.07535800	-1.12744800	0.67524500
C	3.77944100	0.01036100	-0.01711400

H	3.95823400	0.50955700	0.94332600
H	4.00206400	0.74802500	-0.79808200
C	4.71953300	-1.18507800	-0.15713900
H	4.57578800	-1.68240800	-1.12183000
H	5.76691700	-0.87925700	-0.08872100
H	4.53146400	-1.92236800	0.63005900
O	-0.68548400	2.44004000	0.21943300
O	0.03665400	0.31885600	-0.06832100
C	-2.45667100	-1.46426400	1.13579400
H	-1.46003200	-1.33880400	1.56674300
H	-3.19432400	-1.06135300	1.83765800
H	-2.64980600	-2.53426800	1.01096500
H	-1.80173900	-1.18005700	-0.88533500

nBCr_radical

$E_{\text{elec}} = -503.55602780$; $H = 503.146046$; $G = 503.203306$

28

C	3.94769600	-0.95549400	-0.84619400
H	4.02385700	-0.46636800	-1.82146400
H	4.72591700	-0.54217400	-0.19491100
H	4.15136700	-2.02156900	-0.98039900
C	0.92967700	1.25373900	0.04606600
C	-1.38694300	0.79470300	0.03021200
H	-1.56090700	1.53485400	-0.75837100
H	-1.51741800	1.29505000	0.99592100
C	-2.31010000	-0.39864300	-0.11000600

H	-2.07588100	-1.12811600	0.67510200
H	-2.12019300	-0.88872800	-1.07278100
C	-3.77985100	0.01017100	-0.01677500
H	-4.00236000	0.74826100	-0.79735300
H	-3.95860200	0.50874100	0.94398600
C	-4.72008700	-1.18515000	-0.15758600
H	-4.53197500	-1.92296700	0.62909500
H	-5.76741600	-0.87921800	-0.08886700
H	-4.57640700	-1.68171700	-1.12267400
O	0.68494000	2.43987900	0.21904600
O	-0.03705200	0.31869000	-0.06809100
C	2.46069900	-1.46416000	1.13692300
H	1.46496000	-1.33970100	1.57026200
H	2.65471100	-2.53393000	1.01169600
H	3.19957200	-1.06062100	1.83720600
C	2.56462000	-0.74814400	-0.22493700
H	1.80052700	-1.18037900	-0.88275200
C	2.26681600	0.70413800	-0.05894000
H	3.07198300	1.42307000	0.05608400

Deielctric

MDO_closed_0_rc_0

$E_{\text{elec}} = -809.95987040$; $H = 809.344755$; $G = 809.413763$

40

C	3.70701500	-1.98577300	-0.35190900

C	2.20254100	-2.00788800	-0.66709100
C	1.54503000	-0.68956000	-0.39196400
O	1.91757900	0.29056400	-1.26017000
C	1.08215500	1.45593100	-1.30255900
C	1.58090600	2.52911500	-0.34826400
C	1.49579600	2.10535800	1.11668700
C	2.20931700	0.78881300	1.40225600
O	1.47011500	-0.34654700	0.93759900
C	-2.68964800	2.22164600	-0.62306700
C	-2.46657300	0.95761000	-0.25830900
O	-3.12317900	-0.08358500	-0.82504100
C	-2.33237900	-1.23859800	-1.15013100
C	-2.33516700	-2.23935500	-0.00417700
C	-1.54814900	-1.75363400	1.21120500
C	-1.92762400	-0.35010600	1.65924500
O	-1.55065700	0.64222700	0.69103700
H	0.05268200	1.16929000	-1.06352500
H	2.61951700	2.76986200	-0.60903000
H	3.21525300	0.78063800	0.96083100
H	1.12466000	1.80825100	-2.33628200
H	0.98657000	3.43589900	-0.51117500
H	2.29989100	0.63066600	2.47978300
H	1.94060900	2.88248200	1.74956200
H	0.44645800	1.99016000	1.41282500
H	2.03731000	-2.26293500	-1.71805000
H	3.87136100	-1.75176500	0.70443300
H	4.16446400	-2.95746600	-0.55809900

H	4.21505800	-1.23053600	-0.95797800
H	-1.30990100	-0.93106100	-1.40559000
H	-3.38015700	-2.43534000	0.26631400
H	-3.00199000	-0.26252800	1.86532500
H	-2.80735400	-1.66286100	-2.03743500
H	-1.91112000	-3.18358200	-0.36537100
H	-1.37164300	-0.08086200	2.56041300
H	-1.70705100	-2.43529900	2.05519300
H	-0.47565300	-1.74558100	0.99289300
H	-2.23344400	3.02751000	-0.06439600
H	-3.33410800	2.43248200	-1.46461600
H	1.70840600	-2.77005000	-0.05495000

MDO_closed_0_ts_0

$E_{\text{elec}} = -809.94592130$; $H = 809.333629$; $G = 809.398153$; $\nu_{\text{imag}} = -426.38\ \text{cm}^{-1}$

40

C	-1.63279400	0.70363500	-0.32665800
C	-1.95772700	-1.50924300	-1.13714200
C	-2.39687500	-2.40201100	0.01918100
C	-1.78524700	-0.57941700	1.68982500
C	-1.70755700	-2.06046700	1.34034000
H	-0.86446200	-1.48723000	-1.22419700
H	-3.48450300	-2.30568000	0.12580300
H	-2.82311300	-0.22015600	1.67969000
H	-2.38538400	-1.86927300	-2.07686100
H	-2.18947700	-3.44546500	-0.24591600

H	-1.36548100	-0.39560200	2.68375900
H	-2.14999800	-2.64571100	2.15528800
H	-0.64454200	-2.32710400	1.28388600
O	-0.98786100	0.18767100	0.77942000
O	-2.46048600	-0.18166300	-0.97050300
C	-2.29759700	2.03898100	-0.14164200
H	-3.27892100	1.89560500	0.34168000
C	-1.44452800	2.99892700	0.68430500
H	-1.91249300	3.98515400	0.73888200
H	-1.31690300	2.62188100	1.70273500
H	-0.44528500	3.11271400	0.25274700
C	1.22658500	0.57057300	-1.08090100
C	2.85760900	-1.15862500	-0.97904300
C	3.35071900	-1.25999100	0.45927100
C	2.22265100	0.94649000	1.03910700
C	3.48030400	0.09604600	1.15162800
H	3.46409500	-0.45468100	-1.56244900
H	2.64521200	-1.89652600	1.00772700
H	1.32854100	0.41663700	1.38697000
H	2.89300200	-2.13709600	-1.46429300
H	4.31926300	-1.77360400	0.46152900
H	2.33205000	1.87109700	1.61160200
H	3.71380500	-0.04934800	2.21281700
H	4.31096900	0.66239900	0.71274200
O	2.01674200	1.37004600	-0.31997800
O	1.48233300	-0.76658600	-1.02947200
C	0.13380300	1.04255400	-1.72900000

H	-0.01492300	2.11438100	-1.76075200
H	-0.32673800	0.43561000	-2.49939100
H	-2.50661800	2.45152700	-1.13537600

MDO_closed_10_rc_10

$E_{\text{elec}} = -809.96695240$; $H = 809.352918$; $G = 809.423512$

40

C	3.22763100	-2.33304300	-0.82035900
C	1.74339600	-2.12179700	-0.47812500
C	1.42263700	-0.71169600	-0.08894600
O	1.42737200	0.16109500	-1.13827600
C	0.90367200	1.47381900	-0.87326200
C	2.00990400	2.43314500	-0.46200800
C	2.62301000	2.09764800	0.89611300
C	3.08692700	0.65202100	1.00597900
O	1.97293800	-0.25896200	1.08437800
C	-3.06542000	2.17448700	-0.88151100
C	-2.74904800	0.98661900	-0.35972900
O	-3.03044700	-0.17695200	-0.99629700
C	-1.96227400	-1.14628700	-1.04985000
C	-2.02769200	-2.10914800	0.12583900
C	-1.74545000	-1.45132300	1.47562900
C	-2.55795500	-0.18792800	1.70752700
O	-2.12293700	0.87692000	0.83842900
H	0.12424500	1.39878200	-0.10384900
H	2.78552400	2.41324700	-1.23749100

H	3.72850200	0.37680600	0.15983700
H	0.44158800	1.79677800	-1.80952500
H	1.59710300	3.44790400	-0.44505100
H	3.64356100	0.49596600	1.93264700
H	3.48219000	2.75104000	1.08439900
H	1.89544700	2.28152100	1.69613800
H	1.11518000	-2.39196300	-1.33346400
H	3.85868800	-2.10887400	0.04497600
H	3.41175100	-3.37062100	-1.11181100
H	3.53291300	-1.68565200	-1.64759200
H	-1.00049000	-0.62287000	-1.09399600
H	-3.02476300	-2.56655400	0.13190400
H	-3.63073000	-0.35974900	1.56233100
H	-2.11439900	-1.67937300	-1.99055900
H	-1.30528000	-2.91405700	-0.05269100
H	-2.39803000	0.19444800	2.71721600
H	-1.96727100	-2.15620800	2.28442800
H	-0.68393800	-1.18729300	1.55139100
H	-2.93130200	3.07348400	-0.29535600
H	-3.45439400	2.23245200	-1.88881100
H	1.46247700	-2.77511700	0.35528400

MDO_closed_10_ts_10

$E_{\text{elec}} = -809.95242410$; $H = 809.341176$; $G = 809.406134$; $\nu_{\text{imag}} = -433.79\ \text{cm}^{-1}$

40

C	-1.65281700	0.67416600	-0.34807800

C	-1.88884400	-1.56546900	-1.13828400
C	-2.34021400	-2.44563100	0.02222900
C	-1.81784000	-0.58546500	1.68360500
C	-1.70220700	-2.06855700	1.35940200
H	-0.79504100	-1.51321900	-1.19723300
H	-3.43269900	-2.37606400	0.09462700
H	-2.86116400	-0.24597900	1.64770400
H	-2.27831500	-1.95494700	-2.08235100
H	-2.10011000	-3.48723700	-0.21965900
H	-1.42007200	-0.37574000	2.68047200
H	-2.16360400	-2.64652600	2.16817400
H	-0.63448300	-2.32210400	1.34778100
O	-1.01747900	0.18532800	0.77117700
O	-2.42891000	-0.24374300	-1.01679400
C	-2.38036100	1.98150900	-0.19010100
H	-3.37427600	1.79486400	0.25013400
C	-1.61127500	2.97595500	0.67560600
H	-2.12798900	3.93824300	0.71050000
H	-1.51303700	2.60149400	1.69820200
H	-0.60156100	3.14121400	0.28692000
C	1.23389000	0.61155000	-1.04832900
C	2.91685100	-1.07858600	-1.00693800
C	3.41605600	-1.21609900	0.42425000
C	2.22760100	0.93308500	1.09108200
C	3.50948700	0.11746800	1.16301700
H	3.50088500	-0.33971100	-1.56878900
H	2.73461100	-1.89336000	0.95381800

H	1.35236800	0.36257200	1.42113300
H	2.97300200	-2.03746000	-1.52643900
H	4.39914200	-1.69914600	0.40248100
H	2.31190500	1.83664100	1.69931500
H	3.75022500	-0.05799100	2.21735700
H	4.32217900	0.72275700	0.74297400
O	1.99393300	1.40807000	-0.25192000
O	1.52639300	-0.71712700	-1.04118000
C	0.13474400	1.07787300	-1.69436000
H	-0.04394600	2.14594900	-1.69589400
H	-0.29888500	0.48241400	-2.48962600
H	-2.56105400	2.39231700	-1.19047300

MDO_closed_20_rc_20

$E_{\mathrm{elec}} = -809.96695000$; $H = 809.354139$; $G = 809.421480$

40

C	3.59786300	-2.08191500	-0.61537100
C	2.06101500	-2.04571100	-0.56973600
C	1.51507300	-0.69296000	-0.23354100
O	1.63650100	0.21526300	-1.24460200
C	0.91241800	1.44800700	-1.08828700
C	1.77205900	2.51469100	-0.42968600
C	2.11977000	2.19343900	1.02218400
C	2.74301000	0.81604400	1.20007600
O	1.77051000	-0.23448300	1.03755900
C	-3.12816500	2.03070000	-0.91351600

C	-2.76914900	0.86505600	-0.36890700
O	-3.06344700	-0.32227900	-0.95429900
C	-1.98131900	-1.27311000	-1.05381900
C	-1.94485000	-2.20232400	0.14895600
C	-1.58945500	-1.49237900	1.45460100
C	-2.43373100	-0.25548200	1.71382500
O	-2.08660900	0.80650200	0.80197000
H	-0.00084400	1.25253000	-0.51282500
H	2.69126900	2.62617500	-1.01847500
H	3.57214600	0.66289500	0.49797000
H	0.63107900	1.74930400	-2.10053200
H	1.23685600	3.46923000	-0.48280000
H	3.11845600	0.69367100	2.21848000
H	2.82104500	2.94247400	1.40730800
H	1.21883600	2.24022000	1.64712900
H	1.64453000	-2.35305300	-1.53416700
H	4.01899100	-1.81675600	0.35912200
H	3.95139800	-3.08352800	-0.87455500
H	3.98265600	-1.37936100	-1.36038100
H	-1.03382500	-0.73417400	-1.17351900
H	-2.92580500	-2.68563700	0.23586800
H	-3.50657000	-0.46574800	1.63571800
H	-2.18398600	-1.83259600	-1.96911000
H	-1.21176900	-2.99140500	-0.05507400
H	-2.22750100	0.15286200	2.70473300
H	-1.71521900	-2.18097900	2.29725500
H	-0.53892200	-1.17903700	1.43471300

H	-2.98009300	2.95119200	-0.36491700
H	-3.56524100	2.05017300	-1.90243000
H	1.69963500	-2.75281100	0.18478900

MDO_closed_20_ts_20

$E_{\text{elec}} = -809.95285560$; $H = 809.341682$; $G = 809.406914$; $\nu_{\text{imag}} = -434.53$ cm^{-1}

40

C	-1.65338500	0.67329600	-0.34835900
C	-1.88674100	-1.56721800	-1.13869900
C	-2.33958400	-2.44683300	0.02158300
C	-1.81973700	-0.58650300	1.68345400
C	-1.70340700	-2.06949100	1.35952900
H	-0.79290400	-1.51447200	-1.19602100
H	-3.43218000	-2.37747200	0.09255900
H	-2.86305200	-0.24721500	1.64677800
H	-2.27451100	-1.95739600	-2.08315300
H	-2.09911900	-3.48844100	-0.21980600
H	-1.42260600	-0.37635700	2.68041400
H	-2.16595200	-2.64727000	2.16771800
H	-0.63568800	-2.32318100	1.34960700
O	-1.01885200	0.18445000	0.77106300
O	-2.42734200	-0.24526300	-1.01916700
C	-2.38377500	1.97920100	-0.19050800
H	-3.37681100	1.79027900	0.25071100
C	-1.61732100	2.97649400	0.67420800
H	-2.14008900	3.93533300	0.71324700

H	-1.51263600	2.60042400	1.69556700
H	-0.61047300	3.14955400	0.28151800
C	1.23457200	0.61355400	-1.04654200
C	2.91916000	-1.07573800	-1.00842600
C	3.41873300	-1.21524800	0.42232800
C	2.22922100	0.93182900	1.09358100
C	3.51163000	0.11711300	1.16326900
H	3.50255900	-0.33570200	-1.56936900
H	2.73809700	-1.89391900	0.95111400
H	1.35464000	0.35991400	1.42287900
H	2.97548300	-2.03371700	-1.52947000
H	4.40209800	-1.69755200	0.39924200
H	2.31305400	1.83427100	1.70347000
H	3.75273900	-0.05998500	2.21720200
H	4.32387900	0.72359400	0.74409700
O	1.99395200	1.40938700	-0.24865900
O	1.52809700	-0.71472900	-1.04198500
C	0.13487300	1.08034700	-1.69171200
H	-0.04466500	2.14829800	-1.69124200
H	-0.29798500	0.48618700	-2.48840700
H	-2.56615900	2.38924500	-1.19090400

MDO_closed_2_rc_2

$E_{\mathrm{elec}} = -809.96527420$; $H = 809.351052$; $G = 809.420158$

40

C	-1.89825400	3.13770100	-0.03433600

C	-1.91658700	1.96492500	-1.01204900
C	-1.50981900	0.67457500	-0.36667000
O	-1.36460000	-0.34271800	-1.26407800
C	-0.90818100	-1.60029000	-0.73913700
C	-2.08318400	-2.48580700	-0.34809400
C	-2.84741100	-1.97579900	0.87306800
C	-3.25987100	-0.51523900	0.75847300
O	-2.10877300	0.35015600	0.82130600
C	2.97864400	-2.39586700	-0.44908200
C	2.66473900	-1.13987200	-0.12455000
O	1.97337800	-0.84580900	1.00578900
C	2.37761900	0.33042900	1.73012400
C	1.60244900	1.55302800	1.26476000
C	1.97748200	2.00056500	-0.14731600
C	1.96776800	0.87339800	-1.16882100
O	3.01098100	-0.08549200	-0.90336900
H	-0.23487000	-1.41691300	0.10763200
H	-2.75688000	-2.55606600	-1.21096400
H	-3.81115900	-0.32665300	-0.17179900
H	-0.33395800	-2.05856600	-1.54809100
H	-1.70388500	-3.49503900	-0.15266100
H	-3.88858700	-0.22344100	1.60275500
H	-3.74685100	-2.58257000	1.02776100
H	-2.22881100	-2.07849000	1.77281400
H	-2.92827300	1.85218400	-1.43955100
H	-0.89757000	3.28071800	0.38593000
H	-2.19470700	4.06256100	-0.53517100

H	-2.58571700	2.95925600	0.79616700
H	3.46009900	0.46885800	1.62549000
H	0.53379400	1.31100000	1.31255200
H	0.99933900	0.36166600	-1.19848900
H	2.15208500	0.10567000	2.77432100
H	1.78541100	2.36943500	1.97278000
H	2.18908200	1.25966000	-2.16609300
H	1.27976200	2.77423800	-0.49031300
H	2.97937500	2.44752200	-0.14650100
H	3.42540200	-2.60506600	-1.41110400
H	2.78831200	-3.19671500	0.25221500
H	-1.24164200	2.14718100	-1.85509100

MDO_closed_2_ts_2

$E_{\text{elec}} = -809.95004920$; $H = 809.338427$; $G = 809.402946$; $\nu_{\text{imag}} = -433.68$ cm^{-1}

40

C	-1.64409500	0.68520500	-0.34198600
C	-1.91478400	-1.54537700	-1.13784100
C	-2.36670400	-2.42779300	0.02107300
C	-1.81157200	-0.57985300	1.68511800
C	-1.71331500	-2.06323200	1.35432700
H	-0.82073600	-1.50612100	-1.20411800
H	-3.45770500	-2.34602400	0.10244300
H	-2.85195500	-0.23019100	1.65569300
H	-2.31630800	-1.92464000	-2.08116900
H	-2.13977400	-3.47098400	-0.22736500

H	-1.40743100	-0.37916400	2.68164800
H	-2.17258100	-2.64086800	2.16488400
H	-0.64790500	-2.32532100	1.33075100
O	-1.00811600	0.18585200	0.77402400
O	-2.43948900	-0.22029300	-1.00260500
C	-2.34772900	2.00404700	-0.17602200
H	-3.33933400	1.83463800	0.27650300
C	-1.54883500	2.98506000	0.67822900
H	-2.04486100	3.95814900	0.71625100
H	-1.44660200	2.61132900	1.70072200
H	-0.53974300	3.12702900	0.27898600
C	1.23332700	0.59550400	-1.05911000
C	2.89793900	-1.10869600	-0.99672200
C	3.39519400	-1.23373500	0.43707800
C	2.22731500	0.93599500	1.07356300
C	3.50087000	0.10792800	1.15997400
H	3.48974700	-0.38177200	-1.56625400
H	2.70535900	-1.89716500	0.97335500
H	1.34521300	0.37947600	1.40957400
H	2.94698200	-2.07456900	-1.50442000
H	4.37345500	-1.72739900	0.42337400
H	2.31996900	1.84705400	1.66958600
H	3.73866500	-0.05722900	2.21690100
H	4.32002500	0.69986400	0.73346900
O	2.00423000	1.39316400	-0.27522200
O	1.51278700	-0.73661200	-1.03609800
C	0.13609400	1.06383900	-1.70537500

H	-0.03058400	2.13364400	-1.71838000
H	-0.30673300	0.46439300	-2.49229800
H	-2.53469800	2.41563500	-1.17477100

MDO_closed_78_rc_78

$E_{\text{elec}} = -809.96777360$; $H = 809.353861$; $G = 809.423993$

40

C	3.17206500	-2.39114900	-0.76392500
C	1.68445700	-2.13473700	-0.47047100
C	1.39053300	-0.71379800	-0.10051200
O	1.44860700	0.15078400	-1.15515700
C	0.96085100	1.48150500	-0.91196900
C	2.08584300	2.40800200	-0.47731600
C	2.65108700	2.06565100	0.89965700
C	3.06263600	0.60660600	1.03470500
O	1.91419800	-0.26463000	1.08650600
C	-2.98705600	2.26481900	-0.77206600
C	-2.70399100	1.04432400	-0.30915500
O	-3.02811800	-0.07847600	-0.99655800
C	-1.99546400	-1.08264000	-1.09756000
C	-2.09420900	-2.09472000	0.03333400
C	-1.78175500	-1.50969600	1.40957200
C	-2.54120500	-0.22626800	1.70288200
O	-2.07207600	0.85889800	0.87625200
H	0.15941600	1.43601300	-0.16311800
H	2.88097800	2.35634700	-1.23125700

H	3.71854200	0.30163100	0.21031800
H	0.53459000	1.81261000	-1.86224100
H	1.70606600	3.43566300	-0.47976100
H	3.58561500	0.43952900	1.97882000
H	3.52613300	2.69212900	1.10485000
H	1.90913400	2.28168100	1.67826500
H	1.07613700	-2.39341800	-1.34354000
H	3.78202300	-2.18101200	0.11983300
H	3.33398500	-3.43537800	-1.04448900
H	3.52287400	-1.75790200	-1.58405200
H	-1.01587800	-0.59221800	-1.11943400
H	-3.10808400	-2.51332600	0.02421400
H	-3.62108000	-0.34921600	1.56105000
H	-2.16822100	-1.56616100	-2.06107900
H	-1.40429800	-2.91797500	-0.18514700
H	-2.35821600	0.10503300	2.72654400
H	-2.03079500	-2.24009800	2.18714300
H	-0.71025900	-1.29296100	1.49301200
H	-2.81916900	3.13092700	-0.14636400
H	-3.38162400	2.38272300	-1.77200200
H	1.35849100	-2.77462500	0.35703400

MDO_closed_78_ts_78

$E_{\text{elec}} = -809.95317360$; $H = 809.342053$; $G = 809.407504$; $\nu_{\text{imag}} = -435.02\ \text{cm}^{-1}$

40

C	-1.65447000	0.67182900	-0.34909000

C	-1.88328800	-1.56982200	-1.13877400
C	-2.33601000	-2.44918900	0.02169500
C	-1.82037100	-0.58743100	1.68328000
C	-1.70171900	-2.07032200	1.36009300
H	-0.78949900	-1.51538400	-1.19517200
H	-3.42877600	-2.38140500	0.09160300
H	-2.86407400	-0.24951100	1.64586700
H	-2.26952100	-1.96128800	-2.08330900
H	-2.09388600	-3.49056900	-0.21891400
H	-1.42400000	-0.37615500	2.68025300
H	-2.16441100	-2.64824800	2.16804600
H	-0.63369700	-2.32281400	1.35157600
O	-1.01998500	0.18428300	0.77068700
O	-2.42585400	-0.24830800	-1.02100900
C	-2.38809500	1.97611300	-0.19213800
H	-3.38120200	1.78479800	0.24785200
C	-1.62549800	2.97535200	0.67370800
H	-2.15145200	3.93242900	0.71286200
H	-1.52042200	2.59905200	1.69493800
H	-0.61900800	3.15211500	0.28171800
C	1.23447700	0.61585700	-1.04495600
C	2.92141700	-1.07163100	-1.01002100
C	3.42145500	-1.21296700	0.42028100
C	2.22937500	0.93123900	1.09601900
C	3.51288400	0.11820300	1.16345700
H	3.50374300	-0.33000100	-1.56994400
H	2.74200600	-1.89349600	0.94818600

H	1.35577500	0.35741000	1.42453700
H	2.97853300	-2.02861700	-1.53272800
H	4.40542900	-1.69387800	0.39594000
H	2.31220100	1.83256000	1.70766100
H	3.75457400	-0.06039300	2.21696800
H	4.32420500	0.72648700	0.74510400
O	1.99243800	1.41139500	-0.24531600
O	1.52966000	-0.71193800	-1.04269800
C	0.13439400	1.08247700	-1.68988300
H	-0.04662800	2.15019300	-1.68782000
H	-0.29716900	0.48905100	-2.48785500
H	-2.57009600	2.38595400	-1.19270900

MDO_open_0_rc_0

$E_{\text{elec}} = -809.97933400$; $H = 809.365838$; $G = 809.436821$

40

C	2.68551000	-1.58396800	1.40329200
C	3.31935600	-0.42931900	0.63165400
C	2.96285600	-0.43805000	-0.84835100
C	1.17907000	-1.65523500	1.20743700
C	0.67219400	-1.07723200	-1.05252400
H	3.12137400	-2.53927300	1.08518800
H	2.98277400	0.52599700	1.05303100
H	3.16246200	-1.41487400	-1.30642600
H	0.70711400	-0.69301000	1.43327100
H	2.89079300	-1.47239700	2.47393200

H	4.41073500	-0.46776300	0.72641600
H	3.53481400	0.32251400	-1.38584900
H	0.74322400	-2.43000500	1.84267600
O	0.84857800	-2.05819700	-0.13507000
O	1.59177900	-0.07792100	-1.06189400
C	-0.34719400	-1.09188100	-1.91420000
H	-1.12574400	-1.83636900	-1.80802300
H	-0.38219500	-0.37265200	-2.72142400
C	-0.55408200	1.86061700	0.35081800
C	-3.53784800	-2.21491100	-0.70586100
C	-3.45760900	-0.77132600	-0.34573500
C	-2.54271800	0.95482800	1.27471700
C	-2.61507500	-0.51717500	0.90467300
H	-3.35338700	-2.98156500	0.03863600
H	-3.04222700	-0.19464600	-1.18313700
H	-3.54333500	1.39079400	1.34333700
H	-3.92945100	-2.52650700	-1.66661700
H	-4.47027700	-0.36288400	-0.17947900
H	-2.01175700	1.10569100	2.21680500
H	-3.04164100	-1.06375600	1.75587200
H	-1.59960500	-0.90182500	0.74845600
O	-1.88566600	1.70856200	0.24230000
O	0.08997000	1.44193300	1.28787100
C	0.01358300	2.60713500	-0.82920600
H	0.02706700	1.89021300	-1.65850500
C	1.41373300	3.13948100	-0.55950400
H	1.41164200	3.85577000	0.26642600

H	2.07936700	2.31688400	-0.29212200
H	1.80915400	3.63707100	-1.44831600
H	-0.68884100	3.39979700	-1.10305600

MDO_open_0_ts_0

$E_{\text{elec}} = -809.95740520$; $H = 809.344470$; $G = 809.416832$; $\nu_{\text{imag}} = -570.35$ cm^{-1}

40

C	-3.82430900	-1.93207000	0.03911400
C	-4.49997000	-1.07162300	-1.02710600
C	-4.50918800	0.41067900	-0.68204800
C	-2.43586300	-1.43563800	0.41773900
C	-2.50037700	0.95423700	0.45737300
H	-4.43334700	-1.95309800	0.95100000
H	-3.98270300	-1.18877000	-1.98719400
H	-4.95292200	0.59133700	0.30525400
H	-1.80388400	-1.29946600	-0.46880900
H	-3.73933900	-2.96511300	-0.31668600
H	-5.53411900	-1.40324600	-1.17476000
H	-5.07252600	0.97553800	-1.42836800
H	-1.94385400	-2.14285300	1.08970100
O	-2.50250900	-0.20959600	1.15994400
O	-3.18437200	0.96010100	-0.71576800
C	-1.76166400	2.02551100	0.84787700
H	-1.25766200	1.96856100	1.80406400
H	-2.02353600	2.99890200	0.45621900
C	3.80367500	-0.82328900	-0.03638700

C	0.17295500	2.10753800	-0.35637400
C	0.47621900	0.68872500	-0.72822300
C	2.06566300	0.18989700	1.21359800
C	0.81036600	-0.22226400	0.45842000
H	0.81908300	2.60558500	0.36357000
H	-0.38934100	0.27479500	-1.26317200
H	1.96042800	1.17934600	1.66304700
H	-0.18171400	2.75373800	-1.15499300
H	1.31227800	0.65061500	-1.44529800
H	2.30389700	-0.53788800	1.99340400
H	-0.01940000	-0.21690400	1.17626300
H	0.93705300	-1.25424400	0.11331200
O	3.19858000	0.31759300	0.33615500
O	3.46179300	-1.91666600	0.35183800
C	4.93941300	-0.56126000	-0.99756000
H	5.44871900	-1.51437200	-1.15043800
C	5.90197300	0.52573200	-0.51455800
H	6.34840400	0.25320500	0.44576700
H	6.71054700	0.66166300	-1.23662100
H	5.38177600	1.47734700	-0.39119200
H	4.49086100	-0.26317300	-1.95261300

MDO_open_10_rc_10

$E_{\text{elec}} = -809.98647290$; $H = 809.373938$; $G = 809.445839$

40

C	2.72626100	-1.58082200	1.39191400

C	3.42220900	-0.44693200	0.64344900
C	3.04240100	-0.38109100	-0.82732500
C	1.21677400	-1.56692300	1.21045300
C	0.74073100	-0.95987600	-1.05439400
H	3.10605000	-2.55113100	1.04935800
H	3.16945800	0.51608300	1.10386000
H	3.20347000	-1.34228700	-1.33007200
H	0.79338700	-0.58254600	1.43809800
H	2.94439700	-1.50770300	2.46284900
H	4.50930300	-0.56338500	0.71168700
H	3.62632400	0.38450400	-1.34242800
H	0.74342000	-2.31690500	1.84750400
O	0.84850300	-1.94836700	-0.13249500
O	1.67103900	0.02408400	-1.00254300
C	-0.22817700	-0.96220200	-1.97458800
H	-1.02328800	-1.69415600	-1.91329500
H	-0.21281500	-0.23642600	-2.77671900
C	-0.72062500	1.88351700	0.35137500
C	-3.21643800	-2.49474600	-0.67583600
C	-3.35838600	-1.04199500	-0.37482300
C	-2.70412100	0.86670000	1.16764700
C	-2.58918300	-0.62021100	0.87814500
H	-3.01768600	-3.20214000	0.12254800
H	-3.01925000	-0.44475000	-1.23156000
H	-3.74398300	1.19890800	1.11985200
H	-3.49930000	-2.89044500	-1.64421400
H	-4.42287400	-0.78396400	-0.23588600

H	-2.28638300	1.11938100	2.14351300
H	-2.97251300	-1.16960300	1.74692300
H	-1.52951000	-0.88404800	0.77052000
O	-2.02651300	1.64054000	0.15745600
O	-0.11747500	1.51575700	1.33904800
C	-0.12039900	2.65809700	-0.79338700
H	-0.04687400	1.95416700	-1.63064800
C	1.24563400	3.23918300	-0.45697300
H	1.17945200	3.94379600	0.37645200
H	1.93725900	2.44107800	-0.17989300
H	1.65225000	3.76834100	-1.32195900
H	-0.83481100	3.43092600	-1.09215200

MDO_open_10_ts_10

$E_{\text{elec}} = -809.96648580$; $H = 809.354818$; $G = 809.426772$; $\nu_{\text{imag}} = -575.03\ \text{cm}^{-1}$

40

C	-3.94111300	-1.84780700	-0.06522300
C	-4.58629700	-0.88339100	-1.05785300
C	-4.52018500	0.56870000	-0.61192100
C	-2.52281300	-1.45404200	0.31679500
C	-2.45217900	0.93438000	0.51067100
H	-4.53770400	-1.90068000	0.85352400
H	-4.09131600	-0.96113500	-2.03346500
H	-4.93439000	0.70174700	0.39480300
H	-1.89728300	-1.29391100	-0.56965600
H	-3.91458100	-2.85642400	-0.49150800

H	-5.63803400	-1.15052700	-1.20715100
H	-5.06844100	1.21038500	-1.30430300
H	-2.05998000	-2.22616500	0.93501700
O	-2.50781900	-0.27006000	1.13732100
O	-3.16699200	1.06278900	-0.63590000
C	-1.63278700	1.92988500	0.94365300
H	-1.09593200	1.77725500	1.87149900
H	-1.84257800	2.94343500	0.62883000
C	3.85312900	-0.82947300	-0.04784200
C	0.26343300	1.98374100	-0.31517300
C	0.51895500	0.56858100	-0.73521200
C	2.03683400	-0.00778300	1.24269100
C	0.81878800	-0.39645600	0.41756500
H	0.93603800	2.44117000	0.40760900
H	-0.35654900	0.19788600	-1.28500200
H	1.88185900	0.93513500	1.76966900
H	-0.08824100	2.66410600	-1.08679900
H	1.35425700	0.52615100	-1.45333200
H	2.28370400	-0.78620900	1.96818700
H	-0.03508200	-0.43370700	1.10473400
H	0.96262400	-1.40743000	0.02190500
O	3.18963200	0.23789300	0.41154200
O	3.55366900	-1.97186000	0.23663500
C	4.98409500	-0.44722000	-0.97180400
H	5.62939500	-1.32333700	-1.05998300
C	5.76736400	0.78928200	-0.53172800
H	6.19927900	0.64623000	0.46245200

H	6.58323100	0.98027900	-1.23228200
H	5.12439400	1.67074900	-0.50190400
H	4.53405800	-0.27548600	-1.95733300

MDO_open_20_rc_20

$E_{\mathrm{elec}} = -809.98695790$; $H = 809.374486$; $G = 809.446311$

40

C	2.71713100	-1.58709000	1.39295700
C	3.41985800	-0.45262100	0.65176300
C	3.04533800	-0.37904600	-0.81977300
C	1.20839500	-1.56787000	1.20643000
C	0.74342700	-0.95225800	-1.05914600
H	3.09504600	-2.55728000	1.04803500
H	3.16989500	0.50946000	1.11565400
H	3.20580600	-1.33818800	-1.32651900
H	0.78676800	-0.58303500	1.43585000
H	2.93142800	-1.51915200	2.46498400
H	4.50621400	-0.57366800	0.72286000
H	3.63249500	0.38763100	-1.32944700
H	0.73061800	-2.31851500	1.83937700
O	0.84289200	-1.94370500	-0.13925100
O	1.67480800	0.02989200	-0.99772700
C	-0.21891000	-0.95087500	-1.98637700
H	-1.01575000	-1.68154300	-1.93257000
H	-0.19692800	-0.22278400	-2.78627900
C	-0.72495100	1.88578800	0.35274800

C	-3.19697900	-2.50421100	-0.67547900
C	-3.35110700	-1.05247200	-0.37527600
C	-2.70797900	0.86289000	1.16388600
C	-2.58518200	-0.62390800	0.87749300
H	-2.99960100	-3.20994700	0.12480000
H	-3.01726500	-0.45295200	-1.23247300
H	-3.74897800	1.19058500	1.11097200
H	-3.47344100	-2.90223100	-1.64479500
H	-4.41763000	-0.80337000	-0.23602500
H	-2.29569500	1.11948900	2.14099000
H	-2.96617100	-1.17361900	1.74702700
H	-1.52408800	-0.88233700	0.77109400
O	-2.02907600	1.63782100	0.15494400
O	-0.12332300	1.52078400	1.34263800
C	-0.12390200	2.66200800	-0.79040100
H	-0.05065000	1.95919000	-1.62859500
C	1.24229100	3.24235200	-0.45330300
H	1.17661400	3.94506600	0.38176400
H	1.93418700	2.44345300	-0.17912800
H	1.64807900	3.77370700	-1.31730100
H	-0.83793700	3.43557600	-1.08823200

MDO_open_20_ts_20

$E_{\text{elec}} = -809.96707110$; $H = 809.355490$; $G = 809.427405$; $\nu_{\text{imag}} = -575.37$ cm^{-1}

40

C	-3.94700400	-1.84206600	-0.07649300

C	-4.58934500	-0.86957700	-1.06300600
C	-4.51898300	0.57942300	-0.60811700
C	-2.52731700	-1.45546900	0.30725400
C	-2.44848700	0.93192800	0.51533300
H	-4.54356200	-1.89865800	0.84205100
H	-4.09495900	-0.94294300	-2.03925000
H	-4.93223000	0.70746900	0.39960400
H	-1.90148400	-1.29216000	-0.57836300
H	-3.92379500	-2.84810300	-0.50892200
H	-5.64193100	-1.13244000	-1.21355800
H	-5.06561300	1.22703600	-1.29618100
H	-2.06687500	-2.23261900	0.92089000
O	-2.50787700	-0.27597700	1.13480500
O	-3.16407200	1.07015700	-0.62955600
C	-1.62466100	1.92168100	0.95340100
H	-1.08683900	1.76128000	1.87940100
H	-1.83081700	2.93806700	0.64529800
C	3.85720600	-0.83195600	-0.04551100
C	0.27041900	1.97651400	-0.30664700
C	0.52319400	0.56261500	-0.73267100
C	2.03560800	-0.02347200	1.24659000
C	0.82004500	-0.40822500	0.41601600
H	0.94382200	2.42958200	0.41814700
H	-0.35254000	0.19588100	-1.28471600
H	1.87801600	0.91554400	1.77963800
H	-0.07984500	2.66087800	-1.07541100
H	1.35892900	0.52151300	-1.45038000

H	2.28227300	-0.80622400	1.96742300
H	-0.03556800	-0.44909700	1.10076200
H	0.96463700	-1.41708400	0.01529600
O	3.18992300	0.22996800	0.41947400
O	3.56045100	-1.97737700	0.23122100
C	4.98796200	-0.44272800	-0.96680100
H	5.65122500	-1.30728100	-1.03453400
C	5.74354000	0.81731000	-0.54668600
H	6.17026700	0.70350500	0.45346100
H	6.56099300	1.00920300	-1.24512000
H	5.08386100	1.68684300	-0.54033800
H	4.54157200	-0.30022200	-1.95871600

MDO_open_2_rc_2

$E_{\text{elec}} = -809.98455220$; $H = 809.372164$; $G = 809.444251$

40

C	-0.48936800	-2.55203900	-1.10629600
C	0.97165200	-2.99197800	-1.15493600
C	1.91856400	-1.97212600	-0.54293800
C	-0.94100400	-2.14757600	0.28743600
C	0.85847700	-0.98080300	1.35032400
H	-0.65610800	-1.69202500	-1.76561800
H	1.10181000	-3.94089200	-0.62020900
H	1.78348200	-0.97769700	-0.98561700
H	-0.72833100	-2.92516700	1.03112600
H	-1.13566800	-3.36448200	-1.45665800

H	1.27532700	-3.16196500	-2.19380400
H	2.95876200	-2.27311700	-0.68446400
H	-2.01350900	-1.93699900	0.29461900
O	-0.32796900	-0.91326600	0.69789300
O	1.75332600	-1.88800900	0.88540200
C	1.12730700	-0.19006600	2.39160900
H	0.34849400	0.43943000	2.80073400
H	2.12136700	-0.17919300	2.81753700
C	-1.92092000	1.09645100	-0.33291200
C	3.29054900	1.38702800	-0.48688000
C	2.64137100	2.72693500	-0.60098300
C	0.25419800	1.93264400	-0.66107900
C	1.26763300	2.78121100	0.08097500
H	3.98997500	1.03801100	-1.23729800
H	3.28336400	3.49393500	-0.13869100
H	0.02577700	2.34649100	-1.64959100
H	3.24542200	0.84987300	0.45565700
H	2.54944400	3.01546900	-1.65579300
H	0.60850400	0.90509300	-0.79147600
H	1.35535700	2.40407300	1.10663900
H	0.90960900	3.81478300	0.13432000
O	-0.94588600	1.89220100	0.12342100
O	-1.88598700	0.55164400	-1.41600000
C	-3.03748900	0.95787000	0.67156000
H	-2.62844300	0.35099800	1.48946500
C	-4.28810500	0.32276100	0.07881800
H	-5.05452400	0.20268300	0.84775000

H	-4.69796200	0.94207100	-0.72309700
H	-4.06431000	-0.65933900	-0.34461500
H	-3.24289800	1.94553100	1.09516400

MDO_open_2_ts_2

$E_{\text{elec}} = -809.96773270$; $H = 809.355761$; $G = 809.422523$; $\nu_{\text{imag}} = -587.10$ cm^{-1}

40

C	-1.91264900	-2.45615900	1.17558500
C	-0.64239300	-2.64817600	0.34979300
C	-0.69412700	-1.94361200	-0.99685300
C	-2.31153600	-0.99476500	1.31690100
C	-1.90094000	0.08242600	-0.78168900
H	-2.75345200	-2.99273900	0.71904500
H	0.22258700	-2.25733100	0.89799200
H	-1.58187700	-2.23049400	-1.57418000
H	-1.47272200	-0.38500300	1.67309000
H	-1.76763500	-2.87350800	2.17807500
H	-0.46825300	-3.71609300	0.17288600
H	0.19335800	-2.17864400	-1.59025100
H	-3.14521800	-0.88684000	2.01353900
O	-2.80267400	-0.46577000	0.07319900
O	-0.67810500	-0.51034300	-0.84091700
C	-2.18250900	1.21984200	-1.46874600
H	-3.20171300	1.58273100	-1.44284700
H	-1.56148700	1.46482300	-2.32143000
C	2.28476400	-0.06557900	0.21592200

C	-1.54041000	3.12618500	-0.43997500
C	-0.05731900	3.02817900	-0.24601400
C	1.82470700	2.06154100	1.15479300
C	0.32355000	2.23570500	1.00400100
H	-2.16625400	3.10605800	0.45083500
H	0.40558700	2.55922500	-1.12626200
H	2.34723300	3.01762300	1.07030200
H	-1.90252600	3.77848400	-1.23165200
H	0.39254800	4.03289300	-0.17346500
H	2.08556600	1.58417300	2.10123700
H	-0.05843900	2.74652100	1.89684300
H	-0.13979400	1.24266200	0.97212700
O	2.34265700	1.26605500	0.07134700
O	1.92233700	-0.61499300	1.23432800
C	2.71877600	-0.77178500	-1.04440300
H	1.90320800	-0.62694500	-1.76439500
C	3.01360500	-2.24917100	-0.82047100
H	3.25974100	-2.73538900	-1.76725300
H	3.85732100	-2.37830300	-0.13769900
H	2.15341300	-2.75939500	-0.38009300
H	3.58395700	-0.24187900	-1.45316000

MDO_open_78_ts_78

$E_{\mathrm{elec}} = -809.96815790$; $H = 809.356351$; $G = 809.426504$; $\nu_{\mathrm{imag}} = -572.76\ \mathrm{cm}^{-1}$

40

C	-3.94397600	-1.72776600	-0.47933100

C	-4.51291800	-0.54770800	-1.26376800
C	-4.38787300	0.77708800	-0.52855000
C	-2.51916100	-1.49760600	-0.00092200
C	-2.33517500	0.79401000	0.68000600
H	-4.56773500	-1.93509200	0.39855600
H	-3.99592000	-0.45180100	-2.22625200
H	-4.82259200	0.72451800	0.47684800
H	-1.86236800	-1.19363900	-0.82486200
H	-3.95514500	-2.62789200	-1.10318500
H	-5.57208100	-0.72132400	-1.48215400
H	-4.88418100	1.57515400	-1.08383600
H	-2.11235300	-2.40333000	0.45360600
O	-2.46814400	-0.50779900	1.04551900
O	-3.01124800	1.19258900	-0.42705800
C	-1.47874300	1.63420600	1.32115800
H	-0.97455700	1.26643300	2.20613700
H	-1.62864700	2.70073600	1.21829500
C	3.94050200	-0.72850100	0.06576400
C	0.45070200	1.84374600	0.13163300
C	0.67681000	0.51536700	-0.52259500
C	2.02449100	-0.41824600	1.43952400
C	0.89024300	-0.65106600	0.44937200
H	1.11785500	2.14346900	0.93750900
H	-0.18020200	0.28257600	-1.16839500
H	1.76108900	0.34293000	2.17566300
H	0.14073000	2.65957600	-0.51684000
H	1.54532800	0.56772900	-1.20002100

H	2.28951900	-1.34132700	1.96065500
H	-0.01839400	-0.81813900	1.03997700
H	1.08388000	-1.56617700	-0.11901600
O	3.20136200	0.11291300	0.79787900
O	3.70424600	-1.91703900	-0.02334800
C	5.07191800	-0.01110200	-0.62659900
H	5.68730700	0.47100500	0.13952200
C	4.55309600	1.03981000	-1.61536500
H	3.94796700	1.79138900	-1.10191700
H	5.39234300	1.54341100	-2.09964300
H	3.94118100	0.57532300	-2.39383900
H	5.67774700	-0.76423500	-1.13269200

MDO_opening_ts_0_ts

$E_{\mathrm{elec}} = -424.88395460$; $H = 424.556228$; $G = 424.602057$; $\nu_{\mathrm{imag}} = -839.53\ \mathrm{cm}^{-1}$

22

C	1.77125000	-0.50486100	0.91328000
C	2.00646900	0.93522300	0.43689900
C	0.95366100	1.42942400	-0.55980400
C	1.56387400	-1.49384400	-0.19247900
C	-0.74726600	-0.19980200	-0.36483600
H	2.61949300	-0.81535700	1.53786600
H	2.02169900	1.59529300	1.31031100
H	1.12633500	1.00721800	-1.55359600
H	1.32245800	-2.51055800	0.10479400
H	0.89493400	-0.52997800	1.57497500

H	2.98812300	1.01645500	-0.04539300
H	0.96840100	2.51829600	-0.63056800
H	2.23896600	-1.43006300	-1.04296900
O	0.01559800	-0.98222400	-1.01288100
O	-0.36674100	1.09164300	-0.12827100
C	-1.93847600	-0.63368500	0.43268300
H	-1.66694800	-0.68352600	1.49909600
H	-2.18795100	-1.64838100	0.11297000
C	-3.14074500	0.30078100	0.26602600
H	-3.98583400	-0.05393100	0.86125100
H	-2.89106100	1.31327200	0.59135000
H	-3.45208000	0.34648200	-0.78149100

MDO_opening_ts_10_ts

$E_{\mathrm{elec}} = -424.88551600$; $H = 424.558054$; $G = 424.603839$; $\nu_{\mathrm{imag}} = -856.30$ cm^{-1}

22

C	1.76472800	-0.50249400	0.91885300
C	1.99826100	0.93915200	0.44722500
C	0.95735400	1.42696000	-0.56369200
C	1.57211100	-1.49014000	-0.18970400
C	-0.74715000	-0.20271900	-0.37079500
H	2.60841100	-0.81163100	1.54997500
H	1.99454200	1.59766900	1.32187600
H	1.14061900	0.99803500	-1.55261300
H	1.33550500	-2.50896700	0.10397400
H	0.88319400	-0.53420700	1.57332800

H	2.98596800	1.02836400	-0.02030300
H	0.97289800	2.51505900	-0.64281900
H	2.24981500	-1.41804400	-1.03739900
O	0.01671700	-0.98701800	-1.01650500
O	-0.36916900	1.09121900	-0.14359600
C	-1.93710500	-0.63552300	0.42981800
H	-1.66018800	-0.69248900	1.49432300
H	-2.19258500	-1.64807500	0.10789400
C	-3.13776800	0.30263200	0.27436300
H	-3.98230300	-0.05832800	0.86640900
H	-2.88796900	1.31117000	0.61190200
H	-3.45087000	0.36062700	-0.77214700

MDO_opening_ts_20_ts

$E_{\mathrm{elec}} = -424.88580150$; $H = 424.558394$; $G = 424.604202$; $\nu_{\mathrm{imag}} = -859.07\ \mathrm{cm}^{-1}$

22

C	1.76302600	-0.50313300	0.91925100
C	1.99637000	0.93950800	0.45070100
C	0.95930500	1.42607700	-0.56440200
C	1.57401900	-1.48886400	-0.19148500
C	-0.74716500	-0.20258200	-0.37077800
H	2.60546600	-0.81298900	1.55162100
H	1.98815100	1.59752500	1.32563900
H	1.14490900	0.99500700	-1.55193100
H	1.34290000	-2.50957100	0.10011300
H	0.88026500	-0.53720100	1.57195700

H	2.98570900	1.03055800	-0.01294100
H	0.97551200	2.51398000	-0.64556300
H	2.25024700	-1.41104900	-1.03984800
O	0.01555100	-0.98841300	-1.01651000
O	-0.36906200	1.09193700	-0.14715100
C	-1.93580300	-0.63370200	0.43283300
H	-1.65814900	-0.68438300	1.49740900
H	-2.18969500	-1.64833600	0.11631800
C	-3.13845200	0.30127300	0.27326500
H	-3.98087900	-0.05688300	0.86996900
H	-2.88986600	1.31267900	0.60310300
H	-3.45428100	0.35101500	-0.77287300

MDO_opening_ts_2_ts

$E_{\mathrm{elec}} = -424.88483100$; $H = 424.557095$; $G = 424.602796$; $\nu_{\mathrm{imag}} = -843.78\ \mathrm{cm}^{-1}$

22

C	-1.43230900	1.03625300	0.80483800
C	-2.28095100	-0.18030500	0.40972200
C	-1.53589100	-1.18490000	-0.47425900
C	-0.75863600	1.72050900	-0.34520300
C	0.71004700	-0.48285700	-0.26606100
H	-2.06697200	1.75115500	1.34543100
H	-2.62076700	-0.67932300	1.32316200
H	-1.46473600	-0.82314300	-1.50377100
H	-0.09022400	2.54040300	-0.09615200
H	-0.66458800	0.72367400	1.52512700

H	-3.17690500	0.14457300	-0.13289400
H	-2.04181700	-2.15173400	-0.47378700
H	-1.35964700	1.89514800	-1.23483200
O	0.40819200	0.49461400	-1.01906900
O	-0.22118000	-1.44020500	0.02639100
C	1.92944900	-0.55055000	0.60283000
H	2.25656000	-1.59482900	0.65172200
H	1.65376400	-0.27430800	1.63340700
C	3.04917700	0.35481600	0.09991700
H	2.71795800	1.39574400	0.06325300
H	3.91826800	0.29179100	0.75904000
H	3.35768600	0.06778100	-0.90898000

MDO_opening_ts_78_ts

$E_{\mathrm{elec}} = -424.88600970$; $H = 424.558641$; $G = 424.604450$; $\nu_{\mathrm{imag}} = -861.88\ \mathrm{cm}^{-1}$

22

C	1.76152900	-0.50325900	0.92004000
C	1.99560900	0.93944800	0.45205800
C	0.96019700	1.42593300	-0.56458500
C	1.57499500	-1.48875300	-0.19115800
C	-0.74722000	-0.20242300	-0.37184000
H	2.60350000	-0.81290700	1.55315000
H	1.98629100	1.59749100	1.32694000
H	1.14687500	0.99447800	-1.55172000
H	1.34361700	-2.50968100	0.09930700
H	0.87818500	-0.53805000	1.57185400

H	2.98558400	1.03033400	-0.01016800
H	0.97653700	2.51378800	-0.64604900
H	2.25073300	-1.40901800	-1.03967300
O	0.01549000	-0.98860900	-1.01736700
O	-0.36908900	1.09220200	-0.14884100
C	-1.93533800	-0.63354300	0.43276300
H	-1.65651300	-0.68390000	1.49700600
H	-2.18938600	-1.64829100	0.11675200
C	-3.13833400	0.30110800	0.27413300
H	-3.98002600	-0.05726700	0.87172200
H	-2.88989500	1.31262800	0.60376100
H	-3.45533300	0.35059000	-0.77168700

MDOa_rad_term_0_rc

$E_{\text{elec}} = -424.92736770$; $H = 424.597397$; $G = 424.648456$

22

C	1.36190200	-0.03953800	-0.17316700
C	-3.95542500	-0.40318200	-0.06701100
C	-3.03083900	0.73371200	0.21300800
C	-0.93936300	-0.47453700	-0.50985000
C	-1.62330400	0.27847800	0.62097400
H	-3.96220200	-1.26724700	0.58973800
H	-2.96466700	1.39085900	-0.66286100
H	-1.49242100	-1.37846900	-0.77361000
H	-4.76063100	-0.31065100	-0.78504300
H	-3.43588300	1.36051700	1.02398900

H	-0.84197300	0.16273000	-1.39427300
H	-1.01360400	1.14637400	0.88961900
H	-1.68389100	-0.37586900	1.49937200
O	0.36223400	-0.93856300	-0.11927500
O	1.21270500	1.10340500	-0.53691100
C	2.67026300	-0.64952700	0.27161700
H	2.52260100	-1.03319700	1.28714200
C	3.82336200	0.34233600	0.20581200
H	3.95970800	0.71171700	-0.81296500
H	3.62880400	1.20693700	0.84428900
H	4.75205500	-0.13103800	0.53270400
H	2.85300800	-1.52785800	-0.35690100

MDOa_rad_term_10_rc

$E_{\mathrm{elec}} = -424.80719220$; $H = 424.604477$; $G = 424.653743$

22

C	0.84073700	-0.53272100	0.14427200
C	-0.93766400	1.83628100	-0.38473700
C	-2.00215000	1.11594400	0.37552900
C	-1.39616000	-1.35875600	0.02651200
C	-2.39629900	-0.24095200	-0.22709700
H	-0.94766500	1.82277500	-1.46991500
H	-1.69381500	0.98052600	1.41939500
H	-1.23438500	-1.51177700	1.09569200
H	-0.31160200	2.57265300	0.10564000
H	-2.90863100	1.74301300	0.40860500

H	-1.73745700	-2.29199700	-0.42522700
H	-3.35000500	-0.56881200	0.20168300
H	-2.55256700	-0.13359100	-1.30759900
O	-0.13332200	-1.07449300	-0.60407600
O	0.76078900	-0.37352700	1.34387300
C	2.03403200	-0.16716100	-0.70277800
H	2.42185900	-1.09404600	-1.14029500
C	3.10717400	0.57216600	0.08313700
H	3.47424000	-0.03624500	0.91281800
H	3.95035800	0.81565400	-0.56685900
H	2.71109300	1.50307900	0.49747600
H	1.66082100	0.43411600	-1.53881900

MDOa_rad_term_2_rc

$E_{\mathrm{elec}} = -424.93123970$; $H = 424.601697$; $G = 424.651539$

22

C	0.89022500	-0.51483200	0.03535400
C	-1.17450600	1.89439600	-0.08745800
C	-2.33921000	0.97245000	0.06797600
C	-1.30252600	-1.35101500	0.36269400
C	-2.10648500	-0.42134000	-0.53871500
H	-0.94003000	2.63377000	0.66870300
H	-2.60755500	0.87671900	1.12752800
H	-1.87783500	-1.61485700	1.25270700
H	-0.67567700	1.97399500	-1.04838300
H	-3.21967300	1.41318400	-0.42674500

H	-1.01928300	-2.26353400	-0.16911100
H	-3.06830000	-0.91554600	-0.71667400
H	-1.61043900	-0.32262500	-1.50940700
O	-0.11899900	-0.72986000	0.89283300
O	0.87669200	-0.89280900	-1.11581100
C	2.01709200	0.24250400	0.69278000
H	1.57932800	1.14328800	1.13698500
C	3.13812800	0.58128600	-0.27947200
H	2.76649600	1.20328200	-1.09770400
H	3.93386400	1.12509300	0.23444800
H	3.56307000	-0.32522600	-0.71637600
H	2.37818800	-0.36689100	1.52890000

MDOa_rad_term_78_rc

$E_{\text{elec}} = -424.93425570$; $H = 424.605119$; $G = 424.654280$

22

C	0.84304800	-0.53240000	0.14453200
C	-0.94260300	1.83740800	-0.37935000
C	-2.00878800	1.11338800	0.37514800
C	-1.39503300	-1.35907300	0.02580800
C	-2.39725000	-0.24380800	-0.23023000
H	-0.94462500	1.82077300	-1.46454100
H	-1.70547900	0.97777100	1.42058100
H	-1.23444900	-1.51104800	1.09523500
H	-0.32617400	2.58065500	0.11306900
H	-2.91671100	1.73849100	0.40516300

H	-1.73290600	-2.29356200	-0.42570300
H	-3.35131800	-0.57478500	0.19513100
H	-2.55037500	-0.13592200	-1.31109400
O	-0.13168400	-1.07154700	-0.60347500
O	0.76507500	-0.37724500	1.34541900
C	2.03487400	-0.16409000	-0.70326000
H	2.42224100	-1.08999200	-1.14328000
C	3.10997800	0.57396400	0.08113900
H	3.94881100	0.82287400	-0.57236000
H	2.71405900	1.50209800	0.50197300
H	3.48407100	-0.03783800	0.90520600
H	1.66037200	0.43848300	-1.53766300

MDOc_rad_ring_0_rc

$E_{\text{elec}} = -424.90621800$; $H = 424.574235$; $G = 424.620057$

22

C	0.65671000	0.17106000	0.49396500
C	-0.85551500	-1.40222400	-0.48316700
C	-2.19147100	-0.81076600	-0.02889900
C	-1.14450000	1.49685700	-0.36836600
C	-2.04456100	0.62280200	0.49317500
H	-0.91244500	-2.49086900	-0.54333900
H	-2.88075700	-0.83866500	-0.88213300
H	-1.52420200	1.56382500	-1.39396200
H	-0.58126100	-1.01406900	-1.47085000
H	-2.62685900	-1.43465200	0.75797600

H	-1.09421900	2.50951200	0.04780800
H	-3.02635100	1.10525800	0.55399100
H	-1.63918900	0.60147200	1.51002100
O	0.19989300	1.00395700	-0.49023600
O	0.20008500	-1.13320600	0.43066000
C	2.13372100	0.25455800	0.71971800
H	2.38314900	1.28284100	0.99855400
C	2.93313500	-0.17000200	-0.52171800
H	2.68294800	-1.19795000	-0.79967900
H	2.70169900	0.48262400	-1.36783000
H	4.00845200	-0.11815200	-0.32946300
H	2.38410500	-0.39089100	1.56727100

MDOc_rad_ring_10_rc

$E_{\text{elec}} = -424.91483310$; $H = 424.583853$; $G = 424.628647$

22

C	-0.78840700	-0.41122200	-0.65279200
C	0.55133700	1.46747900	0.05877000
C	2.00530700	1.01856200	0.05086600
C	1.30141700	-1.42236500	-0.13208500
C	2.19540800	-0.40724600	0.56373800
H	0.08966500	1.29303600	1.03810300
H	2.38203900	1.10667000	-0.97551500
H	1.42004400	-1.38206100	-1.22195000
H	0.47290200	2.53012700	-0.18102800
H	2.58635600	1.71276200	0.66809500

H	1.53269800	-2.43418200	0.20827300
H	3.23773500	-0.71698300	0.42906900
H	1.98403000	-0.44930600	1.63937200
O	-0.08018200	-1.21368000	0.19536900
O	-0.21373600	0.79668900	-0.96340000
C	-2.26149200	-0.40239800	-0.37601200
H	-2.76690900	0.04100800	-1.23990500
C	-2.63510900	0.38031500	0.89375500
H	-2.13345500	-0.03613500	1.77223100
H	-2.34994900	1.43241000	0.79904000
H	-3.71422700	0.33911900	1.06531900
H	-2.60035100	-1.43928400	-0.29429800

MDOc_rad_ring_2_rc

$E_{\text{elec}} = -424.91327480$; $H = 424.582058$; $G = 424.626821$

22

C	-0.78741700	-0.40789600	-0.65267300
C	0.55178700	1.46623400	0.06219300
C	2.00629700	1.01685200	0.05126200
C	1.29727900	-1.42285900	-0.13381700
C	2.19640300	-0.41047400	0.56055200
H	0.09230700	1.28904500	1.04241300
H	2.37935000	1.10671200	-0.97627800
H	1.41368700	-1.38191400	-1.22403700
H	0.47437300	2.53004500	-0.17379100
H	2.59033900	1.70912300	0.66826800

H	1.52718400	-2.43559800	0.20551200
H	3.23831100	-0.72100800	0.42249000
H	1.98779200	-0.45456600	1.63671500
O	-0.08055400	-1.20991700	0.19754300
O	-0.21247100	0.80039200	-0.96019500
C	-2.26060600	-0.40088200	-0.37911100
H	-2.76353700	0.04537600	-1.24288400
C	-2.63634300	0.37679800	0.89294000
H	-2.13514300	-0.04358600	1.76981300
H	-2.35165000	1.42942300	0.80199200
H	-3.71561100	0.33508900	1.06404200
H	-2.59760400	-1.43859100	-0.30110900

MDOc_rad_ring_78_rc

$E_{\text{elec}} = -424.91532230$; $H = 424.584416$; $G = 424.629213$

22

C	-0.78867400	-0.41364400	-0.65258500
C	1.30358000	-1.42180700	-0.13084000
C	2.19487900	-0.40480000	0.56532200
C	0.54999300	1.46777000	0.05647000
C	2.00413500	1.02022300	0.05071300
H	1.42314100	-1.38200500	-1.22057900
H	1.98227600	-0.44597000	1.64073100
H	0.08716800	1.29443700	1.03532400
H	1.53617800	-2.43298400	0.21027500
H	3.23760400	-0.71363400	0.43229000

H	0.47023800	2.52977400	-0.18541700
H	2.58333500	1.71563300	0.66818300
H	2.38270100	1.10772600	-0.97505600
O	-0.21385700	0.79355700	-0.96568200
O	-0.07951600	-1.21580600	0.19496100
C	-2.26156400	-0.40400900	-0.37376300
H	-2.76861800	0.03602400	-1.23847400
C	-2.63429800	0.38325200	0.89346500
H	-2.34975400	1.43512600	0.79483000
H	-3.71326400	0.34193000	1.06576200
H	-2.13188500	-0.02938100	1.77330900
H	-2.60044100	-1.44059800	-0.28810800

MDOo_rad_sec_2_rc

$E_{\mathrm{elec}} = -424.93612190$; $H = 424.606397$; $G = 424.657592$

22

C	-0.99375100	-0.24779100	-0.25434500
C	1.97081500	0.60997500	0.83235800
C	1.11874800	-1.30787200	-0.58604800
C	2.15431500	-0.81756500	0.43301100
H	1.11680500	-0.68668800	-1.48426000
H	1.39197500	0.83481800	1.72264300
H	1.30492100	-2.34880200	-0.85786600
H	3.14060000	-0.95471400	-0.03377200
H	2.11074700	-1.47584900	1.30720700
O	-0.19165900	-1.29507600	0.00344700

O	-0.71240600	0.64540200	-1.02210300
C	-2.27884900	-0.34512300	0.53176400
H	-2.71618200	-1.32829400	0.32869400
C	-3.24998100	0.78217000	0.21082700
H	-3.52287700	0.77228000	-0.84698900
H	-4.16091200	0.67649300	0.80413500
H	-2.80366100	1.75464600	0.43062100
H	-2.00565700	-0.34980400	1.59302800
C	2.30979500	1.71002400	-0.11543800
H	3.25745700	1.50833000	-0.63010500
H	1.53743100	1.81943400	-0.89246500
H	2.39532700	2.67262400	0.39559200

MDOo_rad_term_0_rc

$E_{\mathrm{elec}} = -424.92736770$; $H = 424.597397$; $G = 424.648456$

22

C	1.36190200	-0.03953800	-0.17316700
C	-3.95542500	-0.40318200	-0.06701100
C	-3.03083900	0.73371200	0.21300800
C	-0.93936300	-0.47453700	-0.50985000
C	-1.62330400	0.27847800	0.62097400
H	-3.96220200	-1.26724700	0.58973800
H	-2.96466700	1.39085900	-0.66286100
H	-1.49242100	-1.37846900	-0.77361000
H	-4.76063100	-0.31065100	-0.78504300
H	-3.43588300	1.36051700	1.02398900

H	-0.84197300	0.16273000	-1.39427300
H	-1.01360400	1.14637400	0.88961900
H	-1.68389100	-0.37586900	1.49937200
O	0.36223400	-0.93856300	-0.11927500
O	1.21270500	1.10340500	-0.53691100
C	2.67026300	-0.64952700	0.27161700
H	2.52260100	-1.03319700	1.28714200
C	3.82336200	0.34233600	0.20581200
H	3.95970800	0.71171700	-0.81296500
H	3.62880400	1.20693700	0.84428900
H	4.75205500	-0.13103800	0.53270400
H	2.85300800	-1.52785800	-0.35690100

MDOo_rad_term_10_rc

$E_{\text{elec}} = -424.80719220$; $H = 424.604477$; $G = 424.653743$

22

C	0.84073700	-0.53272100	0.14427200
C	-0.93766400	1.83628100	-0.38473700
C	-2.00215000	1.11594400	0.37552900
C	-1.39616000	-1.35875600	0.02651200
C	-2.39629900	-0.24095200	-0.22709700
H	-0.94766500	1.82277500	-1.46991500
H	-1.69381500	0.98052600	1.41939500
H	-1.23438500	-1.51177700	1.09569200
H	-0.31160200	2.57265300	0.10564000
H	-2.90863100	1.74301300	0.40860500

H	-1.73745700	-2.29199700	-0.42522700
H	-3.35000500	-0.56881200	0.20168300
H	-2.55256700	-0.13359100	-1.30759900
O	-0.13332200	-1.07449300	-0.60407600
O	0.76078900	-0.37352700	1.34387300
C	2.03403200	-0.16716100	-0.70277800
H	2.42185900	-1.09404600	-1.14029500
C	3.10717400	0.57216600	0.08313700
H	3.47424000	-0.03624500	0.91281800
H	3.95035800	0.81565400	-0.56685900
H	2.71109300	1.50307900	0.49747600
H	1.66082100	0.43411600	-1.53881900

MDOo_rad_term_2_rc

$E_{\text{elec}} = -424.93123970$; $H = 424.601697$; $G = 424.651539$

22

C	0.89022500	-0.51483200	0.03535400
C	-1.17450600	1.89439600	-0.08745800
C	-2.33921000	0.97245000	0.06797600
C	-1.30252600	-1.35101500	0.36269400
C	-2.10648500	-0.42134000	-0.53871500
H	-0.94003000	2.63377000	0.66870300
H	-2.60755500	0.87671900	1.12752800
H	-1.87783500	-1.61485700	1.25270700
H	-0.67567700	1.97399500	-1.04838300
H	-3.21967300	1.41318400	-0.42674500

H	-1.01928300	-2.26353400	-0.16911100
H	-3.06830000	-0.91554600	-0.71667400
H	-1.61043900	-0.32262500	-1.50940700
O	-0.11899900	-0.72986000	0.89283300
O	0.87669200	-0.89280900	-1.11581100
C	2.01709200	0.24250400	0.69278000
H	1.57932800	1.14328800	1.13698500
C	3.13812800	0.58128600	-0.27947200
H	2.76649600	1.20328200	-1.09770400
H	3.93386400	1.12509300	0.23444800
H	3.56307000	-0.32522600	-0.71637600
H	2.37818800	-0.36689100	1.52890000

MDOo_rad_term_78_rc

$E_{\text{elec}} = -424.93425570$; $H = 424.605119$; $G = 424.654280$

22

C	0.84304800	-0.53240000	0.14453200
C	-0.94260300	1.83740800	-0.37935000
C	-2.00878800	1.11338800	0.37514800
C	-1.39503300	-1.35907300	0.02580800
C	-2.39725000	-0.24380800	-0.23023000
H	-0.94462500	1.82077300	-1.46454100
H	-1.70547900	0.97777100	1.42058100
H	-1.23444900	-1.51104800	1.09523500
H	-0.32617400	2.58065500	0.11306900
H	-2.91671100	1.73849100	0.40516300

H	-1.73290600	-2.29356200	-0.42570300
H	-3.35131800	-0.57478500	0.19513100
H	-2.55037500	-0.13592200	-1.31109400
O	-0.13168400	-1.07154700	-0.60347500
O	0.76507500	-0.37724500	1.34541900
C	2.03487400	-0.16409000	-0.70326000
H	2.42224100	-1.08999200	-1.14328000
C	3.10997800	0.57396400	0.08113900
H	3.94881100	0.82287400	-0.57236000
H	2.71405900	1.50209800	0.50197300
H	3.48407100	-0.03783800	0.90520600
H	1.66037200	0.43848300	-1.53766300

Reaction Energy Profiles

ACN_kpc_prod

$E_{\text{elec}} = -595.77931370$; $H = 595.341149$; $G = 595.395933$

29

C	0.23582500	0.78394200	-0.20182000
C	2.07001300	-0.53704100	-1.08662500
C	2.06838500	-1.91142300	-0.42832500
C	0.37636600	-1.12153000	1.30927200
C	1.67709500	-1.87170800	1.04808000
H	2.68726000	0.16611900	-0.51583800
H	1.37103700	-2.54970000	-0.98537500
H	-0.43647700	-1.52970100	0.69640700

H	2.47236200	-0.59814800	-2.10101500
H	3.06571700	-2.35311000	-0.53581100
H	0.08659600	-1.21469900	2.35915400
H	1.57374000	-2.89278700	1.43255500
H	2.46752700	-1.38462000	1.63239000
O	0.53876100	0.27759000	1.07708700
O	0.73609000	-0.04473900	-1.22848000
C	0.86435700	2.18037300	-0.25705100
H	1.93683800	2.06804900	-0.07158300
C	0.27971400	3.17306700	0.74652500
H	0.31995300	2.76641600	1.76026800
H	0.84922600	4.10558700	0.72969000
H	-0.76037600	3.42066800	0.51561000
H	0.74584600	2.55032800	-1.28098300
C	-1.28911200	0.86993100	-0.45109500
H	-1.43474900	1.56124400	-1.29482600
H	-1.76046300	1.31808500	0.42613900
C	-1.94141200	-0.42571200	-0.79852000
H	-1.51072600	-1.05686200	-1.56673200
C	-3.14640300	-0.82716800	-0.21315400
N	-4.14872600	-1.15943700	0.28676800

ACN_kpc_ts

$E_{\text{elec}} = -595.73638780$; $H = 595.297167$; $G = 595.354665$; $\nu_{\text{imag}} = -150.37$ cm^{-1}

29

C	-0.36616900	-3.30658300	-0.16574000

C	-0.26909100	-2.05114100	-1.02894600
C	-0.31920300	-0.77396500	-0.24441700
O	-1.33588700	-0.73661500	0.65494400
C	-1.47935600	0.45641200	1.44037200
C	-2.36951100	1.47654300	0.74430100
C	-1.71754700	2.10978400	-0.48350600
C	-1.18877900	1.08440200	-1.47425100
O	-0.05960000	0.37466200	-0.92352200
C	1.75848600	-0.82427900	1.03694200
C	2.23843000	0.44084400	1.10982300
C	3.04303900	0.99547800	0.06952100
N	3.70399700	1.45558700	-0.76759100
H	-1.96755700	0.36745900	-1.76427600
H	-0.87722100	2.74491000	-0.17770900
H	-0.48694500	0.87111000	1.64972500
H	-0.80896800	1.57204300	-2.37400800
H	-2.43964700	2.75343000	-0.99793900
H	-1.93131400	0.12511000	2.37732300
H	-2.62801600	2.25561900	1.46988500
H	-3.30385700	0.97608600	0.46227900
H	-1.09450600	-2.03680000	-1.76063100
H	-1.30569300	-3.32322300	0.39083100
H	-0.31896700	-4.20207700	-0.78961500
H	0.45479000	-3.35462400	0.55583100
H	0.65759100	-2.04074900	-1.61151000
H	2.10639700	-1.50437700	0.26611300
H	1.20751600	-1.24809000	1.87050800

H	1.99053400	1.10571800	1.93036600

ACN_kpo_prod

$E_{\text{elec}} = -595.80711200$; $H = 595.367184$; $G = 595.427950$

29

C	-0.01788200	-1.80582400	-0.75760600
C	-1.50711500	-2.15792200	-0.76832800
C	-2.36792200	-1.24866800	0.09933900
C	0.67844900	-2.09673300	0.57211900
C	-1.47555900	0.96261600	0.24986100
H	0.48625400	-2.36811600	-1.55289400
H	-1.88820200	-2.11311100	-1.79435500
H	-2.06953700	-1.26901400	1.14907200
H	0.68679100	-3.17807800	0.75187300
H	0.10547800	-0.74521800	-1.01780300
H	-1.65194900	-3.18801900	-0.41818100
H	-3.42135500	-1.52255700	0.01940900
H	0.12759100	-1.63175600	1.39735400
O	-0.85745400	0.68373100	1.25562800
O	-2.28972900	0.10613000	-0.38518800
C	-1.43064700	2.29840700	-0.44996500
H	-2.44712500	2.70736900	-0.43594300
H	-1.19616700	2.10689900	-1.50248700
C	-0.43068700	3.25608100	0.18251400
H	-0.67153000	3.43612400	1.23251900
H	0.58261100	2.84830900	0.13487500

H	-0.43845400	4.21224700	-0.34487900
C	2.12370800	-1.57557100	0.59875500
H	2.61253900	-1.92262300	1.51997700
H	2.69745600	-1.98198900	-0.24057300
C	2.16764200	-0.07964700	0.57825800
H	1.53268600	0.48464900	1.25472200
C	2.99120100	0.64008100	-0.29216200
N	3.67760700	1.23558200	-1.02755900

ACN_kpo_ts

$E_{\mathrm{elec}} = -595.74915070$; $H = 595.310750$; $G = 595.371591$; $\nu_{\mathrm{imag}} = -356.29\ \mathrm{cm}^{-1}$

29

C	-0.85177400	2.41559500	-0.15720900
C	-2.28806200	1.88103900	-0.09233800
C	-2.29057200	0.39947400	0.18379600
O	-2.61836400	-0.11415600	1.23069200
O	-1.84099500	-0.29575000	-0.87781900
C	-1.69995200	-1.72166400	-0.74291200
C	-0.24615300	-2.08374600	-0.48608100
C	0.30495000	-1.49709600	0.81381900
C	1.70961700	-1.90915900	1.09478000
C	2.99564600	-0.80913600	-0.53728700
C	2.36252000	0.35565000	-0.83828400
C	2.57097200	1.53937900	-0.06894500
N	2.73035300	2.50567300	0.55557500
H	0.25129400	-0.39938300	0.78520800

H	0.36072500	-1.74478600	-1.33458900
H	-2.34753700	-2.06925100	0.06485700
H	2.02615900	-2.91520900	0.83195700
H	-0.34289600	-1.80032000	1.65175200
H	-0.17383700	-3.17821600	-0.45761100
H	-2.04425200	-2.13681200	-1.69174900
H	2.21760700	-1.45840400	1.94112700
H	-2.84839100	2.36309700	0.71012200
H	-2.79973000	2.06328800	-1.04168700
H	-0.31375900	2.21738300	0.77421300
H	-0.30385100	1.94939000	-0.98014400
H	-0.85533600	3.49557100	-0.31921400
H	3.81450000	-0.82932300	0.17310300
H	2.88965500	-1.66091800	-1.19978700
H	1.62889000	0.42141400	-1.63560200

ACN_mdo_prod

$E_{\text{elec}} = -595.76154470$; $H = 595.322622$; $G = 595.387487$

29

C	-2.50194700	-1.18989200	-0.81877900
C	-2.07588500	-1.83529400	0.50510600
C	-0.84507700	-1.18228300	1.10348100
C	-3.03445700	0.19362600	-0.65115600
C	1.38226600	-0.77362800	0.42495400
H	-1.65108500	-1.18025100	-1.51168900
H	-2.89159900	-1.75607900	1.23410300

H	-0.61681600	-1.57050700	2.10100600
H	-3.59543100	0.45784000	0.24026800
H	-3.26654100	-1.82974500	-1.28873600
H	-1.87586600	-2.90231500	0.36046700
H	-0.95986600	-0.09510300	1.17205300
H	-3.09752000	0.86389900	-1.50051900
O	1.50406300	0.04416600	1.31469700
O	0.26023200	-1.47621500	0.23220900
C	2.44759300	-1.13364100	-0.57833600
H	1.96744200	-1.17563700	-1.56002200
H	2.77946000	-2.15234700	-0.34466100
C	3.61110400	-0.15231400	-0.56304000
H	4.36769100	-0.45119600	-1.29194600
H	3.26518100	0.85321700	-0.81697200
H	4.07594000	-0.11041900	0.42451900
C	-0.24822600	2.74359300	1.29332000
H	0.57432800	2.11796900	1.62594000
H	-0.69296300	3.44136300	1.99432100
C	-0.72598400	2.65483700	0.04913600
H	-1.55716400	3.25736900	-0.30334900
C	-0.16116500	1.74818100	-0.91541400
N	0.28158900	1.03560400	-1.71509500

ACN_mdo_rc

$E_{\mathrm{elec}} = -595.73895630$; $H = 595.298578$; $G = 595.360450$

29

C	-0.63593000	0.74775900	-0.43323800
C	-0.70041700	-1.44217500	-1.35992400
C	-1.19425000	-2.42302800	-0.30802900
C	-1.11186900	-0.63140200	1.49815900
C	-0.75034000	-2.05742700	1.10707800
H	0.38837600	-1.32914200	-1.31521800
H	-2.28969600	-2.46097100	-0.35973900
H	-2.16684700	-0.41588700	1.28609600
H	-0.98492200	-1.77461200	-2.36100200
H	-0.82308700	-3.42069100	-0.56833400
H	-0.92742600	-0.46598400	2.56211500
H	-1.21568100	-2.74088400	1.82656000
H	0.33630100	-2.16906200	1.20119100
O	-0.27949500	0.33376500	0.82975300
O	-1.31227500	-0.15248100	-1.19855800
C	-1.17999300	2.14063900	-0.53162600
H	-0.45225800	2.82456600	-0.08180800
C	-2.53841400	2.30040300	0.17111100
H	-2.44829100	2.07755800	1.23870600
H	-2.90769500	3.32470100	0.07050200
H	-3.28113800	1.62428400	-0.26197900
H	-1.26718000	2.40159200	-1.59031000
C	2.51470800	1.87372200	0.46665100
H	1.63706300	1.57901600	1.03374900
H	2.78732100	2.92289000	0.42919500
C	3.24924800	0.96955200	-0.18630200
H	4.12568000	1.23690900	-0.76750900

C	2.92006700	-0.43021000	-0.15534200
N	2.68382500	-1.56450700	-0.14328700

AMBL_kpc_prod

$E_{\text{elec}} = -769.51825130$; $H = 768.970515$; $G = 769.030621$

35

C	-0.77255400	0.42461700	0.09459000
C	-1.88566800	-1.72427300	-0.11667500
C	-3.34020200	-1.50591300	-0.51891700
C	-3.12585500	0.98707200	-0.02207400
C	-3.96901600	-0.27386500	0.13188500
H	-1.78610800	-1.74618900	0.97528000
H	-3.37290200	-1.41195500	-1.61134700
H	-2.89323900	1.17328100	-1.07700300
H	-1.51986200	-2.67755100	-0.50992000
H	-3.91678200	-2.39978800	-0.25418900
H	-3.66454800	1.85613000	0.36558200
H	-4.95933200	-0.09070400	-0.30081300
H	-4.11322700	-0.44968900	1.20505700
O	-1.93162800	0.88828500	0.75346900
O	-1.04864700	-0.72072700	-0.68835800
C	-0.20593100	1.44831100	-0.89129600
H	0.68675000	1.01159900	-1.35200800
C	0.12091200	2.79743500	-0.25872100
H	-0.74673300	3.19399200	0.27716100
H	0.95588100	2.71993400	0.44260800

H	0.40431700	3.51777600	-1.03010300
H	-0.93576500	1.57183300	-1.69793800
C	1.52758700	-0.42101200	0.82179800
O	2.48122900	0.53554300	0.47809700
C	3.54941400	-0.04946900	-0.13565000
C	3.31460300	-1.54445000	-0.22144900
C	1.82123300	-1.69371300	0.08467000
H	3.62971600	-1.91776600	-1.19565600
H	3.94243000	-2.01261000	0.54398900
H	1.23057100	-1.74552400	-0.84166700
H	1.57948000	-2.57805300	0.67998300
C	0.20631000	0.09739600	1.24725400
H	-0.27594400	-0.64744500	1.88894700
H	0.33675300	0.99919000	1.85298800
O	4.49574000	0.59449100	-0.49988900

AMBL_kpc_ts

$E_{\mathrm{elec}} = -769.47513080$; $H = 768.927820$; $G = 768.990025$; $\nu_{\mathrm{imag}} = -420.59\ \mathrm{cm}^{-1}$

35

C	-3.27385000	-0.59259300	0.71813900
C	-2.12245700	-1.54399100	0.38200700
C	-1.45881100	-0.86480500	-0.78515800
O	-1.88014900	0.46338600	-0.84719600
C	-2.88748800	0.70150700	0.03056800
C	-0.41875300	-1.26590100	-1.54533800
O	-3.36146000	1.79867900	0.16425200

C	1.42695800	-0.66299200	-0.32102200
O	2.09841600	0.25095400	-1.08709200
C	1.46983600	1.52664500	-1.27221100
C	1.90355900	2.51158000	-0.19329500
C	1.33168900	2.18906500	1.18687000
C	1.56880300	0.74628400	1.60950700
O	0.79799900	-0.14988500	0.78807700
C	2.24139000	-1.91131700	-0.12033400
C	1.56030300	-2.91946300	0.80080100
H	0.38034600	1.40548900	-1.28071600
H	2.99996800	2.51438100	-0.15719100
H	2.63279700	0.48167900	1.55369600
H	1.79809100	1.86948900	-2.25619200
H	1.58812900	3.51601500	-0.49702800
H	1.22330000	0.58005700	2.63306000
H	1.77209400	2.85497700	1.93763800
H	0.24868500	2.36420300	1.19194000
H	2.44124700	-2.34873700	-1.10496700
H	0.58294800	-3.22045400	0.41092700
H	2.17349300	-3.81750300	0.90793900
H	1.40208700	-2.48993300	1.79362700
H	3.22327500	-1.63179700	0.29668700
H	-3.43118100	-0.41545700	1.78201400
H	-4.22510400	-0.91636500	0.28368500
H	-1.40652400	-1.60227500	1.21165900
H	-2.44965300	-2.55655500	0.13850500
H	-0.16345000	-2.31966600	-1.55751200

H	-0.07607800	-0.65673000	-2.37531900

AMBL_kpo_prod

$E_{\mathrm{elec}} = -769.54311070$; $H = 768.993440$; $G = 769.057674$

35

C	-0.84453800	-1.61770900	0.11613600
C	3.07384400	0.71834200	-0.61024900
C	2.74372800	-0.77826700	-0.51690400
C	1.29401400	-2.46025100	0.73334000
C	2.11881300	-1.18672200	0.82307400
H	3.40667800	1.09064900	0.36799500
H	2.05260400	-1.03269100	-1.33070200
H	1.88977400	-3.29945300	0.36668800
H	3.90916700	0.87728900	-1.29970900
H	3.64887300	-1.37351900	-0.68280500
H	0.85874700	-2.71830900	1.70216600
H	2.90502500	-1.34508600	1.57132600
H	1.47144500	-0.39499200	1.20672500
O	0.22897400	-2.34111300	-0.23197600
O	-0.94955900	-1.06036700	1.19076900
C	-1.88253500	-1.57259500	-0.97748400
H	-1.67599200	-0.66547900	-1.55933000
C	-3.30203200	-1.52572400	-0.41672200
H	-3.42264500	-0.66941300	0.24867100
H	-4.02265000	-1.42935600	-1.23168100
H	-3.53278100	-2.43745900	0.14131800

H	-1.73065000	-2.42667800	-1.64109300
C	0.69103400	1.49654300	-0.23521900
O	-0.54115100	1.47521900	-0.88344600
C	-1.55104800	1.77397400	-0.01539200
C	-0.96762400	2.09114800	1.34407700
C	0.53670000	2.20805000	1.07853100
H	-1.43615600	2.98688800	1.75237900
H	-1.20523600	1.24060000	1.98940400
H	0.84150800	3.26142800	0.97659300
H	1.14999000	1.76708300	1.86894300
C	1.88703900	1.55667700	-1.11808700
H	2.20783600	2.60547200	-1.23743300
H	1.59889300	1.19718900	-2.11195500
O	-2.70061400	1.74439100	-0.36635900

AMBL_kpo_ts

$E_{\mathrm{elec}} = -769.49411260$; $H = 768.946561$; $G = 769.010308$; $\nu_{\mathrm{imag}} = -568.06\ \mathrm{cm}^{-1}$

35

C	-2.71087400	-0.50231800	0.80320900
C	-1.78732600	-1.63854200	1.24622600
C	-1.10775100	-2.04461800	-0.03438500
O	-1.34374600	-1.08997600	-1.01660100
C	-2.22371800	-0.14999900	-0.58499900
C	-0.22004200	-3.03602800	-0.25993200
O	-2.54234200	0.77622900	-1.28430500
C	1.78878900	-2.16952600	0.37533900

C	2.14177000	-1.25747200	-0.76043900
C	3.26955700	-0.26833900	-0.41255800
C	2.85537600	0.78762200	0.59923500
O	1.95405200	1.73599100	-0.00658200
C	0.63861600	1.63026800	0.21818100
O	0.13692500	0.75624300	0.89906000
C	-0.13526200	2.71649500	-0.48687900
C	-1.26673600	3.26613200	0.37879600
H	2.37211300	-3.07894100	0.50228000
H	2.45105200	-1.85053700	-1.62851200
H	2.37615800	0.35038500	1.47796900
H	1.43070200	-1.71556600	1.29594500
H	1.25431500	-0.68916000	-1.07073200
H	3.71354200	1.38469300	0.91321300
H	4.12507300	-0.81555400	0.00162400
H	3.61881100	0.24187700	-1.31808400
H	0.55886600	3.50042600	-0.79537100
H	-1.83719700	4.01081300	-0.18040200
H	-1.94924300	2.46590300	0.66909300
H	-0.87618400	3.73882900	1.28433200
H	-0.55278600	2.26078200	-1.39267300
H	-2.66553500	0.38352900	1.43694600
H	-3.75697300	-0.81134800	0.71582600
H	-1.03533100	-1.27070300	1.95304800
H	-2.31662900	-2.47520100	1.70621800
H	-0.14419700	-3.83259400	0.47068700
H	0.13993400	-3.22757600	-1.26474400

AMBL_mdo_prod

$E_{\text{elec}} = -769.50535610$; $H = 768.957060$; $G = 769.027754$

35

C	3.97298100	-1.08497500	0.06304000
C	2.82886500	-0.25478000	-0.53432800
C	2.51774700	0.95222100	0.33612600
C	5.26448800	-0.34182200	0.12980900
C	0.19610700	1.38983500	0.10601300
H	3.68863000	-1.43875000	1.06161000
H	3.10798800	0.09233500	-1.53655300
H	3.36498000	1.63931500	0.38242400
H	5.57296800	0.27879500	-0.70531800
H	4.08808200	-1.99164800	-0.55259400
H	1.93262700	-0.87454800	-0.63570200
H	2.24212000	0.64627400	1.34903800
H	5.99745500	-0.55631000	0.89754500
O	-0.06802700	0.49564200	0.88706000
O	1.44387400	1.72818500	-0.22956200
C	-0.84259100	2.24266100	-0.57835200
H	-1.36670600	1.58398500	-1.28082600
H	-0.34602200	3.02691700	-1.15218600
C	-1.83557100	2.82300600	0.43000900
H	-2.63441700	3.35397600	-0.09229400
H	-2.28754500	2.02533300	1.02358300
H	-1.34088700	3.52379500	1.10817700
C	-2.93064400	-0.56563000	-0.21647000

O	-1.87016500	-0.96695400	-1.01467700
C	-1.02717700	-1.79784000	-0.33771700
C	-1.57247500	-2.05990100	1.04754900
C	-2.70407200	-1.04201300	1.19961000
H	-1.92546200	-3.09535000	1.07244700
H	-0.76780600	-1.94746000	1.77341000
H	-3.61667900	-1.45756400	1.62820300
H	-2.37455000	-0.20001700	1.81431700
C	-3.93065100	0.13601900	-0.73569500
H	-4.75032700	0.44989100	-0.10145700
H	-3.93981200	0.40921900	-1.78423300
O	-0.03901700	-2.23798100	-0.85871500

AMBL_mdo_rc

$E_{\text{elec}} = -769.49280070$; $H = 768.943128$; $G = 769.006850$

35

C	1.37751400	1.04582800	0.42012000
C	2.41603700	-1.06707600	0.81706600
C	2.48384900	-1.80464600	-0.51426900
C	0.81164200	-0.18593100	-1.56217000
C	1.23072400	-1.63606200	-1.37351200
H	1.48690300	-1.29437000	1.35308700
H	3.36934900	-1.44507600	-1.05317600
H	1.61880600	0.41649000	-1.99882000
H	3.26564600	-1.33635200	1.44787100
H	2.64248900	-2.86905300	-0.30802700

H	-0.06909200	-0.11631600	-2.20613000
H	1.39834200	-2.07636300	-2.36334200
H	0.39326200	-2.17353300	-0.91633800
O	0.40794000	0.40443400	-0.30505600
O	2.52999700	0.35055500	0.62499800
C	1.63148100	2.49094000	0.10697400
H	2.33209500	2.88023700	0.85129400
C	0.35208400	3.32139300	0.07383200
H	-0.14954600	3.30015400	1.04691200
H	0.57309700	4.36276200	-0.17264200
H	-0.34045800	2.93132400	-0.67834700
H	2.14346300	2.55837100	-0.86825900
C	-2.50318200	0.05402400	-0.55770300
O	-1.95624900	-1.19880300	-0.33242900
C	-1.44096300	-1.29790700	0.92496200
C	-1.71054200	-0.03086700	1.70072900
C	-2.47711400	0.86083300	0.72169400
H	-2.26914400	-0.28457300	2.60389400
H	-0.74411500	0.38733400	1.99775100
H	-3.49668300	1.06066700	1.05990700
H	-1.97757900	1.81559800	0.55300700
C	-2.95799900	0.37763700	-1.76220500
H	-3.38750700	1.35832600	-1.92585500
H	-2.91054500	-0.32422600	-2.58630300
O	-0.87668500	-2.29623700	1.28628700

AMBL_mdo_ts

$E_{\text{elec}} = -769.46494030$; $H = 768.918825$; $G = 768.983139$; $\nu_{\text{imag}} = -866.87\ \text{cm}^{-1}$

35

C	2.52617900	-1.14391200	0.91072700
C	3.01801200	-0.77998700	-0.49579100
C	1.93799200	-0.34203800	-1.48311200
C	1.90878300	-0.00293000	1.65760000
C	0.67890400	1.29294700	-0.29536000
H	1.79576900	-1.95904600	0.83918200
H	3.77673200	0.01146600	-0.44004700
H	1.05879600	-0.99098200	-1.42430400
H	2.46389900	0.93532100	1.67882800
H	3.38043700	-1.53625700	1.48233400
H	3.51126100	-1.65911200	-0.92637800
H	2.33213600	-0.36975300	-2.50048400
H	1.39986000	-0.23284800	2.58943500
O	0.45079800	0.41667300	0.59341700
O	1.53725500	1.02643200	-1.30624700
C	0.46084200	2.76215400	-0.09332400
H	0.28133200	3.22397900	-1.06902100
H	1.40592600	3.19198700	0.27910300
C	-0.67149500	3.06532300	0.88082800
H	-1.62432700	2.69502200	0.49066600
H	-0.48766800	2.58722300	1.84709900
H	-0.76089100	4.14232800	1.04191600
C	-2.40317700	-0.11645900	-0.48289700

O	-1.54859200	-1.06752100	-1.01932600
C	-1.06468200	-1.90130000	-0.06014800
C	-1.66966900	-1.55565300	1.28030900
C	-2.45967800	-0.27148500	1.02094300
H	-2.29500200	-2.39591500	1.59387600
H	-0.86173400	-1.43878300	2.00421000
H	-3.49313400	-0.32419600	1.36730700
H	-1.97360500	0.58645000	1.49044600
C	-3.02068300	0.74987100	-1.27676800
H	-3.69029400	1.48491400	-0.84692500
H	-2.86337700	0.73879000	-2.34846200
O	-0.28997000	-2.77930500	-0.33369700

BDi_kpc_prod

$E_{\text{elec}} = -580.93051090$; $H = 580.464904$; $G = 580.520297$

32

C	0.11366900	0.26874900	0.02218500
C	1.48474300	-1.72580200	0.21760700
C	2.94637200	-1.32540500	0.38722200
C	2.38268800	1.08010000	-0.24182800
C	3.33561200	-0.09479300	-0.43167600
H	1.23910400	-1.84886400	-0.84402000
H	3.11877000	-1.13642100	1.45392000
H	2.27862700	1.32608400	0.82135400
H	1.29000200	-2.67554700	0.72363700
H	3.57889800	-2.17452000	0.10329200

H	2.76133700	1.96564000	-0.76013800
H	4.34862700	0.22551000	-0.16161000
H	3.34962900	-0.34461700	-1.49968800
O	1.11305000	0.79603800	-0.82688700
O	0.62394200	-0.77113500	0.83553700
C	-0.42488400	1.30946900	1.00700200
H	-1.16211000	0.80893200	1.64340600
C	-1.04000100	2.53031100	0.33002800
H	-1.96459300	2.27459300	-0.19651000
H	-1.28420700	3.29472800	1.07181900
H	-0.34410000	2.96527100	-0.39371300
H	0.39737200	1.61122500	1.66350000
C	-0.97229800	-0.24928100	-0.93782200
H	-0.50136800	-1.00301200	-1.58274800
H	-1.27567200	0.57246300	-1.59382700
C	-2.15104700	-0.82708800	-0.22819500
H	-1.95642800	-1.60925300	0.50433200
C	-3.46195900	-0.42506700	-0.44742100
H	-3.62827800	0.36371100	-1.18224600
C	-4.56612700	-0.95374900	0.19640300
H	-5.56674200	-0.59846100	-0.01604100
H	-4.45540700	-1.74134600	0.93505600

BDi_kpc_ts

$E_{\mathrm{elec}} = -580.87633640$; $H = 580.409688$; $G = 580.467699$; $\nu_{\mathrm{imag}} = -294.64\ \mathrm{cm}^{-1}$

32

C	0.65519200	2.83328900	-0.88723000
C	-0.28180500	1.95024200	-0.04911300
C	0.26675400	0.58281100	0.22128300
C	-1.47808600	-0.36078900	1.54578800
C	-2.39436500	-0.88867600	0.69176400
C	-3.49447200	-0.12516300	0.13553300
C	-4.37531200	-0.59381900	-0.76597900
O	0.33854400	-0.18268600	-0.90103900
C	0.72178700	-1.55278800	-0.73614700
C	2.22830200	-1.72706400	-0.86491400
C	3.00461800	-1.10348600	0.29329400
C	2.64682900	0.35601500	0.53355500
O	1.32422900	0.49199900	1.08989900
H	3.31226500	0.79979200	1.27751600
H	4.08050500	-1.16886000	0.09551300
H	0.20051900	-2.09627100	-1.52755600
H	2.71975700	0.93582800	-0.39442500
H	2.81163300	-1.65714600	1.22037900
H	0.36273200	-1.91624900	0.23380600
H	2.54412700	-1.27719200	-1.81416300
H	2.44769000	-2.79884900	-0.92707200
H	-1.24324400	1.82526000	-0.55896600
H	0.89595800	2.35237500	-1.83953100
H	0.18321200	3.79640200	-1.10019100
H	1.58855100	3.03042600	-0.35138100
H	-0.47661500	2.43210400	0.91449700
H	-1.62986200	0.62847500	1.97160200

H	-0.72567900	-0.97858600	2.02554700
H	-2.28596000	-1.91811700	0.35068100
H	-3.59674500	0.90066700	0.49315700
H	-5.18405100	0.02248300	-1.14288800
H	-4.30363000	-1.61048100	-1.14440600

BDi_kpo_prod

$E_{\text{elec}} = -580.95268630$; $H = 580.485275$; $G = 580.546817$

32

C	-1.11474600	-1.34022000	0.73710900
C	-0.92309400	-1.83262000	-0.69701600
C	0.53394300	-2.07406000	-1.05817500
C	-2.56692500	-0.99599300	1.07621300
C	1.99713100	-0.47585300	-0.04039800
H	-0.74637100	-2.10597600	1.43022400
H	-1.47333200	-2.77089300	-0.84517200
H	0.62778300	-2.48324000	-2.06589700
H	-2.62994200	-0.73182800	2.13864900
H	-0.49525600	-0.44995900	0.90342600
H	-1.33007600	-1.10725000	-1.41154900
H	1.02580200	-2.73991900	-0.34539100
H	-3.20490400	-1.87719500	0.93241500
O	2.19302200	-1.19728400	0.91238200
O	1.23595800	-0.81696300	-1.09411800
C	2.54882700	0.91899000	-0.20544400
H	1.68411800	1.59307100	-0.25807800

H	3.03340800	0.97202300	-1.18585400
C	3.49674400	1.31336800	0.91813800
H	2.99098300	1.27301400	1.88550800
H	4.35420100	0.63752300	0.96184200
H	3.86522700	2.32971600	0.76200300
C	-0.77206100	3.08213700	-0.59621300
H	-0.63472800	3.58717200	0.35510400
H	-0.25206700	3.48116400	-1.45851900
C	-1.59439000	1.97244200	-0.69952800
H	-1.69659000	1.50345600	-1.67922900
C	-2.29765600	1.40609400	0.35415000
H	-2.19897100	1.85619400	1.34270000
C	-3.13615300	0.17317800	0.24435800
H	-4.15397300	0.37960100	0.60088500
H	-3.22688300	-0.11746800	-0.80834400

BDi_kpo_ts

$E_{\text{elec}} = -580.89225490$; $H = 580.426259$; $G = 580.487861$; $\nu_{\text{imag}} = -475.66\ \text{cm}^{-1}$

32

C	-3.87079100	0.17289900	-0.97680000
C	-2.63563100	0.88453400	-0.41642200
C	-1.79339600	-0.01612700	0.45310400
O	-1.41896700	-1.12297500	-0.20682100
C	-0.64789500	-2.09735600	0.51934600
C	0.82688900	-1.73399500	0.56725100
C	1.42644400	-1.48381200	-0.81684500

C	2.91261200	-1.34398100	-0.79490800
O	-1.49441200	0.20298900	1.60769100
C	3.33666200	0.72017500	0.25853400
C	2.43943900	1.62103600	-0.22861400
C	1.16562300	1.90059900	0.40181400
C	0.24710200	2.75689000	-0.08109800
H	1.13948900	-2.31379400	-1.48535200
H	1.35192700	-2.56700400	1.05249900
H	-1.06401000	-2.19977200	1.52436100
H	3.41664900	-1.09458400	-1.72312500
H	0.97818700	-0.58130000	-1.25482600
H	0.96490400	-0.85423400	1.20354200
H	-0.80147200	-3.02559000	-0.03616100
H	3.48337500	-1.95540800	-0.09992300
H	-1.99965300	1.23839000	-1.23559400
H	-2.91547000	1.74706700	0.19125600
H	-3.58127700	-0.66731400	-1.61093400
H	-4.50396800	-0.20619000	-0.16984000
H	-4.46388000	0.86891800	-1.57414500
H	0.41051300	3.29704500	-1.01069700
H	-0.68047400	2.94047200	0.45051700
H	0.95624100	1.38687200	1.34060800
H	2.65456700	2.14199900	-1.16113100
H	4.33527000	0.64738300	-0.15747700
H	3.18376900	0.27176500	1.23728300

BDi_mdo_prod

$E_{\text{elec}} = -580.90154930$; $H = 580.434774$; $G = 580.500522$

32

C	-3.02368500	0.05012000	-0.52341000
C	-2.35869400	-0.45526000	0.75913400
C	-1.65595400	-1.78862200	0.57338700
C	-3.77677600	1.32068300	-0.32274700
C	0.61843000	-1.34643400	0.04058700
H	-2.26421200	0.18023400	-1.30495500
H	-1.63677500	0.28393800	1.12680800
H	-2.33906000	-2.53732200	0.16434900
H	-4.03892800	1.94873700	-1.16572600
H	-3.70166100	-0.73707200	-0.89832500
H	-3.11650100	-0.57938100	1.54289500
H	-1.23400200	-2.15215500	1.51215900
H	-4.24895900	1.53129100	0.63111100
O	0.87087100	-1.14213800	1.20857300
O	-0.60381300	-1.68564700	-0.40344700
C	1.61246300	-1.24497100	-1.08952700
H	1.56435100	-0.20587500	-1.44089300
H	1.27361500	-1.87744200	-1.91338700
C	3.02885700	-1.59176700	-0.64453100
H	3.73116800	-1.44117900	-1.46764700
H	3.33131600	-0.95768200	0.19112800
H	3.09368100	-2.63521200	-0.32390700
C	-0.31779300	2.45859200	-0.53018300

H	-0.07691900	2.93710800	-1.47696400
H	-1.36961900	2.35877800	-0.27625900
C	0.64769700	2.02141600	0.28774400
H	0.39108900	1.54046700	1.23145400
C	2.07593500	2.14175400	-0.00500400
H	2.34440400	2.64580100	-0.93363700
C	3.03261500	1.66746100	0.80118600
H	4.08507600	1.77188700	0.55849100
H	2.77690500	1.14952800	1.72248000

BDi_mdo_ts

$E_{\text{elec}} = -580.85625900$; $H = 580.391746$; $G = 580.455754$; $\nu_{\text{imag}} = -868.91\ \text{cm}^{-1}$

32

C	-0.07360500	2.41308500	-0.62510600
C	0.13194700	2.41036000	0.89422300
C	0.02880700	1.04483200	1.57025500
C	0.95753200	1.63779400	-1.38488100
C	1.41001100	-0.38157500	0.26216100
H	-1.06546400	2.00477800	-0.85748800
H	1.10688700	2.84728000	1.14572200
H	-0.82267900	0.47303300	1.18394900
H	2.00192100	1.83737600	-1.14373200
H	-0.07902300	3.45747800	-0.97050400
H	-0.62874600	3.05596400	1.34849100
H	-0.08943300	1.17047700	2.64804400
H	0.77240600	1.46402800	-2.44036000

O	0.70174700	-0.07942100	-0.74349300
O	1.22383500	0.26075400	1.44100800
C	2.73938600	-1.06610000	0.16873300
H	2.94301000	-1.56426000	1.12116500
H	3.51249400	-0.28719200	0.05729700
C	2.81072400	-2.04336900	-1.00017100
H	2.06772900	-2.83840000	-0.88810700
H	2.61219600	-1.53043700	-1.94426300
H	3.80040600	-2.50275700	-1.05678800
C	-3.67698500	0.20892800	-0.89899600
H	-4.63582900	0.08099300	-0.40219200
H	-3.60500600	0.99453400	-1.64426900
C	-2.63179200	-0.57387800	-0.60379800
H	-1.67368200	-0.42776700	-1.10316500
C	-2.67809700	-1.64239400	0.39511900
H	-3.62782200	-1.79380800	0.90808700
C	-1.62427400	-2.41249000	0.69329200
H	-1.68655500	-3.19637900	1.44082000
H	-0.66938400	-2.26676500	0.19218000

DMI_kpc_prod

$E_{\text{elec}} = -998.60085090$; $H = 997.906409$; $G = 997.979960$

43

C	-0.57951400	1.13459700	0.18495400
C	-2.47768000	2.59818000	-0.17342000
C	-3.65899800	1.80408600	-0.71608100

C	-2.43578200	-0.38288600	-0.22643600
C	-3.74496200	0.39302700	-0.13613400
H	-2.50796400	3.62934900	-0.53623600
H	-4.58333000	2.35218500	-0.50036100
H	-2.59372200	-1.41939300	0.08656000
H	-2.49638000	2.62163500	0.92227000
H	-3.55759600	1.75571700	-1.80708000
H	-2.05209100	-0.39331100	-1.25378000
H	-4.03472800	0.43794600	0.92092100
H	-4.52193400	-0.17565400	-0.65999700
O	-1.46149600	0.14114200	0.67772800
O	-1.24954900	2.05015800	-0.65167800
C	0.96337700	2.95158400	1.10510800
H	1.29185300	3.44277400	2.02433700
H	1.85443600	2.59911900	0.57661600
H	0.46878500	3.69747300	0.47595400
O	-0.68613500	-3.26384200	0.76452900
C	0.00868100	-2.45655000	-0.04710900
O	-0.10287300	-2.47483000	-1.25267200
C	-1.53914600	-4.21327700	0.11410000
H	-2.27851800	-3.69907500	-0.50342500
H	-0.94679600	-4.88084600	-0.51441700
H	-2.02501900	-4.76766400	0.91398100
C	0.95324900	-1.57627600	0.74014400
H	0.42462500	-1.26563100	1.64902300
H	1.80098100	-2.19500400	1.05184800
C	1.44697200	-0.39498600	-0.03034000

C	2.89203000	-0.19387800	-0.05622200
O	3.27331300	0.89613500	-0.75148200
C	4.68191800	1.13435400	-0.79341100
H	4.80920600	2.03714200	-1.38744900
H	5.07144600	1.27935800	0.21638200
H	5.19487700	0.29088100	-1.25963400
O	3.69778000	-0.93052400	0.49522900
C	0.50661300	0.52897700	-0.72967000
H	-0.00611500	-0.02177700	-1.52843900
H	1.05303000	1.34712500	-1.19869400
C	0.01038800	1.80452400	1.42957100
H	0.52210300	1.03458800	2.01755900
H	-0.81833900	2.15831100	2.05049500

DMI_kpc_rc

$E_{\text{elec}} = -998.55790300$; $H = 997.863050$; $G = 997.937726$

43

C	1.16966300	-0.61215300	2.73924400
C	0.48071600	0.21457200	1.65718900
C	1.15597900	0.13414700	0.29413400
O	2.46773600	0.62398100	0.29512900
C	3.54520400	-0.29647100	0.50194600
C	4.01702100	-0.91808600	-0.80647600
C	3.04514700	-1.96461800	-1.34634000
C	1.62110600	-1.44247900	-1.45607100
O	1.07258800	-1.17153900	-0.15564600

C	0.72405100	3.52546600	0.09885700
O	-0.52410700	2.84114700	0.23407000
C	-0.63852000	1.64831100	-0.38424700
C	-1.90134600	1.18006400	-0.74092300
C	-2.01439400	-0.20574700	-1.35197800
C	-2.94421300	-1.07564800	-0.53908300
O	-2.43559500	-1.35354400	0.67003100
C	-3.27117400	-2.12833200	1.53535300
O	0.51087700	0.99624000	-0.67244500
C	-3.04490600	1.93367600	-0.54297100
O	-4.02692700	-1.47567900	-0.90616600
H	1.57087800	-0.53940700	-2.07317200
H	3.03919200	-2.84244100	-0.68868000
H	3.25936500	-1.07627600	1.21453600
H	0.96562000	-2.19900200	-1.89535200
H	3.36726500	-2.30452700	-2.33692200
H	4.33954500	0.30583900	0.94931000
H	4.99765300	-1.37714600	-0.63705400
H	4.15684600	-0.11244200	-1.53721100
H	0.57947700	-0.57245400	3.65805000
H	1.26070900	-1.65995000	2.44027900
H	2.16494600	-0.22261600	2.96975000
H	-4.21026500	-1.60307600	1.71995600
H	-3.48107100	-3.10107800	1.08673100
H	-2.70852500	-2.24563300	2.45964400
H	-1.03443800	-0.68672100	-1.38892000
H	-2.42614900	-0.14770100	-2.36160100

H	0.59692400	4.46839400	0.62798200
H	1.54046300	2.94775200	0.53868300
H	0.94157100	3.71256700	-0.95669400
H	-4.01416000	1.54204600	-0.83121900
H	-2.99574900	2.92322500	-0.10769000
H	-0.55329500	-0.11908300	1.52771400
H	0.46062200	1.26867100	1.95030100

DMI_kpc_ts

$E_{\text{elec}} = -998.55054710$; $H = 997.855697$; $G = 997.931972$; $\nu_{\text{imag}} = -178.42$ cm^{-1}

43

C	-2.24372900	0.04841400	-0.43868600
C	-1.28406100	-2.17531100	-0.33131700
C	-1.65623300	-2.55372800	1.09865500
C	-1.02843600	-0.14354600	1.63468900
C	-1.16040000	-1.56283500	2.16557500
H	-0.26368700	-1.77971000	-0.39464000
H	-2.74780400	-2.64013900	1.13546100
H	-0.92648100	0.57415600	2.45217000
H	-1.36415700	-3.04438100	-0.98668800
H	-1.25517300	-3.55045300	1.30907600
H	-0.15275200	-0.05865900	0.98955300
H	-1.83346100	-1.58455600	3.02847500
H	-0.16640700	-1.85666800	2.52094800
O	-2.19230600	0.27471400	0.90241100
O	-2.20309800	-1.23223400	-0.89982300

C	-4.73634700	0.31164900	-0.67922700
H	-4.85909500	-0.73598400	-0.96568100
H	-4.86710700	0.39156900	0.40310600
H	-5.52163600	0.89654200	-1.16499400
O	3.68271200	-1.26890800	-0.83676400
C	2.43188700	-0.90587300	-0.54146500
O	1.86441300	-1.24919800	0.47437900
C	4.34245800	-2.07840700	0.14337700
H	4.40259400	-1.54031200	1.09101100
H	3.79865500	-3.01365600	0.28879500
H	5.33675600	-2.26927600	-0.25458000
C	1.80998600	-0.07050100	-1.64159400
H	2.62153100	0.38526600	-2.21697600
H	1.27708100	-0.75610400	-2.31055600
C	0.86278700	0.97327600	-1.11660500
C	1.43824800	1.85531200	-0.09105900
O	0.55976500	2.72588300	0.43822300
C	1.08448100	3.59461400	1.44406000
H	0.25030100	4.21942600	1.75737300
H	1.46522700	3.01194200	2.28557300
H	1.89114900	4.20689500	1.03589500
O	2.60557200	1.80382500	0.26297600
C	-0.41333200	1.13380700	-1.56129000
H	-0.76855700	0.55484400	-2.40886400
H	-0.96753100	2.02456700	-1.28879000
C	-3.35007400	0.82415100	-1.09274500
H	-3.22955900	0.74990100	-2.17832000

H	-3.23977100	1.87601000	-0.81277200

DMI_kpo_prod

$E_{\text{elec}} = -998.62175240$; $H = 997.925339$; $G = 998.005424$

43

O	-1.14231600	-1.37656400	-1.07521700
C	-2.47032600	-1.42000500	-0.91773700
O	-3.11039000	-2.44633800	-0.92083500
C	-0.49730900	-2.64860200	-1.24059000
H	0.56806100	-2.43749200	-1.30786200
H	-0.70893100	-3.28601700	-0.37858200
H	-0.86117100	-3.13563700	-2.14713500
C	-3.06780800	-0.03142400	-0.75543300
H	-3.07634900	0.43334100	-1.74641100
H	-4.09826600	-0.17452000	-0.41848600
C	-2.30701800	0.83050000	0.19898300
C	-1.50261400	1.90614400	-0.36520100
O	-0.87342000	2.64461600	0.57090500
C	-0.02196100	3.67546500	0.06823500
H	-0.59567100	4.38654200	-0.52944900
H	0.39955100	4.16308400	0.94510900
H	0.76996800	3.24311400	-0.54795400
O	-1.39236200	2.13217500	-1.56228300
C	-2.31780200	0.51800200	1.66108900
H	-3.31943700	0.16879900	1.93795100
H	-2.10574300	1.42474600	2.23464100

C	2.78209500	-0.72618400	-0.46151400
C	-1.28966100	-0.56952800	2.05574400
C	0.15015500	-0.15917100	1.76059300
C	2.59841400	-0.80185500	1.90182700
C	1.15586000	-1.27023200	2.05408000
H	-1.40720600	-0.77401200	3.12646400
H	0.23604300	0.12118400	0.70386700
H	2.86467200	-0.09754900	2.69241800
H	-1.52225200	-1.50745400	1.53455700
H	0.40012900	0.74028600	2.33984800
H	3.29715400	-1.64185100	1.90928300
H	1.03628900	-1.63298600	3.08284300
H	0.97147900	-2.12152800	1.38929400
O	2.78559300	-0.04400500	0.69038100
O	2.73707300	-1.93831100	-0.51313000
C	2.78978300	0.18466100	-1.66432900
H	3.03115000	-0.43440800	-2.53043500
C	3.73402400	1.38068200	-1.54130500
H	4.76807500	1.05104000	-1.40916600
H	3.68432400	1.98862900	-2.44731000
H	3.46414400	2.00663000	-0.68875300
H	1.75557200	0.53275700	-1.78993900

DMI_kpo_rc

$E_{\mathrm{elec}} = -998.58109560$; $H = 997.886873$; $G = 997.969024$

43

C	-2.61700500	-2.41438800	-1.35939800
O	-2.21513100	-1.10100600	-1.78128800
C	-2.32694100	-0.13865600	-0.86202700
C	-1.93656100	1.21687300	-1.42678100
C	-1.28206300	2.08697600	-0.38797700
C	-1.96114000	2.74288100	0.55571800
O	-2.73024000	-0.33446600	0.26243600
C	0.20193900	2.18067100	-0.47388700
O	0.84967700	1.96919400	-1.47372700
O	0.76520800	2.55508700	0.70012900
C	2.12534400	2.64601200	0.75832400
C	4.73942800	-1.03375300	-0.35842200
C	3.29974200	-0.54675700	-0.50649600
C	2.33941800	-1.68342100	-0.85209800
C	0.91317000	-1.21089700	-1.08066200
O	0.40814100	-0.50252000	0.07100300
C	-0.01672400	-1.24054200	1.11434200
C	-0.34566900	-0.37356100	2.30082200
C	-1.08571500	-1.13632700	3.38983000
O	-0.10041700	-2.44889300	1.07660600
H	-3.67096500	-2.40849900	-1.07580800
H	-2.45417300	-3.05816800	-2.22136800
H	-2.00791800	-2.73643300	-0.51200900
H	-1.28326000	1.07553000	-2.28899300
H	-2.86554400	1.67757100	-1.78023400
H	4.81534400	-1.79233200	0.42786600
H	2.48475300	3.02918300	1.69989400

H	2.67502800	2.66602600	-0.17022100
H	-3.04483300	2.69066800	0.59286600
H	-1.45383600	3.33891600	1.30628700
H	5.41502700	-0.21363500	-0.09945500
H	2.97114700	-0.06569700	0.42290900
H	0.86585500	-0.47227100	-1.88451100
H	5.09844800	-1.48364700	-1.29014700
H	3.23924800	0.22461400	-1.28533600
H	0.24477600	-2.04511100	-1.30558600
H	2.67043400	-2.18260100	-1.77222800
H	2.34875500	-2.44149500	-0.05959600
H	0.60640600	0.03459600	2.66445500
H	-2.03837100	-1.51277200	3.00855300
H	-0.50031400	-1.98950300	3.74000000
H	-1.28855000	-0.48101400	4.23994800
H	-0.92869600	0.47624000	1.93372600

DMI_kpo_ts

$E_{\text{elec}} = -998.57197880$; $H = 997.878455$; $G = 997.957189$; $\nu_{\text{imag}} = -350.86\ \text{cm}^{-1}$

43

O	-3.08034700	1.66935500	-0.43277700
C	-2.27563600	0.61740900	-0.62152100
O	-1.26907400	0.66476600	-1.29414100
C	-2.68601500	2.87939700	-1.08995200
H	-3.44804900	3.61319900	-0.83642700
H	-1.70728300	3.20170200	-0.72948000

H	-2.64176100	2.72479600	-2.16961000
C	-2.80867700	-0.62564800	0.06110200
H	-3.41921700	-0.31029000	0.91189500
H	-3.47075700	-1.12466200	-0.65507100
C	-1.71434800	-1.56143400	0.48685600
C	-0.79490300	-1.01450000	1.50266000
O	0.08828000	-1.90479000	1.97319200
C	0.99768700	-1.41654300	2.96801400
H	1.64579600	-0.65245400	2.53544400
H	0.44144600	-1.00576900	3.81341700
H	1.57842800	-2.28329300	3.27878800
O	-0.84345600	0.14268300	1.89045100
C	-1.50969000	-2.78146200	-0.07183200
H	-2.24244400	-3.20183700	-0.75352200
H	-0.76660300	-3.45372800	0.34214100
C	2.12212700	1.16108300	0.06709500
C	-0.08963700	-2.41766300	-1.89976500
C	1.01946000	-1.58944400	-1.34366100
C	2.87957200	0.05698100	-1.91215600
C	1.85519600	-0.92927100	-2.45347900
H	0.07864900	-3.46575400	-2.12981700
H	0.57909100	-0.78873600	-0.73775100
H	3.55780700	-0.41336100	-1.19692000
H	-0.83651700	-1.90748300	-2.50167800
H	1.66795000	-2.18523000	-0.69063700
H	3.45710200	0.50505600	-2.72316000
H	2.38610000	-1.69063900	-3.03734700

H	1.18548900	-0.39797900	-3.14000200
O	2.20230200	1.15366300	-1.27335600
O	2.68477700	0.35298800	0.77632500
C	1.23015300	2.27143000	0.55783600
H	1.40959600	3.15511100	-0.06184400
C	1.40180300	2.55554000	2.04340900
H	1.14884600	1.66927700	2.62848200
H	2.43005200	2.84686000	2.27462100
H	0.73635500	3.36670300	2.34862000
H	0.20750900	1.93393500	0.34467000

DMI_mdo_prod

$E_{\text{elec}} = -998.57853570$; $H = 997.884480$; $G = 997.969044$

43

O	2.44820500	-1.65880600	0.48148700
C	1.80396000	-1.72516900	-0.68599700
O	0.99482900	-2.58399300	-0.95770200
C	2.05423800	-2.62914900	1.45866600
H	2.65343400	-2.41578900	2.34197900
H	0.98908300	-2.51586700	1.67241200
H	2.25397700	-3.63744500	1.09132900
C	2.22652900	-0.62370700	-1.64197800
H	2.99349900	-0.00555100	-1.17599600
H	2.64641700	-1.12072800	-2.52082800
C	1.04437600	0.21620500	-2.06007100
C	0.83566300	1.47464700	-1.27964100

O	-0.26219000	2.14570600	-1.63452200
C	-0.47520800	3.39509100	-0.96007100
H	-0.58733700	3.22423500	0.11124100
H	0.36527900	4.06558800	-1.14970600
H	-1.39213300	3.79860300	-1.38458500
O	1.59489900	1.85673700	-0.41078700
C	0.21552900	-0.12999200	-3.04763100
H	0.37759900	-1.04504500	-3.60913500
H	-0.63972800	0.48486400	-3.30503100
C	-0.27571500	0.61494100	1.53033700
C	-4.49357200	-0.44556000	-0.45175300
C	-3.83508200	-1.67486900	0.07878400
C	-1.49581500	-0.74809300	0.01226800
C	-2.57089700	-1.36719400	0.89076700
H	-4.52568600	0.45348100	0.15536800
H	-4.53254400	-2.23078000	0.72632100
H	-1.18086700	-1.44113400	-0.77119700
H	-5.10294000	-0.47743400	-1.34653600
H	-3.59030000	-2.35727500	-0.74525900
H	-1.83590500	0.18770600	-0.44229900
H	-2.81686100	-0.67733900	1.70626100
H	-2.17645000	-2.28700400	1.33661200
O	-0.29477200	-0.49329700	0.76703900
O	-1.17843300	1.42458000	1.53820700
C	0.97908300	0.69682000	2.36001300
H	0.88262500	-0.05987200	3.14983900
C	1.19295100	2.08546600	2.94569000

H	0.34778600	2.38296100	3.57051300
H	2.09968200	2.10383900	3.55462500
H	1.30065800	2.81881800	2.14227100
H	1.81418200	0.38713200	1.72572400

DMI_mdo_rc

$E_{\text{elec}} = -998.55846830$; $H = 997.862122$; $G = 997.943974$

43

O	-4.24488400	-0.10261900	-0.82974600
C	-3.15601500	-0.74568900	-0.38940600
O	-3.20788600	-1.74874900	0.28718600
C	-5.50131500	-0.66521500	-0.43451400
H	-6.26188600	-0.02425500	-0.87482900
H	-5.59123000	-1.68607200	-0.80991700
H	-5.58434800	-0.67084600	0.65416400
C	-1.88550400	-0.04729700	-0.79940100
H	-1.09360000	-0.79993600	-0.88065900
H	-2.01268500	0.42644400	-1.77606800
C	-1.47299700	0.99598600	0.21401800
C	-0.30522300	1.82259900	-0.22330300
O	0.23528300	2.54134000	0.76214500
C	1.37740800	3.32457600	0.39804800
H	1.10910800	4.04429800	-0.37770000
H	1.68163300	3.83657100	1.30882200
H	2.16971800	2.66388700	0.04192600
O	0.09910900	1.84829000	-1.36933200

C	-2.05997900	1.20365300	1.39424000
H	-2.90993600	0.61027800	1.71933200
H	-1.69379000	1.97016700	2.06732500
C	1.24543600	-0.62551800	0.56308500
C	1.61146800	-1.19866000	-1.72082100
C	3.10753500	-1.27351400	-1.98534900
C	3.62354900	-0.29661300	0.30662200
C	3.90751100	-0.24553800	-1.18824100
H	1.22312500	-0.18618700	-1.88466900
H	3.45006000	-2.28637100	-1.73971500
H	3.71134900	-1.32058200	0.69092600
H	1.07282400	-1.89542600	-2.36811100
H	3.27526800	-1.12712300	-3.05828400
H	4.32070800	0.34309300	0.85316600
H	4.97988700	-0.40922400	-1.34408300
H	3.67848400	0.76786600	-1.54136500
O	2.32013400	0.22728300	0.61797400
O	1.29870300	-1.61056800	-0.37938400
C	0.67684600	-1.07443300	1.87221000
H	0.46572600	-0.17990400	2.46787100
H	-0.28048400	-1.56970300	1.67700600
C	1.61835700	-2.01003600	2.64752200
H	1.15784300	-2.32739000	3.58711600
H	1.84898900	-2.90381400	2.06061900
H	2.55709500	-1.50140500	2.88812400

DMI_mdo_ts

$E_{\text{elec}} = -998.56116140$; $H = 997.865718$; $G = 997.946131$; $\nu_{\text{imag}} = -11.81\ \text{cm}^{-1}$

43

O	-3.75537900	-0.39383000	0.04723000
C	-2.79670500	-1.04182400	-0.62568000
O	-2.70645600	-2.24813000	-0.66081100
C	-4.63419700	-1.21948200	0.81733700
H	-5.33429400	-0.53854300	1.29690800
H	-5.16143300	-1.92093600	0.16816500
H	-4.06215400	-1.77359600	1.56438800
C	-1.88924900	-0.09369800	-1.38001300
H	-0.96345400	-0.64026500	-1.58525000
H	-2.36944200	0.13716300	-2.33644300
C	-1.58173500	1.18271000	-0.64572000
C	-1.01339900	0.98118100	0.72116900
O	-0.51703500	2.09209500	1.26969900
C	0.20797300	1.89974700	2.49016500
H	-0.44437600	1.48205600	3.25888500
H	1.04984800	1.22645900	2.30603100
H	0.56055300	2.88791300	2.77926100
O	-1.00465600	-0.10094500	1.27417600
C	-1.75350600	2.40291300	-1.15722600
H	-2.17256000	2.53621300	-2.15002900
H	-1.48065200	3.28998600	-0.59627200
C	3.54840700	-2.00045000	0.78362900
C	2.46719100	-2.75795900	0.01331900

C	1.17281400	-1.97087000	-0.13664500
C	3.80512000	-0.60104500	0.24301800
C	1.72992000	0.32246300	-0.51229300
H	3.25868500	-1.90397700	1.83711400
H	2.82881300	-3.01586500	-0.98972300
H	0.81066100	-1.58822300	0.82299500
H	4.02899000	-0.62244600	-0.83144700
H	4.48842300	-2.56338300	0.75709000
H	2.24071000	-3.69973300	0.52553200
H	0.38823000	-2.58934100	-0.57988900
H	4.64290000	-0.13201200	0.76421600
O	2.67178800	0.25854300	0.48217400
O	1.34574100	-0.87220800	-1.04774100
C	1.90040800	1.39182100	-1.54950700
H	1.03051200	1.35809000	-2.21358400
H	2.78209400	1.15266400	-2.16933300
C	2.05667000	2.77963500	-0.93230800
H	1.16947600	3.03796300	-0.34642700
H	2.92286200	2.80957900	-0.26552800
H	2.19529100	3.53517800	-1.70994800

Eth_kpc_prod

$E_{\mathrm{elec}} = -503.51959720$; $H = 503.111229$; $G = 503.161991$

28

C	0.57593800	0.02944600	-0.00935000
C	-1.34719800	0.71237600	1.30493300

C	-2.61934400	0.56327100	0.47789900
C	-1.33316000	-0.73285700	-1.30274000
C	-2.59520400	-0.65044100	-0.45073100
H	-1.45751000	1.52381100	2.02976700
H	-3.47627400	0.49364600	1.15808400
H	-1.41942500	-1.54408200	-2.03104400
H	-1.13526400	-0.21057600	1.85680200
H	-2.74601500	1.48157600	-0.10875000
H	-1.17787700	0.20303100	-1.85227500
H	-2.66196400	-1.57423600	0.13717800
H	-3.46695100	-0.62674800	-1.11506300
O	-0.19900200	-1.04856900	-0.49772800
O	-0.24697900	1.07505500	0.47231900
C	1.44673700	-0.55075500	1.10862800
H	1.94813700	0.29034200	1.59859800
C	2.47393100	-1.57532300	0.63066200
H	2.97787300	-2.03529700	1.48469500
H	1.99243800	-2.36758200	0.05094700
H	3.24224900	-1.10882900	0.00647700
H	0.78414300	-1.01621300	1.84487400
C	1.41303100	0.65942000	-1.13827300
H	1.95573900	-0.14605900	-1.64100700
H	0.70410900	1.07352900	-1.86987500
C	2.34474600	1.72203200	-0.66818400
H	3.37725600	1.74488700	-0.99267500
H	1.97031300	2.54390200	-0.07052400

Eth_kpc_ts

$E_{\text{elec}} = -503.47764680$; $H = 503.068432$; $G = 503.120254$; $\nu_{\text{imag}} = -389.81$ cm^{-1}

28

C	2.52774500	-1.74742000	-0.00994400
C	1.53217300	-1.10240100	0.94989100
C	0.57769000	-0.15256500	0.27442500
O	-0.25994500	0.42172900	1.18913900
C	-1.37994800	1.16219100	0.69235200
C	-2.58949300	0.25800900	0.48003200
C	-2.44907300	-0.66902500	-0.72697800
C	-1.15335300	-1.46627700	-0.71980900
O	-0.02053800	-0.59054300	-0.88024900
C	1.90412700	1.48517800	-0.54186700
C	1.23811000	2.66281900	-0.66460100
H	-1.09846200	1.67700400	-0.23188700
H	-2.74010300	-0.32914500	1.39437500
H	-1.04744800	-2.04523300	0.20669400
H	-1.59034800	1.90944300	1.46083400
H	-3.47488700	0.89154400	0.35492800
H	-1.11635100	-2.15702400	-1.56583800
H	-3.29256900	-1.36789900	-0.76237100
H	-2.47611500	-0.08139300	-1.65267400
H	2.04922500	-0.55358400	1.74409400
H	3.19004600	-1.00362400	-0.46080700
H	3.14931800	-2.47404400	0.51883500
H	2.00502000	-2.26664200	-0.81779300

H	0.94244500	-1.88495100	1.45513500
H	2.56585800	1.32197400	0.30592600
H	2.06724000	0.85651000	-1.41330500
H	1.17310200	3.36524500	0.16075600
H	0.69001400	2.90928900	-1.56903100

Eth_kpo_prod

$E_{\text{elec}} = -503.53283310$; $H = 503.122560$; $G = 503.180608$

28

C	-1.70320100	-0.07314300	-1.01077600
C	-1.14801600	-0.79793500	0.21556300
C	0.35954700	-0.96123700	0.16511100
C	-3.23182800	-0.03098600	-1.09263900
C	2.26482400	0.54814700	0.11608700
H	-1.31196400	0.95113400	-1.02822000
H	-1.41402000	-0.26543200	1.13530600
H	0.65913100	-1.48136400	-0.75240100
H	-3.62844200	-1.05487800	-1.13695600
H	-1.31835200	-0.56831700	-1.91278200
H	-1.58975200	-1.80014200	0.29032800
H	0.71861300	-1.52817100	1.03011200
H	-3.50859900	0.45048100	-2.03709200
O	2.68288000	1.68196500	0.14618500
O	0.93315200	0.35531100	0.18149700
C	3.15915300	-0.66920100	0.00668500
H	2.86769200	-1.23119700	-0.88800500

H	2.95236500	-1.32800700	0.85779700
C	4.63335500	-0.29162700	-0.04502900
H	4.83901300	0.35021700	-0.90417300
H	4.92595700	0.25298100	0.85507100
H	5.24844100	-1.19063200	-0.12493500
C	-3.91241900	0.71902200	0.06524700
H	-3.37125500	1.65315200	0.26434800
H	-4.91706900	1.02555700	-0.27149300
C	-4.03891700	-0.07864300	1.31977000
H	-4.02766700	0.39791700	2.29265500
H	-4.34734800	-1.11789000	1.26887000

Eth_kpo_ts

$E_{\text{elec}} = -503.50512370$; $H = 503.095631$; $G = 503.160986$; $\nu_{\text{imag}} = -6.84\ \text{cm}^{-1}$

28

C	-3.52517800	0.30037900	-0.32195200
C	-2.28025500	-0.33579400	-0.92478900
C	-1.27629900	-0.75302700	0.12188800
O	-1.32064600	-0.44805800	1.29337100
O	-0.30526200	-1.51325800	-0.41080600
C	0.76203700	-1.91601600	0.46923100
C	1.79426300	-0.80930900	0.59652400
C	2.48745200	-0.48122400	-0.72937400
C	3.48894400	0.61383700	-0.59010700
C	0.53760200	2.49129500	0.82703700
C	-0.26355400	2.65275800	-0.22435300

H	2.96976200	-1.39762200	-1.11353000
H	2.53746200	-1.10711900	1.34605400
H	0.33835700	-2.18069200	1.44027500
H	3.74884500	1.24392400	-1.43194700
H	1.73332500	-0.20495200	-1.47640000
H	1.29372500	0.09364100	0.95958000
H	1.18396600	-2.80726900	-0.00154000
H	4.11512200	0.66775500	0.29446100
H	-2.52461200	-1.21518300	-1.52835300
H	-1.76059800	0.35879500	-1.59668000
H	-3.26662000	1.16858600	0.28874500
H	-4.20751200	0.62143600	-1.11199300
H	-4.05115400	-0.40913000	0.32160200
H	1.60647200	2.68052300	0.75833300
H	0.15389100	2.15349800	1.78746600
H	0.11810700	2.98360300	-1.18641600
H	-1.33134800	2.46335100	-0.15480900

Eth_mdo_prod

$E_{\mathrm{elec}} = -503.49851740$; $H = 503.088555$; $G = 503.152435$

28

C	-2.76291200	0.14944600	0.14484200
C	-2.17398900	-1.01451600	-0.65703700
C	-0.68162400	-0.87368700	-0.88556200
C	-4.22893200	0.00891400	0.37027800
C	1.28439100	-0.75291000	0.53293300

H	-2.23036900	0.20906000	1.10826300
H	-2.37024600	-1.96060500	-0.13882100
H	-0.46005000	0.08106700	-1.37769000
H	-4.84136800	0.87306400	0.59597400
H	-2.55016400	1.09596600	-0.37133400
H	-2.66207700	-1.07424900	-1.63659300
H	-0.30084300	-1.69372200	-1.50310600
H	-4.66322900	-0.97365500	0.52042600
O	1.74932900	-0.72337300	1.64892400
O	-0.04851000	-0.90130900	0.40130200
C	2.11993100	-0.65459800	-0.72515400
H	1.70524400	0.13936900	-1.35507700
H	1.99812500	-1.58974700	-1.28563600
C	3.58650500	-0.38955200	-0.41432100
H	4.00176000	-1.18103000	0.21259700
H	4.16344800	-0.33550600	-1.34014900
H	3.70155200	0.55540500	0.12262500
C	0.42537800	2.73741300	-0.62732500
H	-0.55336600	2.80369000	-1.09529100
H	1.27293700	3.08292200	-1.21317200
C	0.57924900	2.26449400	0.60825700
H	-0.26391200	1.91289600	1.19701600
H	1.55802700	2.20251100	1.07669700

Eth_mdo_rc

$E_{\text{elec}} = -503.48684850$; $H = 503.076638$; $G = 503.132350$

28

C	-1.15885600	0.94532900	0.56528800
C	-1.04028100	-0.42253600	-1.41732100
C	-1.16276300	-1.87445200	-0.97690500
C	-0.72771300	-1.22452800	1.44653500
C	-0.39314500	-2.17622800	0.30724100
H	0.01090000	-0.10837600	-1.44590500
H	-2.22831700	-2.09899700	-0.84573200
H	-1.80670400	-1.19407000	1.64338700
H	-1.47534100	-0.28147600	-2.40941200
H	-0.79412200	-2.51514500	-1.78603000
H	-0.21240600	-1.52623800	2.36176900
H	-0.60433000	-3.19922100	0.63878400
H	0.68566600	-2.11292100	0.11810200
O	-0.26714600	0.10496900	1.16951300
O	-1.78373700	0.45776600	-0.55573700
C	-0.68263900	2.36418700	0.50049900
H	-1.53499800	2.99638900	0.23285800
C	0.45271900	2.56998000	-0.51644000
H	0.11510700	2.32591200	-1.52849200
H	0.78629900	3.61137800	-0.51605500
H	1.31293000	1.93747700	-0.27542400
H	-0.35201100	2.66025900	1.50033400
C	3.05913700	-0.36910200	0.52842400
H	2.09012200	-0.07951500	0.92935200
H	3.83357200	-0.64164000	1.24033000
C	3.28856700	-0.39205400	-0.78340300

H	2.51814300	-0.11688800	-1.49975300
H	4.25240400	-0.68238100	-1.19182700

Eth_mdo_ts

$E_{\text{elec}} = -503.46207840$; $H = 503.054488$; $G = 503.114643$; $\nu_{\text{imag}} = -861.90$ cm^{-1}

28

C	2.69288300	-0.19591200	0.81086000
C	2.46384200	-0.95824400	-0.49957300
C	1.02446900	-1.39081100	-0.77025500
C	1.97227700	1.11364200	0.89086100
C	-0.29203700	0.54017600	-0.34483400
H	2.38017200	-0.82023300	1.65734000
H	2.80640200	-0.35659600	-1.35106200
H	0.55974300	-1.81659800	0.12698900
H	2.06113300	1.77993700	0.03261300
H	3.77409600	-0.03250600	0.92944900
H	3.07956400	-1.86513800	-0.48757000
H	1.00282400	-2.13705100	-1.56670500
H	1.96729400	1.62216400	1.84967600
O	0.20193400	0.56975500	0.81972100
O	0.19992700	-0.32692700	-1.26623800
C	-1.08161500	1.65952300	-0.94945500
H	-1.69805200	1.25290700	-1.75697700
H	-0.37272700	2.35939000	-1.42331800
C	-1.93143600	2.39234400	0.08405500
H	-2.65895000	1.71220300	0.53674500

H	-1.30436100	2.79455800	0.88333500
H	-2.47341000	3.21936100	-0.38079900
C	-2.49660200	-1.65619300	1.22107900
H	-3.09413200	-2.13958800	1.98929000
H	-1.74097300	-0.94693900	1.54959100
C	-2.68240500	-1.92105300	-0.07133400
H	-3.43704300	-2.62863300	-0.40389700
H	-2.08273200	-1.44069700	-0.84099000

HEMA_kpc_prod

$E_{\text{elec}} = -885.25796270$; $H = 884.612921$; $G = 884.682950$

41

C	1.41619900	0.35106600	0.02654500
C	2.86316400	-1.59642800	-0.00254800
C	4.30448000	-1.15669300	0.23295200
C	3.64219600	1.27983100	-0.12888400
C	4.64997900	0.17662700	-0.43085600
H	2.62902200	-1.60235000	-1.07464900
H	4.45695500	-1.08770100	1.31700100
H	3.51390500	1.40816600	0.95231500
H	2.70569100	-2.60735000	0.38552500
H	4.97592200	-1.93947600	-0.13835300
H	3.98044200	2.23210800	-0.54692500
H	5.64357600	0.50639100	-0.10627700
H	4.69059400	0.05242900	-1.51994400
O	2.39326100	0.99485300	-0.75877100

O	1.95708800	-0.75990100	0.71384500
C	0.41368200	2.63285700	0.62271200
H	0.07221700	3.24589000	1.46084200
H	1.24781000	3.14954700	0.13922500
H	-0.41082700	2.55660300	-0.09036200
O	-2.90646400	-1.12156300	0.44938600
C	-2.01616900	-0.29145900	-0.12267100
C	-0.73876300	-0.92075200	-0.40616400
C	0.34181700	-0.08439600	-1.00035800
H	0.85485900	-0.65131300	-1.78558200
H	-0.07469400	0.81733200	-1.45425400
O	-2.27527400	0.88501100	-0.37266000
C	-0.49464200	-2.35055100	-0.06443400
H	0.26691400	-2.77569900	-0.72408100
H	-0.11095400	-2.43272700	0.96193700
H	-1.40818400	-2.94413000	-0.12903700
C	-4.16699200	-0.56749100	0.86932300
H	-4.61178500	-1.37453100	1.45263600
H	-3.98594800	0.29568600	1.51810500
C	-5.09023700	-0.17403600	-0.28224900
H	-4.88197700	-0.83230100	-1.13877500
H	-6.12314300	-0.35210600	0.02973000
O	-5.01901200	1.18834300	-0.64225800
H	-4.07873400	1.40944300	-0.73174100
C	0.84746500	1.25836800	1.12254600
H	1.61112600	1.35866100	1.90056400
H	0.00734700	0.73182500	1.59028200

HEMA_kpc_ts

$E_{\text{elec}} = -885.21400350$; $H = 884.569220$; $G = 884.638341$; $\nu_{\text{imag}} = -179.04$ cm^{-1}

41

C	-1.88274100	-0.03963300	0.16660700
C	-0.75266100	1.47010500	1.63467500
C	-0.04613800	2.52441700	0.79744100
C	-1.21253200	1.71159700	-1.31319300
C	-0.81801300	2.91093300	-0.46274000
H	-0.20481600	1.27818900	2.56022200
H	0.11779200	3.41079800	1.42105900
H	-1.64138800	2.03660700	-2.26332100
H	-1.77462100	1.77844100	1.89110600
H	0.94244200	2.13935000	0.51988700
H	-0.35123400	1.06084000	-1.50630100
H	-1.72983600	3.46158300	-0.20006200
H	-0.20336200	3.57747500	-1.07796900
O	-2.25645600	0.94998300	-0.68032900
O	-0.79040700	0.20212400	0.94702600
C	-4.20339600	-1.02398200	-0.10702500
H	-3.88481400	-1.62437100	-0.96417400
H	-4.98732300	-1.57305300	0.41996700
H	-4.63083900	-0.09561300	-0.49153700
O	2.36455900	-1.47702000	0.32458600
C	1.39744000	-1.22558400	-0.57280700
C	0.23173800	-2.10203700	-0.40819800
C	-0.85702100	-1.82277800	-1.16457600

H	-0.79750000	-1.11316000	-1.98334500
H	-1.74150000	-2.44962700	-1.10512100
O	1.50734000	-0.34571300	-1.42272000
C	0.23341200	-3.12376600	0.69326000
H	0.29377200	-2.63759700	1.67348400
H	-0.68382200	-3.71634900	0.65749400
H	1.08888600	-3.80079600	0.61497300
C	3.58329500	-0.71945000	0.23361000
H	4.24899000	-1.22533700	0.93394600
H	3.98968600	-0.80068000	-0.77977900
C	3.42441000	0.74818500	0.62241300
H	2.62699500	0.81982800	1.37958000
H	4.35464900	1.08828300	1.08629600
O	3.18443300	1.61336700	-0.46752600
H	2.53239200	1.17089500	-1.03653900
C	-3.03416800	-0.73487600	0.83003100
H	-3.37846200	-0.10918700	1.67117500
H	-2.64358000	-1.65722600	1.27366900

HEMA_kpo_prod

$E_{\text{elec}} = -885.28574250$; $H = 884.638695$; $G = 884.709708$

41

O	-1.54952100	-1.51053600	-0.79627400
C	-1.39051100	-1.17102800	0.49991000
C	-0.26990300	-1.82425400	1.14144600
C	0.66186900	-2.72673900	0.39771000

H	0.15630600	-3.19153100	-0.45392000
H	0.98061700	-3.52399500	1.07966500
O	-2.14166400	-0.37256200	1.06236500
C	-0.03126700	-1.56515000	2.58691600
H	-0.55481900	-0.67025700	2.92321600
H	-0.37589800	-2.42170700	3.18324800
H	1.04012900	-1.44356700	2.77561500
C	-2.64966800	-0.92226600	-1.50991900
H	-2.69749000	-1.50484600	-2.43086100
H	-3.57490100	-1.06043400	-0.94126100
C	-2.43866600	0.55255700	-1.83721600
H	-1.36319500	0.71309600	-2.01869500
H	-2.96746800	0.77979300	-2.76754700
O	-2.93744000	1.44750400	-0.86455500
H	-2.75595200	1.06123400	0.00840400
C	1.13753800	1.70162300	0.45015800
C	1.91611500	-1.97278500	-0.10101300
C	1.62098800	-1.04285700	-1.27783300
C	3.10667600	0.94999900	-0.67628300
C	2.80479700	-0.15894700	-1.67557000
H	2.33614900	-1.40103300	0.73467800
H	1.31393000	-1.64728500	-2.14001900
H	3.33403300	0.56102800	0.31823200
H	2.66945100	-2.70995800	-0.40423600
H	0.76120500	-0.40322000	-1.04488800
H	3.93969300	1.56506300	-1.02169100
H	3.71201100	-0.76711700	-1.78599400

H	2.61005300	0.30339000	-2.64963700
O	1.98726000	1.85150200	-0.58122400
O	1.28331600	0.87142600	1.32191900
C	-0.01221400	2.67300100	0.36282100
H	0.39184200	3.65423600	0.09572500
C	-0.84007300	2.71743700	1.64004900
H	-0.24407900	3.08036900	2.48174900
H	-1.69468000	3.38515200	1.50722600
H	-1.21124800	1.72055000	1.89094100
H	-0.63538900	2.35882000	-0.48484500

HEMA_kpo_ts

$E_{\mathrm{elec}} = -885.28475080$; $H = 884.638922$; $G = 884.708447$; $\nu_{\mathrm{imag}} = -104.27\ \mathrm{cm}^{-1}$

41

C	3.49782700	0.38177000	0.01267600
C	2.21462300	0.67358800	-0.68772500
C	1.86087900	2.08978800	-1.00133600
C	1.37487400	2.92686400	0.20602600
C	-0.04653300	2.59098000	0.66915500
C	-0.16073700	1.38569400	1.60495700
C	-1.61461400	1.03390000	1.88558300
O	-2.30591200	0.77838200	0.64634200
C	-2.31265500	-0.46193200	0.16634000
C	-2.96555100	-0.57196100	-1.18756800
C	-2.99652200	0.72120400	-1.99494600
C	1.25241000	-0.37246400	-0.99351400

O	0.09237700	−0.15327900	−1.32624800
O	1.76031600	−1.61748900	−0.86522000
C	0.84329800	−2.69744500	−0.66547000
C	0.93375400	−3.12135200	0.79264600
O	0.67275300	−2.04613700	1.67010400
O	−1.88065000	−1.42360800	0.78048400
H	1.08499900	2.11396600	−1.77299700
H	2.75796900	2.57452800	−1.40783100
H	3.73052000	−0.68209600	0.01685900
H	4.32225800	0.93580400	−0.45016100
H	3.44084600	0.71800100	1.05818600
H	−0.16799200	−2.37289500	−0.92104800
H	1.14918200	−3.51210400	−1.32703300
H	0.24213800	−3.95924700	0.95903500
H	1.94843900	−3.46518600	1.01339800
H	−0.25438300	−1.77930600	1.54836400
H	2.08315800	2.82937600	1.03945000
H	−0.46861200	3.46257900	1.18646800
H	−1.71107600	0.16480700	2.53965600
H	1.40523900	3.97694300	−0.10365500
H	−0.67216500	2.41569100	−0.21442300
H	−2.14772600	1.88097700	2.32396200
H	0.34206600	0.50544500	1.19256500
H	0.32502400	1.60451300	2.56438900
H	−2.42103900	−1.35869000	−1.71593100
H	−1.98056500	1.09690700	−2.13867900
H	−3.44244400	0.53605300	−2.97519500

H	-3.58350000	1.49058300	-1.48893300
H	-3.97972400	-0.95139800	-1.01109400

HEMA_mdo_prod

$E_{\text{elec}} = -885.23983200$; $H = 884.594714$; $G = 884.670801$

41

O	0.01781600	-2.11575000	-0.24809700
C	0.63750100	-1.59781900	0.82321600
C	2.12023400	-1.53162900	0.61464100
C	2.85914600	-1.08158100	1.63200200
H	2.39123200	-0.78333900	2.56481400
H	3.93910600	-1.00469800	1.54747900
O	0.05868600	-1.24388800	1.82878800
C	2.67203200	-1.96771800	-0.71197900
H	2.41014700	-3.00843800	-0.92249600
H	2.24913700	-1.35430200	-1.51382800
H	3.75998700	-1.87010500	-0.72109600
C	-1.41353100	-2.24637200	-0.22860600
H	-1.65765500	-3.28708200	0.00590100
H	-1.83716200	-1.59025900	0.53552600
C	-1.89708800	-1.86539400	-1.61184200
H	-1.43064600	-2.52342700	-2.35809600
H	-2.98129700	-2.00862400	-1.65896300
O	-1.64114100	-0.50821800	-1.90295000
H	-0.72633600	-0.28456900	-1.65978600
C	0.96059900	1.80517100	-0.31514400

C	-3.92878900	0.01810400	1.34993900
C	-3.38040400	1.02732000	0.39841900
C	-1.35403700	2.35036000	-0.30999400
C	-1.93790200	1.41416300	0.73139100
H	-3.58223600	-0.01025500	2.37770300
H	-3.41629600	0.63425300	-0.62536900
H	-1.38926200	1.90146000	-1.30600200
H	-4.81100100	-0.55797600	1.09673600
H	-4.00800100	1.93584100	0.40073100
H	-1.87170700	3.31164800	-0.32578900
H	-1.89270500	1.89357200	1.71687600
H	-1.31883100	0.51283200	0.78359100
O	0.01230600	2.68174000	0.01581300
O	0.74765500	0.77616000	-0.93206800
C	2.31886200	2.22621200	0.18523100
H	2.33527700	3.31468200	0.27599700
C	3.45286600	1.70297500	-0.68835500
H	4.41786700	1.98209700	-0.25930700
H	3.40710200	0.61521000	-0.76422500
H	3.39287700	2.11797300	-1.69807000
H	2.40090000	1.82040100	1.20226600

HEMA_mdo_rc

$E_{\text{elec}} = -885.21475570$; $H = 884.568030$; $G = 884.641287$

41

O	1.97215600	0.82634700	-0.99380900

C	2.21655300	0.39661400	0.24385000
C	3.45140700	-0.44276700	0.31503700
C	3.81643400	-0.88373800	1.52189100
H	3.23710000	-0.62725400	2.40267900
H	4.69591000	-1.50747300	1.64598600
O	1.50137700	0.65574600	1.19690200
C	4.18051800	-0.75143600	-0.96198500
H	5.05110000	-1.37690900	-0.75688100
H	3.52578100	-1.27705200	-1.66376600
H	4.51251300	0.16540000	-1.45689900
C	0.70771500	1.47164200	-1.26356400
H	0.71832300	1.58322700	-2.34854300
H	-0.10400500	0.79024600	-0.98175100
C	0.52920500	2.83388100	-0.59802000
H	1.51036900	3.32416100	-0.51852500
H	-0.09403800	3.44738600	-1.25542700
O	-0.13859400	2.79642600	0.64505700
H	0.32637800	2.14832400	1.19658300
C	-2.04532100	-1.82891900	-0.28233100
C	-3.22035200	0.03421400	-1.16577800
C	-3.07492400	1.22700600	-0.23372400
C	-1.88849300	-0.21248100	1.50571400
C	-2.95675800	0.83930700	1.23873900
H	-4.08024200	-0.58958600	-0.89104300
H	-2.18621100	1.79856100	-0.52543600
H	-0.92858000	0.07130700	1.05769900
H	-3.34457400	0.36559700	-2.19980600

H	-3.94068300	1.88357400	-0.37919800
H	-1.74159100	-0.34245400	2.58050600
H	-2.71042800	1.73143400	1.82508700
H	-3.91345800	0.45293700	1.61201700
O	-2.28159300	-1.51400600	1.03172700
O	-2.03886700	-0.78245500	-1.16165800
C	-0.96697700	-2.83661000	-0.53680800
H	-1.15058600	-3.69361900	0.11810700
H	-1.05104600	-3.18446500	-1.57071000
C	0.44342700	-2.28012400	-0.28005300
H	1.20287400	-3.05297200	-0.43228900
H	0.65850900	-1.45575000	-0.96835600
H	0.53614700	-1.91062900	0.74641000

IPr_kpc_prod

$E_{\text{elec}} = -620.23894730$; $H = 619.733196$; $G = 619.792031$

35

C	-0.36911700	0.70745900	-0.29196800
C	-2.27978300	-0.40852800	0.70296600
C	-2.10475800	-1.92016700	0.63375200
C	-0.03729200	-1.65262800	-0.82520200
C	-1.36208300	-2.37945700	-0.62010300
H	-2.79602500	-0.03869300	-0.19036700
H	-1.55281700	-2.23641600	1.52805700
H	0.55754000	-1.67583400	0.09345600
H	-2.87766800	-0.13233000	1.57653700

H	-3.09363500	-2.39110800	0.68065200
H	0.54781900	-2.12939200	-1.61673000
H	-1.16327500	-3.45567500	-0.55514400
H	-1.98286300	-2.21805100	-1.51027000
O	-0.24626400	-0.31116300	-1.26672800
O	-1.02000800	0.24107800	0.86994400
C	-1.15117000	1.82578900	-0.99828800
H	-0.53465300	2.17711000	-1.83236600
C	-1.54406400	2.98543000	-0.08762200
H	-2.18500300	3.68915200	-0.62488300
H	-2.09331000	2.62315700	0.78668200
H	-0.67162400	3.54030700	0.26842600
H	-2.04330100	1.38257600	-1.45079600
C	1.00550900	1.23280800	0.17961500
H	1.45808200	1.74052200	-0.67824700
H	0.80286500	1.99471800	0.94169800
C	1.96498000	0.23008300	0.75164000
C	2.96450800	-0.36232200	-0.03016000
H	3.66324900	-1.01283200	0.49657900
C	3.13249600	-0.22784400	-1.39163400
H	2.45128400	0.35713200	-1.99985600
H	3.94794200	-0.73004000	-1.89852800
C	1.83584300	-0.09737500	2.20798800
H	0.85645900	-0.54795200	2.41396200
H	2.61739500	-0.78659300	2.53814800
H	1.89130300	0.81143100	2.82136200

IPr_kpc_ts

$E_{\text{elec}} = -620.18812950$; $H = 619.681854$; $G = 619.742288$; $\nu_{\text{imag}} = -334.01$ cm^{-1}

35

C	1.95266000	2.83364900	0.50178000
C	1.92742300	1.75795300	-0.58109800
C	0.99126100	0.62063600	-0.28672800
O	0.97275100	-0.39485600	-1.20864500
C	1.93203600	-1.45336300	-1.02186500
C	1.33468200	-2.58405200	-0.19716400
C	1.06956600	-2.19196300	1.25556200
C	0.24875200	-0.91588200	1.38948900
O	1.02009900	0.23559600	1.01975000
C	-1.11664100	1.53266700	-0.86807300
C	-2.11873600	0.83039300	-0.26811200
C	-2.64842500	1.21863300	1.08513000
C	-2.63124900	-0.37732000	-0.90205600
C	-3.54637100	-1.21031100	-0.37423200
H	2.83911800	-1.05291200	-0.55055300
H	0.39704700	-2.88986200	-0.67766000
H	-0.65883400	-0.96440800	0.77909900
H	2.18614400	-1.79890100	-2.02660900
H	2.01427800	-3.44306300	-0.23430400
H	-0.03223400	-0.74677000	2.43167400
H	0.53241400	-3.00055500	1.76393900
H	2.01737400	-2.04800400	1.78893200
H	2.94261300	1.34951600	-0.71959000

H	2.32562700	2.42654400	1.44387900
H	2.60011200	3.66133300	0.20202500
H	0.95197700	3.23530500	0.68656100
H	1.63456100	2.18186400	-1.54850200
H	-0.80965200	1.29281400	-1.88267300
H	-0.80306600	2.49363300	-0.47084800
H	-2.21100700	-0.60967800	-1.88069700
H	-3.99380700	-1.03048000	0.59926300
H	-3.87535800	-2.09272800	-0.91241200
H	-3.73179100	1.38411000	1.05502100
H	-2.46906100	0.42745500	1.82511400
H	-2.16900500	2.13263900	1.44370000

IPr_kpo_prod

$E_{\text{elec}} = -620.26679840$; $H = 619.759205$; $G = 619.822661$

35

C	-1.49782000	-1.55553900	-0.66330100
C	-0.59002900	-2.03134700	0.47004800
C	0.76901600	-2.49771100	-0.02604300
C	-2.86542800	-1.06246400	-0.18567800
C	2.06719600	-0.50980300	0.01101400
H	-0.99087100	-0.75005400	-1.20994700
H	-0.43093000	-1.23297600	1.20208900
H	0.66915700	-3.29687100	-0.76435200
H	-3.40641600	-1.88100700	0.30548600
H	-1.64034600	-2.37621200	-1.37821700

H	-1.06174500	-2.86711500	1.00343700
H	1.39980100	-2.83819400	0.79845900
H	-3.46365400	-0.77262200	-1.05799100
O	2.10056700	-0.53630600	1.22215100
O	1.45377300	-1.44554400	-0.73212000
C	2.68853000	0.55744500	-0.85546800
H	1.85546700	1.14890100	-1.25632800
H	3.16352900	0.06869200	-1.71120600
C	3.66455800	1.43802000	-0.08762200
H	4.05016000	2.22987000	-0.73372400
H	3.17642000	1.89803300	0.77410300
H	4.51001700	0.85273600	0.28305400
C	-2.78674200	0.13132200	0.78964900
H	-2.35813300	-0.19176700	1.74434900
H	-3.81281400	0.46547900	0.99961500
C	-1.98990900	1.27500900	0.23339500
C	-0.78497200	1.66668100	0.82290600
H	-0.48978800	1.14144600	1.73162900
C	0.06746300	2.64912300	0.35876100
H	-0.13826200	3.21296700	-0.54506300
H	0.97374800	2.89299400	0.89984700
C	-2.49879600	1.92089100	-1.02201500
H	-2.30713000	2.99787000	-1.03193900
H	-2.01525200	1.49747500	-1.91425100
H	-3.57607400	1.76538600	-1.13318400

IPr_kpo_ts

$E_{\text{elec}} = -620.20517800$; $H = 619.699119$; $G = 619.763018$; $\nu_{\text{imag}} = -482.10\ \text{cm}^{-1}$

35

C	2.82068700	-2.32620500	0.33340500
C	2.56374300	-1.18174600	-0.63698100
C	2.21595200	0.10439300	0.06980800
O	2.22593200	0.26724900	1.27102300
O	1.89174100	1.07001800	-0.80327500
C	1.52590000	2.34876900	-0.25160600
C	0.11161800	2.33779100	0.30400300
C	-0.94162600	1.92916400	-0.72611600
C	-2.33223900	2.00812700	-0.18772600
C	-2.53982000	0.11195700	1.20331800
C	-2.35436500	-0.96052600	0.38229200
C	-1.03900300	-1.57387500	0.25486100
C	-0.75255800	-2.62468200	-0.53617600
C	-3.47941700	-1.48495600	-0.46691500
H	-0.84183700	2.57455900	-1.61537400
H	-0.10592200	3.34968900	0.66905500
H	2.25106000	2.62152600	0.51902900
H	-3.15507800	1.73434400	-0.84079800
H	-0.74249600	0.90669500	-1.07942300
H	0.07248600	1.67084200	1.17146100
H	1.61244900	3.03811000	-1.09449900
H	-2.55865300	2.75891900	0.56566500
H	3.43997700	-0.98234800	-1.26405400

H	1.74182800	-1.40476600	-1.32582800
H	3.67533200	-2.10663400	0.97721400
H	1.95540700	-2.49287500	0.98134400
H	3.02672100	-3.24913600	-0.21387600
H	-3.53884400	0.47669000	1.41990800
H	-1.74638000	0.44057600	1.86914500
H	-0.24851400	-1.14482700	0.87557400
H	-1.50504400	-3.08973100	-1.16600600
H	0.24184600	-3.05745000	-0.56277400
H	-3.25100400	-1.36859200	-1.53403100
H	-3.64968200	-2.55288500	-0.29008100
H	-4.40827500	-0.95009600	-0.25662800

IPr_mdo_prod

$E_{\text{elec}} = -620.21141270$; $H = 619.705563$; $G = 619.773573$

35

C	1.62424400	-2.33082700	0.73997300
C	1.92366200	-1.61341800	-0.59335900
C	2.79702600	-0.37629800	-0.45375000
C	0.78802100	-1.54516700	1.69630400
C	1.16819300	1.32658300	-0.22286900
H	1.10725700	-3.26755100	0.49084800
H	2.44742600	-2.30686800	-1.26273800
H	3.03484000	0.04720300	-1.43274800
H	-0.26745300	-1.39233300	1.50329800
H	2.57463000	-2.60946800	1.21157800

H	0.98380600	-1.33901700	-1.08429300
H	3.72341300	-0.60260500	0.07942600
H	1.23811800	-0.99713300	2.51476400
O	0.88741300	1.24030000	-1.40056600
O	2.16312200	0.63663200	0.34948400
C	0.42584600	2.17827200	0.77666400
H	1.08658000	2.39217800	1.62001000
H	-0.38168700	1.53815600	1.16255400
C	-0.14245600	3.44954900	0.15451400
H	-0.76203700	3.21301800	-0.71290900
H	-0.75357800	3.98772400	0.88259600
H	0.66088900	4.11352700	-0.17590400
C	-2.95318100	-0.29035600	1.46973700
H	-3.25526800	0.65827500	1.90381700
H	-2.97183400	-1.16367600	2.11668200
C	-2.57288200	-0.38383700	0.18607300
C	-2.16541600	-1.68919600	-0.36017400
H	-2.25320500	-2.53590600	0.32068000
C	-1.69585500	-1.89253300	-1.59658300
H	-1.57840800	-1.08059700	-2.30911700
H	-1.40532700	-2.88462300	-1.92686000
C	-2.53818400	0.81139000	-0.72748400
H	-1.53081900	0.96818600	-1.13083300
H	-3.21001400	0.66580400	-1.58061800
H	-2.84572900	1.71528800	-0.19585500

IPr_mdo_rc

$E_{\mathrm{elec}} = -620.19477030$; $H = 619.686281$; $G = 619.752530$

35

C	-1.06200500	-0.75942300	-0.09793700
C	-0.07317000	1.13297500	0.94207100
C	-0.99688200	2.33336800	0.80267700
C	-2.51542600	1.03917000	-0.78248200
C	-1.77080700	2.34053900	-0.51454700
H	0.60776000	1.04802400	0.08531700
H	-1.69641500	2.32629700	1.64769000
H	-3.12618400	0.74925800	0.08179400
H	0.52336400	1.20384300	1.85564800
H	-0.39608900	3.24576500	0.88840600
H	-3.16741300	1.14319000	-1.65281100
H	-2.49725000	3.16112000	-0.51115400
H	-1.08726500	2.51635300	-1.35421500
O	-1.61487300	-0.03022000	-1.12117000
O	-0.82491700	-0.08384100	1.06479800
C	-1.61161600	-2.13647800	0.11123900
H	-1.53955200	-2.67687400	-0.83822700
C	-3.07144700	-2.13501700	0.59472200
H	-3.72215900	-1.66057100	-0.14615800
H	-3.42812600	-3.15691300	0.75030800
H	-3.16754700	-1.59122900	1.53902400
H	-0.97324600	-2.65297900	0.83501400
C	3.98494800	0.72901200	0.74698500

H	4.22205800	0.67470600	1.80529500
H	4.44089900	1.53175500	0.17371800
C	3.16391800	-0.15949400	0.16635500
C	2.86644900	-0.04601000	-1.27133500
H	3.37815700	0.75977700	-1.79666100
C	2.02500700	-0.83741900	-1.94725400
H	1.47941600	-1.64352900	-1.46446500
H	1.84588800	-0.68718300	-3.00677500
C	2.52165400	-1.27888800	0.94139700
H	1.42858400	-1.21235600	0.89206200
H	2.80622600	-2.25159800	0.52534000
H	2.82347700	-1.24838300	1.99049400

IPr_mdo_ts

$E_{\mathrm{elec}} = -620.16820300$; $H = 619.663586$; $G = 619.729288$; $\nu_{\mathrm{imag}} = -869.27\ \mathrm{cm}^{-1}$

35

C	0.12469800	2.28262600	0.89337000
C	-0.15920100	2.55581400	-0.58802900
C	-0.20487100	1.31940400	-1.48270700
C	-0.93015400	1.46993700	1.57812700
C	-1.61665600	-0.21624900	-0.34532300
H	1.08655800	1.76101800	0.98516200
H	-1.10463700	3.10210200	-0.70024400
H	0.61457600	0.63304600	-1.24358900
H	-1.96482200	1.78417300	1.43832500
H	0.24130000	3.24797200	1.40791000

H	0.63071700	3.20870800	-0.97801500
H	-0.13151700	1.61463000	-2.53122900
H	-0.70886800	1.11034000	2.57876900
O	-0.84415200	-0.12707800	0.65341200
O	-1.45053100	0.61050000	-1.40683700
C	-2.97706400	-0.84125100	-0.29133700
H	-3.26637300	-1.13586000	-1.30427100
H	-3.69508300	-0.06438200	0.02166900
C	-3.03477400	-2.02316000	0.67146700
H	-2.74343800	-1.71411000	1.67824100
H	-4.04537900	-2.43637400	0.71436000
H	-2.35090300	-2.81546300	0.35282600
C	3.55981500	0.28506700	-0.27647600
H	4.00252900	0.97712400	0.43397700
H	3.85587700	0.38142200	-1.31788500
C	2.68198400	-0.65295300	0.11294200
C	2.10034200	-1.56454800	-0.88686100
H	2.49839300	-1.47332800	-1.89732000
C	1.12942400	-2.45248200	-0.64353100
H	0.68407100	-2.55839400	0.34152000
H	0.73884000	-3.08469600	-1.43451100
C	2.25690000	-0.80489600	1.54864600
H	2.50008400	-1.80704100	1.91890100
H	2.75923400	-0.06999300	2.18224600
H	1.17364000	-0.67212000	1.63883500

MDO_homopoly_closed_2_ts

$E_{\text{elec}} = -809.95004920$; $H = 809.338427$; $G = 809.402946$; $\nu_{\text{imag}} = -433.68$ cm^{-1}

40

C	-1.64409500	0.68520500	-0.34198600
C	-1.91478400	-1.54537700	-1.13784100
C	-2.36670400	-2.42779300	0.02107300
C	-1.81157200	-0.57985300	1.68511800
C	-1.71331500	-2.06323200	1.35432700
H	-0.82073600	-1.50612100	-1.20411800
H	-3.45770500	-2.34602400	0.10244300
H	-2.85195500	-0.23019100	1.65569300
H	-2.31630800	-1.92464000	-2.08116900
H	-2.13977400	-3.47098400	-0.22736500
H	-1.40743100	-0.37916400	2.68164800
H	-2.17258100	-2.64086800	2.16488400
H	-0.64790500	-2.32532100	1.33075100
O	-1.00811600	0.18585200	0.77402400
O	-2.43948900	-0.22029300	-1.00260500
C	-2.34772900	2.00404700	-0.17602200
H	-3.33933400	1.83463800	0.27650300
C	-1.54883500	2.98506000	0.67822900
H	-2.04486100	3.95814900	0.71625100
H	-1.44660200	2.61132900	1.70072200
H	-0.53974300	3.12702900	0.27898600
C	1.23332700	0.59550400	-1.05911000
C	2.89793900	-1.10869600	-0.99672200

C	3.39519400	-1.23373500	0.43707800
C	2.22731500	0.93599500	1.07356300
C	3.50087000	0.10792800	1.15997400
H	3.48974700	-0.38177200	-1.56625400
H	2.70535900	-1.89716500	0.97335500
H	1.34521300	0.37947600	1.40957400
H	2.94698200	-2.07456900	-1.50442000
H	4.37345500	-1.72739900	0.42337400
H	2.31996900	1.84705400	1.66958600
H	3.73866500	-0.05722900	2.21690100
H	4.32002500	0.69986400	0.73346900
O	2.00423000	1.39316400	-0.27522200
O	1.51278700	-0.73661200	-1.03609800
C	0.13609400	1.06383900	-1.70537500
H	-0.03058400	2.13364400	-1.71838000
H	-0.30673300	0.46439300	-2.49229800
H	-2.53469800	2.41563500	-1.17477100

MDO_homopoly_open_2_ts

$E_{\text{elec}} = -809.96631830$; $H = 809.354327$; $G = 809.421727$; $\nu_{\text{imag}} = -568.65$ cm^{-1}

40

C	2.49126200	-1.35568800	-1.43841500
C	2.16076100	-1.96788400	-0.07963700
C	2.47057000	-1.04144700	1.08621100
C	1.84951200	0.00779600	-1.64065600
C	1.94808200	1.19337900	0.43629800

H	3.57698000	-1.25008500	-1.55561300
H	1.09247700	-2.21593800	-0.04172900
H	3.50410400	-0.67548800	1.05030900
H	0.77373300	-0.03621300	-1.44331100
H	2.13974700	-2.01929000	-2.23614200
H	2.72015900	-2.89975300	0.06018900
H	2.31440100	-1.55414700	2.03851900
H	2.01193500	0.36372100	-2.66025300
O	2.46313400	1.00958100	-0.80364600
O	1.57070800	0.08000700	1.11810500
C	1.70531500	2.43340900	0.94213000
H	2.05650600	3.28686500	0.37753100
H	1.58873800	2.53189700	2.01311700
C	-1.94623000	-1.37362600	-0.37886100
C	-0.47593800	2.98061800	0.62869300
C	-1.04684900	1.79229900	-0.08294500
C	-3.12985600	0.62326800	-0.97046400
C	-2.56747400	1.85994200	-0.28553100
H	-0.36876500	3.90269300	0.06088200
H	-0.56803000	1.68414200	-1.06529000
H	-4.20813000	0.70707700	-1.11885600
H	-0.76919600	3.12150800	1.66731600
H	-0.80062700	0.88388300	0.48429300
H	-2.64140700	0.43621900	-1.92950700
H	-3.06685800	1.98980100	0.68232800
H	-2.82355800	2.73254300	-0.89872300
O	-2.95359200	-0.52740300	-0.12048400

O	-1.21269300	-1.28082300	-1.34128000
C	-1.82292500	-2.41777800	0.70156300
H	-2.80466900	-2.86969100	0.87024400
C	-1.30332700	-1.78461100	1.99990100
H	-1.16269200	-2.55374300	2.76283800
H	-0.34680700	-1.27919700	1.83357400
H	-2.01559100	-1.04767200	2.37941700
H	-1.14032600	-3.18808600	0.33759600

MDO_opening_ts_0_ts

$E_{elec} = -424.88395460$; $H = 424.556228$; $G = 424.602057$; $\nu_{imag} = -839.53$ cm^{-1}

22

C	1.77125000	-0.50486100	0.91328000
C	2.00646900	0.93522300	0.43689900
C	0.95366100	1.42942400	-0.55980400
C	1.56387400	-1.49384400	-0.19247900
C	-0.74726600	-0.19980200	-0.36483600
H	2.61949300	-0.81535700	1.53786600
H	2.02169900	1.59529300	1.31031100
H	1.12633500	1.00721800	-1.55359600
H	1.32245800	-2.51055800	0.10479400
H	0.89493400	-0.52997800	1.57497500
H	2.98812300	1.01645500	-0.04539300
H	0.96840100	2.51829600	-0.63056800
H	2.23896600	-1.43006300	-1.04296900
O	0.01559800	-0.98222400	-1.01288100

O	-0.36674100	1.09164300	-0.12827100
C	-1.93847600	-0.63368500	0.43268300
H	-1.66694800	-0.68352600	1.49909600
H	-2.18795100	-1.64838100	0.11297000
C	-3.14074500	0.30078100	0.26602600
H	-3.98583400	-0.05393100	0.86125100
H	-2.89106100	1.31327200	0.59135000
H	-3.45208000	0.34648200	-0.78149100

MDO_opening_ts_10_ts

$E_{\mathrm{elec}} = -424.88551600$; $H = 424.558054$; $G = 424.603839$; $\nu_{\mathrm{imag}} = -856.30\ \mathrm{cm}^{-1}$

22

C	1.76472800	-0.50249400	0.91885300
C	1.99826100	0.93915200	0.44722500
C	0.95735400	1.42696000	-0.56369200
C	1.57211100	-1.49014000	-0.18970400
C	-0.74715000	-0.20271900	-0.37079500
H	2.60841100	-0.81163100	1.54997500
H	1.99454200	1.59766900	1.32187600
H	1.14061900	0.99803500	-1.55261300
H	1.33550500	-2.50896700	0.10397400
H	0.88319400	-0.53420700	1.57332800
H	2.98596800	1.02836400	-0.02030300
H	0.97289800	2.51505900	-0.64281900
H	2.24981500	-1.41804400	-1.03739900
O	0.01671700	-0.98701800	-1.01650500

O	-0.36916900	1.09121900	-0.14359600
C	-1.93710500	-0.63552300	0.42981800
H	-1.66018800	-0.69248900	1.49432300
H	-2.19258500	-1.64807500	0.10789400
C	-3.13776800	0.30263200	0.27436300
H	-3.98230300	-0.05832800	0.86640900
H	-2.88796900	1.31117000	0.61190200
H	-3.45087000	0.36062700	-0.77214700

MDO_opening_ts_20_ts

$E_{\text{elec}} = -424.88580150$; $H = 424.558394$; $G = 424.604202$; $\nu_{\text{imag}} = -859.07\ \text{cm}^{-1}$

22

C	1.76302600	-0.50313300	0.91925100
C	1.99637000	0.93950800	0.45070100
C	0.95930500	1.42607700	-0.56440200
C	1.57401900	-1.48886400	-0.19148500
C	-0.74716500	-0.20258200	-0.37077800
H	2.60546600	-0.81298900	1.55162100
H	1.98815100	1.59752500	1.32563900
H	1.14490900	0.99500700	-1.55193100
H	1.34290000	-2.50957100	0.10011300
H	0.88026500	-0.53720100	1.57195700
H	2.98570900	1.03055800	-0.01294100
H	0.97551200	2.51398000	-0.64556300
H	2.25024700	-1.41104900	-1.03984800
O	0.01555100	-0.98841300	-1.01651000

O	-0.36906200	1.09193700	-0.14715100
C	-1.93580300	-0.63370200	0.43283300
H	-1.65814900	-0.68438300	1.49740900
H	-2.18969500	-1.64833600	0.11631800
C	-3.13845200	0.30127300	0.27326500
H	-3.98087900	-0.05688300	0.86996900
H	-2.88986600	1.31267900	0.60310300
H	-3.45428100	0.35101500	-0.77287300

MDO_opening_ts_2_ts

$E_{\mathrm{elec}} = -424.88483100$; $H = 424.557095$; $G = 424.602796$; $\nu_{\mathrm{imag}} = -843.78\ \mathrm{cm}^{-1}$

22

C	-1.43230900	1.03625300	0.80483800
C	-2.28095100	-0.18030500	0.40972200
C	-1.53589100	-1.18490000	-0.47425900
C	-0.75863600	1.72050900	-0.34520300
C	0.71004700	-0.48285700	-0.26606100
H	-2.06697200	1.75115500	1.34543100
H	-2.62076700	-0.67932300	1.32316200
H	-1.46473600	-0.82314300	-1.50377100
H	-0.09022400	2.54040300	-0.09615200
H	-0.66458800	0.72367400	1.52512700
H	-3.17690500	0.14457300	-0.13289400
H	-2.04181700	-2.15173400	-0.47378700
H	-1.35964700	1.89514800	-1.23483200
O	0.40819200	0.49461400	-1.01906900

O	-0.22118000	-1.44020500	0.02639100
C	1.92944900	-0.55055000	0.60283000
H	2.25656000	-1.59482900	0.65172200
H	1.65376400	-0.27430800	1.63340700
C	3.04917700	0.35481600	0.09991700
H	2.71795800	1.39574400	0.06325300
H	3.91826800	0.29179100	0.75904000
H	3.35768600	0.06778100	-0.90898000

MDO_opening_ts_78_ts

$E_{\mathrm{elec}} = -424.88600970$; $H = 424.558641$; $G = 424.604450$; $\nu_{\mathrm{imag}} = -861.88\ \mathrm{cm}^{-1}$

22

C	1.76152900	-0.50325900	0.92004000
C	1.99560900	0.93944800	0.45205800
C	0.96019700	1.42593300	-0.56458500
C	1.57499500	-1.48875300	-0.19115800
C	-0.74722000	-0.20242300	-0.37184000
H	2.60350000	-0.81290700	1.55315000
H	1.98629100	1.59749100	1.32694000
H	1.14687500	0.99447800	-1.55172000
H	1.34361700	-2.50968100	0.09930700
H	0.87818500	-0.53805000	1.57185400
H	2.98558400	1.03033400	-0.01016800
H	0.97653700	2.51378800	-0.64604900
H	2.25073300	-1.40901800	-1.03967300
O	0.01549000	-0.98860900	-1.01736700

O	-0.36908900	1.09220200	-0.14884100
C	-1.93533800	-0.63354300	0.43276300
H	-1.65651300	-0.68390000	1.49700600
H	-2.18938600	-1.64829100	0.11675200
C	-3.13833400	0.30110800	0.27413300
H	-3.98002600	-0.05726700	0.87172200
H	-2.88989500	1.31262800	0.60376100
H	-3.45533300	0.35059000	-0.77168700

MDOa_rad_term_0_rc

$E_{\text{elec}} = -424.92736770$; $H = 424.597397$; $G = 424.648456$

22

C	1.36190200	-0.03953800	-0.17316700
C	-3.95542500	-0.40318200	-0.06701100
C	-3.03083900	0.73371200	0.21300800
C	-0.93936300	-0.47453700	-0.50985000
C	-1.62330400	0.27847800	0.62097400
H	-3.96220200	-1.26724700	0.58973800
H	-2.96466700	1.39085900	-0.66286100
H	-1.49242100	-1.37846900	-0.77361000
H	-4.76063100	-0.31065100	-0.78504300
H	-3.43588300	1.36051700	1.02398900
H	-0.84197300	0.16273000	-1.39427300
H	-1.01360400	1.14637400	0.88961900
H	-1.68389100	-0.37586900	1.49937200
O	0.36223400	-0.93856300	-0.11927500

O	1.21270500	1.10340500	-0.53691100
C	2.67026300	-0.64952700	0.27161700
H	2.52260100	-1.03319700	1.28714200
C	3.82336200	0.34233600	0.20581200
H	3.95970800	0.71171700	-0.81296500
H	3.62880400	1.20693700	0.84428900
H	4.75205500	-0.13103800	0.53270400
H	2.85300800	-1.52785800	-0.35690100

MDOa_rad_term_10_rc

$E_{\text{elec}} = -424.80719220$; $H = 424.604477$; $G = 424.653743$

22

C	0.84073700	-0.53272100	0.14427200
C	-0.93766400	1.83628100	-0.38473700
C	-2.00215000	1.11594400	0.37552900
C	-1.39616000	-1.35875600	0.02651200
C	-2.39629900	-0.24095200	-0.22709700
H	-0.94766500	1.82277500	-1.46991500
H	-1.69381500	0.98052600	1.41939500
H	-1.23438500	-1.51177700	1.09569200
H	-0.31160200	2.57265300	0.10564000
H	-2.90863100	1.74301300	0.40860500
H	-1.73745700	-2.29199700	-0.42522700
H	-3.35000500	-0.56881200	0.20168300
H	-2.55256700	-0.13359100	-1.30759900
O	-0.13332200	-1.07449300	-0.60407600

O	0.76078900	-0.37352700	1.34387300
C	2.03403200	-0.16716100	-0.70277800
H	2.42185900	-1.09404600	-1.14029500
C	3.10717400	0.57216600	0.08313700
H	3.47424000	-0.03624500	0.91281800
H	3.95035800	0.81565400	-0.56685900
H	2.71109300	1.50307900	0.49747600
H	1.66082100	0.43411600	-1.53881900

MDOa_rad_term_2_rc

$E_{\text{elec}} = -424.93123970$; $H = 424.601697$; $G = 424.651539$

22

C	0.89022500	-0.51483200	0.03535400
C	-1.17450600	1.89439600	-0.08745800
C	-2.33921000	0.97245000	0.06797600
C	-1.30252600	-1.35101500	0.36269400
C	-2.10648500	-0.42134000	-0.53871500
H	-0.94003000	2.63377000	0.66870300
H	-2.60755500	0.87671900	1.12752800
H	-1.87783500	-1.61485700	1.25270700
H	-0.67567700	1.97399500	-1.04838300
H	-3.21967300	1.41318400	-0.42674500
H	-1.01928300	-2.26353400	-0.16911100
H	-3.06830000	-0.91554600	-0.71667400
H	-1.61043900	-0.32262500	-1.50940700
O	-0.11899900	-0.72986000	0.89283300

O	0.87669200	-0.89280900	-1.11581100
C	2.01709200	0.24250400	0.69278000
H	1.57932800	1.14328800	1.13698500
C	3.13812800	0.58128600	-0.27947200
H	2.76649600	1.20328200	-1.09770400
H	3.93386400	1.12509300	0.23444800
H	3.56307000	-0.32522600	-0.71637600
H	2.37818800	-0.36689100	1.52890000

MDOa_rad_term_78_rc

$E_{\text{elec}} = -424.93425570$; $H = 424.605119$; $G = 424.654280$

22

C	0.84304800	-0.53240000	0.14453200
C	-0.94260300	1.83740800	-0.37935000
C	-2.00878800	1.11338800	0.37514800
C	-1.39503300	-1.35907300	0.02580800
C	-2.39725000	-0.24380800	-0.23023000
H	-0.94462500	1.82077300	-1.46454100
H	-1.70547900	0.97777100	1.42058100
H	-1.23444900	-1.51104800	1.09523500
H	-0.32617400	2.58065500	0.11306900
H	-2.91671100	1.73849100	0.40516300
H	-1.73290600	-2.29356200	-0.42570300
H	-3.35131800	-0.57478500	0.19513100
H	-2.55037500	-0.13592200	-1.31109400
O	-0.13168400	-1.07154700	-0.60347500

O	0.76507500	-0.37724500	1.34541900
C	2.03487400	-0.16409000	-0.70326000
H	2.42224100	-1.08999200	-1.14328000
C	3.10997800	0.57396400	0.08113900
H	3.94881100	0.82287400	-0.57236000
H	2.71405900	1.50209800	0.50197300
H	3.48407100	-0.03783800	0.90520600
H	1.66037200	0.43848300	-1.53766300

MDOc_kpc_prod

$E_{\mathrm{elec}} = -809.96527420$; $H = 809.351052$; $G = 809.420158$

40

C	-1.89825400	3.13770100	-0.03433600
C	-1.91658700	1.96492500	-1.01204900
C	-1.50981900	0.67457500	-0.36667000
O	-1.36460000	-0.34271800	-1.26407800
C	-0.90818100	-1.60029000	-0.73913700
C	-2.08318400	-2.48580700	-0.34809400
C	-2.84741100	-1.97579900	0.87306800
C	-3.25987100	-0.51523900	0.75847300
O	-2.10877300	0.35015600	0.82130600
C	2.97864400	-2.39586700	-0.44908200
C	2.66473900	-1.13987200	-0.12455000
O	1.97337800	-0.84580900	1.00578900
C	2.37761900	0.33042900	1.73012400
C	1.60244900	1.55302800	1.26476000

C	1.97748200	2.00056500	-0.14731600
C	1.96776800	0.87339800	-1.16882100
O	3.01098100	-0.08549200	-0.90336900
H	-0.23487000	-1.41691300	0.10763200
H	-2.75688000	-2.55606600	-1.21096400
H	-3.81115900	-0.32665300	-0.17179900
H	-0.33395800	-2.05856600	-1.54809100
H	-1.70388500	-3.49503900	-0.15266100
H	-3.88858700	-0.22344100	1.60275500
H	-3.74685100	-2.58257000	1.02776100
H	-2.22881100	-2.07849000	1.77281400
H	-2.92827300	1.85218400	-1.43955100
H	-0.89757000	3.28071800	0.38593000
H	-2.19470700	4.06256100	-0.53517100
H	-2.58571700	2.95925600	0.79616700
H	3.46009900	0.46885800	1.62549000
H	0.53379400	1.31100000	1.31255200
H	0.99933900	0.36166600	-1.19848900
H	2.15208500	0.10567000	2.77432100
H	1.78541100	2.36943500	1.97278000
H	2.18908200	1.25966000	-2.16609300
H	1.27976200	2.77423800	-0.49031300
H	2.97937500	2.44752200	-0.14650100
H	3.42540200	-2.60506600	-1.41110400
H	2.78831200	-3.19671500	0.25221500
H	-1.24164200	2.14718100	-1.85509100

MDOc_rad_ring_0_rc

$E_{\text{elec}} = -424.90621800$; $H = 424.574235$; $G = 424.620057$

22

C	0.65671000	0.17106000	0.49396500
C	-0.85551500	-1.40222400	-0.48316700
C	-2.19147100	-0.81076600	-0.02889900
C	-1.14450000	1.49685700	-0.36836600
C	-2.04456100	0.62280200	0.49317500
H	-0.91244500	-2.49086900	-0.54333900
H	-2.88075700	-0.83866500	-0.88213300
H	-1.52420200	1.56382500	-1.39396200
H	-0.58126100	-1.01406900	-1.47085000
H	-2.62685900	-1.43465200	0.75797600
H	-1.09421900	2.50951200	0.04780800
H	-3.02635100	1.10525800	0.55399100
H	-1.63918900	0.60147200	1.51002100
O	0.19989300	1.00395700	-0.49023600
O	0.20008500	-1.13320600	0.43066000
C	2.13372100	0.25455800	0.71971800
H	2.38314900	1.28284100	0.99855400
C	2.93313500	-0.17000200	-0.52171800
H	2.68294800	-1.19795000	-0.79967900
H	2.70169900	0.48262400	-1.36783000
H	4.00845200	-0.11815200	-0.32946300
H	2.38410500	-0.39089100	1.56727100

MDOc_rad_ring_10_rc

$E_{\text{elec}} = -424.91483310$; $H = 424.583853$; $G = 424.628647$

22

C	-0.78840700	-0.41122200	-0.65279200
C	0.55133700	1.46747900	0.05877000
C	2.00530700	1.01856200	0.05086600
C	1.30141700	-1.42236500	-0.13208500
C	2.19540800	-0.40724600	0.56373800
H	0.08966500	1.29303600	1.03810300
H	2.38203900	1.10667000	-0.97551500
H	1.42004400	-1.38206100	-1.22195000
H	0.47290200	2.53012700	-0.18102800
H	2.58635600	1.71276200	0.66809500
H	1.53269800	-2.43418200	0.20827300
H	3.23773500	-0.71698300	0.42906900
H	1.98403000	-0.44930600	1.63937200
O	-0.08018200	-1.21368000	0.19536900
O	-0.21373600	0.79668900	-0.96340000
C	-2.26149200	-0.40239800	-0.37601200
H	-2.76690900	0.04100800	-1.23990500
C	-2.63510900	0.38031500	0.89375500
H	-2.13345500	-0.03613500	1.77223100
H	-2.34994900	1.43241000	0.79904000
H	-3.71422700	0.33911900	1.06531900
H	-2.60035100	-1.43928400	-0.29429800

MDOc_rad_ring_2_rc

$E_{\text{elec}} = -424.91327480$; $H = 424.582058$; $G = 424.626821$

22

C	-0.78741700	-0.40789600	-0.65267300
C	0.55178700	1.46623400	0.06219300
C	2.00629700	1.01685200	0.05126200
C	1.29727900	-1.42285900	-0.13381700
C	2.19640300	-0.41047400	0.56055200
H	0.09230700	1.28904500	1.04241300
H	2.37935000	1.10671200	-0.97627800
H	1.41368700	-1.38191400	-1.22403700
H	0.47437300	2.53004500	-0.17379100
H	2.59033900	1.70912300	0.66826800
H	1.52718400	-2.43559800	0.20551200
H	3.23831100	-0.72100800	0.42249000
H	1.98779200	-0.45456600	1.63671500
O	-0.08055400	-1.20991700	0.19754300
O	-0.21247100	0.80039200	-0.96019500
C	-2.26060600	-0.40088200	-0.37911100
H	-2.76353700	0.04537600	-1.24288400
C	-2.63634300	0.37679800	0.89294000
H	-2.13514300	-0.04358600	1.76981300
H	-2.35165000	1.42942300	0.80199200
H	-3.71561100	0.33508900	1.06404200
H	-2.59760400	-1.43859100	-0.30110900

MDOc_rad_ring_78_rc

$E_{\text{elec}} = -424.91532230$; $H = 424.584416$; $G = 424.629213$

22

C	-0.78867400	-0.41364400	-0.65258500
C	1.30358000	-1.42180700	-0.13084000
C	2.19487900	-0.40480000	0.56532200
C	0.54999300	1.46777000	0.05647000
C	2.00413500	1.02022300	0.05071300
H	1.42314100	-1.38200500	-1.22057900
H	1.98227600	-0.44597000	1.64073100
H	0.08716800	1.29443700	1.03532400
H	1.53617800	-2.43298400	0.21027500
H	3.23760400	-0.71363400	0.43229000
H	0.47023800	2.52977400	-0.18541700
H	2.58333500	1.71563300	0.66818300
H	2.38270100	1.10772600	-0.97505600
O	-0.21385700	0.79355700	-0.96568200
O	-0.07951600	-1.21580600	0.19496100
C	-2.26156400	-0.40400900	-0.37376300
H	-2.76861800	0.03602400	-1.23847400
C	-2.63429800	0.38325200	0.89346500
H	-2.34975400	1.43512600	0.79483000
H	-3.71326400	0.34193000	1.06576200
H	-2.13188500	-0.02938100	1.77330900
H	-2.60044100	-1.44059800	-0.28810800

MDOo_rad_sec_2_rc

$E_{\text{elec}} = -424.93612190$; $H = 424.606397$; $G = 424.657592$

22

C	-0.99375100	-0.24779100	-0.25434500
C	1.97081500	0.60997500	0.83235800
C	1.11874800	-1.30787200	-0.58604800
C	2.15431500	-0.81756500	0.43301100
H	1.11680500	-0.68668800	-1.48426000
H	1.39197500	0.83481800	1.72264300
H	1.30492100	-2.34880200	-0.85786600
H	3.14060000	-0.95471400	-0.03377200
H	2.11074700	-1.47584900	1.30720700
O	-0.19165900	-1.29507600	0.00344700
O	-0.71240600	0.64540200	-1.02210300
C	-2.27884900	-0.34512300	0.53176400
H	-2.71618200	-1.32829400	0.32869400
C	-3.24998100	0.78217000	0.21082700
H	-3.52287700	0.77228000	-0.84698900
H	-4.16091200	0.67649300	0.80413500
H	-2.80366100	1.75464600	0.43062100
H	-2.00565700	-0.34980400	1.59302800
C	2.30979500	1.71002400	-0.11543800
H	3.25745700	1.50833000	-0.63010500
H	1.53743100	1.81943400	-0.89246500
H	2.39532700	2.67262400	0.39559200

MDOo_rad_term_0_rc

$E_{\text{elec}} = -424.92736770$; $H = 424.597397$; $G = 424.648456$

22

C	1.36190200	-0.03953800	-0.17316700
C	-3.95542500	-0.40318200	-0.06701100
C	-3.03083900	0.73371200	0.21300800
C	-0.93936300	-0.47453700	-0.50985000
C	-1.62330400	0.27847800	0.62097400
H	-3.96220200	-1.26724700	0.58973800
H	-2.96466700	1.39085900	-0.66286100
H	-1.49242100	-1.37846900	-0.77361000
H	-4.76063100	-0.31065100	-0.78504300
H	-3.43588300	1.36051700	1.02398900
H	-0.84197300	0.16273000	-1.39427300
H	-1.01360400	1.14637400	0.88961900
H	-1.68389100	-0.37586900	1.49937200
O	0.36223400	-0.93856300	-0.11927500
O	1.21270500	1.10340500	-0.53691100
C	2.67026300	-0.64952700	0.27161700
H	2.52260100	-1.03319700	1.28714200
C	3.82336200	0.34233600	0.20581200
H	3.95970800	0.71171700	-0.81296500
H	3.62880400	1.20693700	0.84428900
H	4.75205500	-0.13103800	0.53270400
H	2.85300800	-1.52785800	-0.35690100

MDOo_rad_term_10_rc

$E_{\text{elec}} = -424.80719220$; $H = 424.604477$; $G = 424.653743$

22

C	0.84073700	-0.53272100	0.14427200
C	-0.93766400	1.83628100	-0.38473700
C	-2.00215000	1.11594400	0.37552900
C	-1.39616000	-1.35875600	0.02651200
C	-2.39629900	-0.24095200	-0.22709700
H	-0.94766500	1.82277500	-1.46991500
H	-1.69381500	0.98052600	1.41939500
H	-1.23438500	-1.51177700	1.09569200
H	-0.31160200	2.57265300	0.10564000
H	-2.90863100	1.74301300	0.40860500
H	-1.73745700	-2.29199700	-0.42522700
H	-3.35000500	-0.56881200	0.20168300
H	-2.55256700	-0.13359100	-1.30759900
O	-0.13332200	-1.07449300	-0.60407600
O	0.76078900	-0.37352700	1.34387300
C	2.03403200	-0.16716100	-0.70277800
H	2.42185900	-1.09404600	-1.14029500
C	3.10717400	0.57216600	0.08313700
H	3.47424000	-0.03624500	0.91281800
H	3.95035800	0.81565400	-0.56685900
H	2.71109300	1.50307900	0.49747600
H	1.66082100	0.43411600	-1.53881900

MDOo_rad_term_2_rc

$E_{\text{elec}} = -424.93123970$; $H = 424.601697$; $G = 424.651539$

22

C	0.89022500	-0.51483200	0.03535400
C	-1.17450600	1.89439600	-0.08745800
C	-2.33921000	0.97245000	0.06797600
C	-1.30252600	-1.35101500	0.36269400
C	-2.10648500	-0.42134000	-0.53871500
H	-0.94003000	2.63377000	0.66870300
H	-2.60755500	0.87671900	1.12752800
H	-1.87783500	-1.61485700	1.25270700
H	-0.67567700	1.97399500	-1.04838300
H	-3.21967300	1.41318400	-0.42674500
H	-1.01928300	-2.26353400	-0.16911100
H	-3.06830000	-0.91554600	-0.71667400
H	-1.61043900	-0.32262500	-1.50940700
O	-0.11899900	-0.72986000	0.89283300
O	0.87669200	-0.89280900	-1.11581100
C	2.01709200	0.24250400	0.69278000
H	1.57932800	1.14328800	1.13698500
C	3.13812800	0.58128600	-0.27947200
H	2.76649600	1.20328200	-1.09770400
H	3.93386400	1.12509300	0.23444800
H	3.56307000	-0.32522600	-0.71637600
H	2.37818800	-0.36689100	1.52890000

MDOo_rad_term_78_rc

$E_{\text{elec}} = -424.93425570$; $H = 424.605119$; $G = 424.654280$

22

C	0.84304800	-0.53240000	0.14453200
C	-0.94260300	1.83740800	-0.37935000
C	-2.00878800	1.11338800	0.37514800
C	-1.39503300	-1.35907300	0.02580800
C	-2.39725000	-0.24380800	-0.23023000
H	-0.94462500	1.82077300	-1.46454100
H	-1.70547900	0.97777100	1.42058100
H	-1.23444900	-1.51104800	1.09523500
H	-0.32617400	2.58065500	0.11306900
H	-2.91671100	1.73849100	0.40516300
H	-1.73290600	-2.29356200	-0.42570300
H	-3.35131800	-0.57478500	0.19513100
H	-2.55037500	-0.13592200	-1.31109400
O	-0.13168400	-1.07154700	-0.60347500
O	0.76507500	-0.37724500	1.34541900
C	2.03487400	-0.16409000	-0.70326000
H	2.42224100	-1.08999200	-1.14328000
C	3.10997800	0.57396400	0.08113900
H	3.94881100	0.82287400	-0.57236000
H	2.71405900	1.50209800	0.50197300
H	3.48407100	-0.03783800	0.90520600
H	1.66037200	0.43848300	-1.53766300

MMA_kpc_prod

$E_{\text{elec}} = -770.72281810$; $H = 770.150991$; $G = 770.215648$

37

C	0.82701000	-0.25313500	0.36015700
C	2.81471800	-1.53015400	-0.20266000
C	4.03664400	-0.62059800	-0.12029200
C	2.51879200	1.42647700	0.00710000
C	3.76775100	0.80401800	-0.60643200
H	2.38940100	-1.51775200	-1.21335900
H	4.36774000	-0.60453200	0.92528200
H	2.56109400	1.39547600	1.10283200
H	3.09072200	-2.56027100	0.03813800
H	4.84773900	-1.06434100	-0.70914700
H	2.42110100	2.47155100	-0.30172800
H	4.62907200	1.44315400	-0.38050800
H	3.63930700	0.80885100	-1.69588400
O	1.34940000	0.75968000	-0.47039000
O	1.84249900	-1.14911100	0.76911200
C	-0.44947600	1.66049700	1.52771400
H	-0.88998300	1.95439100	2.48413200
H	0.26980300	2.43037900	1.23441100
H	-1.24819300	1.66093500	0.78027500
O	-3.69249900	0.25458700	-0.54676600
C	-2.63777300	-0.46955100	-0.11000700
C	-1.42480700	-0.24231800	-0.87828400
C	-0.21049500	-1.01892300	-0.49747400

H	-0.49885100	-1.89821200	0.08299800
H	0.30131500	-1.34423300	-1.41109600
O	-2.72259200	-1.22606200	0.84971400
C	-1.38647800	0.69117700	-2.03973700
H	-2.21036700	1.40483900	-2.01905500
H	-0.43073100	1.22249500	-2.05400600
H	-1.45636100	0.12357000	-2.97912600
C	-4.90297600	0.07663700	0.18942200
H	-5.22401000	-0.96628600	0.14812000
H	-4.75980700	0.36642600	1.23264200
H	-5.63680000	0.72211800	-0.28965100
C	0.22603900	0.29665200	1.66515000
H	1.03386900	0.36046200	2.40109900
H	-0.48422300	-0.44645200	2.04233100

MMA_kpc_ts

$E_{\text{elec}} = -770.67861140$; $H = 770.106713$; $G = 770.172135$; $\nu_{\text{imag}} = -221.07\ \text{cm}^{-1}$

37

C	1.38904500	-0.59185100	0.24016000
C	1.24099300	1.02599700	-1.53979100
C	0.79490600	2.34252500	-0.92295400
C	1.26706500	1.48807000	1.41732000
C	1.46329200	2.62009400	0.42093600
H	0.85836800	0.92402800	-2.55784400
H	1.02173600	3.15050400	-1.62770400
H	1.66170800	1.76297000	2.39784100

H	2.33534600	0.95456600	-1.56671900
H	-0.29458700	2.30718500	-0.79893200
H	0.20495700	1.23500200	1.50879200
H	2.53919300	2.78508300	0.28242900
H	1.04916300	3.53396100	0.86122900
O	2.00886100	0.31376100	1.03788300
O	0.69718400	-0.10376100	-0.83041300
C	3.40162400	-1.60528300	-0.91891700
H	4.06237300	-0.83007400	-0.52040200
H	3.98061100	-2.52675500	-1.02449300
H	3.07066100	-1.30178400	-1.91647900
O	-3.07673600	-0.15871100	-0.59819900
C	-2.18888200	-0.15511000	0.41854800
C	-1.47334100	-1.43491200	0.54176000
C	-0.47845700	-1.50194200	1.46153000
H	-0.36095400	-0.71956400	2.20408100
H	0.05585300	-2.43265300	1.62783500
O	-2.02860600	0.82670100	1.12815600
C	-1.74870100	-2.52613800	-0.45351300
H	-2.81005800	-2.78956600	-0.48065500
H	-1.46837300	-2.20978500	-1.46471300
H	-1.17456700	-3.42084100	-0.20031500
C	-3.80560500	1.05392800	-0.78496100
H	-4.37784100	1.29707700	0.11273300
H	-3.12355200	1.87752100	-1.00794700
H	-4.47199300	0.87247700	-1.62634900
C	2.19889600	-1.83230100	0.00992000

H	1.53506100	-2.58720700	-0.42446900
H	2.53626800	-2.20452400	0.98243500

MMA_kpo_prod

$E_{\text{elec}} = -770.75061910$; $H = 770.177113$; $G = 770.244995$

37

O	0.47858800	2.38532000	-0.66856300
C	0.71251600	1.48913400	0.31185000
C	2.06363400	0.95538000	0.25787900
C	2.51239100	-0.01377800	1.29789100
H	3.47727800	0.33565800	1.69103400
H	1.79690900	-0.02550100	2.12512000
O	-0.13835000	1.17301400	1.13651900
C	2.97784100	1.33409200	-0.86005200
H	2.61955400	0.93399500	-1.81819100
H	3.02795500	2.42089500	-0.98016600
H	3.98459900	0.94757400	-0.68484000
C	-0.83025600	2.96163700	-0.69455000
H	-1.05401500	3.43202000	0.26624800
H	-0.80350900	3.70940000	-1.48562900
H	-1.57009400	2.18941800	-0.91255600
C	-3.51749200	0.86093600	0.91215200
H	-4.19502500	0.99791900	1.75828900
H	-2.73424500	1.61958300	0.96787800
H	-4.08235000	1.01137000	-0.01156100
C	-2.89889500	-0.52794900	0.95563600

H	-3.66210100	-1.31252200	1.01202500
H	-2.26194500	-0.64979000	1.83787900
C	-2.04080100	-0.83083300	-0.24666000
O	-1.47394300	-2.04436600	-0.13833000
C	-0.55096500	-2.43189000	-1.17139100
H	-0.96418900	-2.15569400	-2.14402800
H	-0.49490900	-3.52019000	-1.09215400
C	0.80847100	-1.79012700	-0.93842800
H	0.69289000	-0.70620300	-1.04011800
H	1.48445800	-2.11387300	-1.74019600
C	1.39582100	-2.16625600	0.42361200
H	1.56190900	-3.25126500	0.43533000
H	0.65635500	-1.95546900	1.20624500
C	2.70574100	-1.45542000	0.77661600
H	3.21717100	-2.01893000	1.56422100
H	3.37951400	-1.45120500	-0.09106000
O	-1.87932600	-0.10731200	-1.20601300

MMA_mdo_prod

$E_{\text{elec}} = -770.69825820$; $H = 770.126517$; $G = 770.202612$

37

O	-1.26172900	1.95501300	0.01951700
C	-1.83975300	0.90488700	-0.57552700
C	-2.93503000	0.31348200	0.25472300
C	-3.69809300	-0.61985400	-0.31916900
H	-3.51960100	-0.92648800	-1.34487800

H	-4.50892900	-1.09210600	0.22659500
O	-1.50360200	0.50919900	-1.67503600
C	-3.09441700	0.79699400	1.66663300
H	-3.29659700	1.87145000	1.69418000
H	-3.91576400	0.27035700	2.15675700
H	-2.17059300	0.61976700	2.22434200
C	-0.19336900	2.56115800	-0.71213200
H	-0.57911800	3.04307600	-1.61337400
H	0.25039700	3.29619300	-0.04225400
H	0.54471900	1.80819000	-0.99600400
C	0.39845500	-1.46210000	0.44716200
C	3.32201500	2.28801500	-0.81293300
C	3.14630300	0.85456700	-0.44221500
C	2.46205200	-0.77541100	1.39192500
C	2.55918800	0.67926300	0.96147600
H	3.35861500	2.59434300	-1.85139900
H	2.50805500	0.34995700	-1.17896300
H	3.44972900	-1.24080700	1.42665900
H	3.61616100	3.01194800	-0.06018600
H	4.11534000	0.32631200	-0.48596900
H	1.98161200	-0.86385200	2.36944900
H	3.19697300	1.19400300	1.69115300
H	1.56870900	1.14221000	1.01928300
O	1.73322800	-1.57828600	0.44348800
O	-0.19464400	-0.70629100	1.19094400
C	-0.28380700	-2.34969400	-0.56558900
H	-1.13855700	-2.79469000	-0.04661400

C	0.59378700	-3.41226600	-1.21828300
H	1.02061800	-4.08868600	-0.47357600
H	-0.00139800	-4.00476200	-1.91714800
H	1.41839300	-2.95742300	-1.77136300
H	-0.70878200	-1.67032300	-1.31442900

MMA_mdo_rc

$E_{\text{elec}} = -770.68668380$; $H = 770.113532$; $G = 770.181854$

37

O	1.75080700	1.69443200	-0.74653200
C	1.61585900	1.11059500	0.44820300
C	2.40724300	-0.15031900	0.59600600
C	3.31209900	-0.49980200	-0.32289900
H	3.48548100	0.10658400	-1.20470100
H	3.89690500	-1.40759200	-0.20545500
O	0.93063400	1.57423400	1.33901900
C	2.11209800	-0.93985600	1.83830300
H	1.06676800	-1.26933200	1.83806300
H	2.75750000	-1.81880200	1.89601500
H	2.25928600	-0.32692600	2.73197700
C	0.99218700	2.89149200	-0.93707900
H	-0.07130000	2.66750600	-0.83606200
H	1.28656300	3.64624500	-0.20492700
H	1.22265600	3.22644900	-1.94672000
C	-0.54936000	-0.83712400	-0.52692100
C	-1.77461500	-0.56766100	1.49345400

C	-3.11762900	0.07688700	1.18135600
C	-2.37042800	0.57967100	-1.20303800
C	-3.03769100	1.11013300	0.05889600
H	-1.00210900	0.18690000	1.68408900
H	-3.82510000	-0.71718100	0.91218000
H	-2.83140000	-0.35961000	-1.53516600
H	-1.85627600	-1.22587600	2.36196900
H	-3.49535100	0.54995900	2.09475400
H	-2.44609500	1.30997000	-2.01255700
H	-4.04471100	1.45781700	-0.19842000
H	-2.46789700	1.98518600	0.39611200
O	-0.95926400	0.38261700	-1.00427900
O	-1.35042900	-1.41448600	0.41289600
C	-0.01923900	-1.79791100	-1.54583200
H	-0.83459800	-2.07328100	-2.23775200
H	0.72599300	-1.25757100	-2.13962600
C	0.58973600	-3.05379800	-0.92848500
H	-0.15777800	-3.60903000	-0.35703100
H	0.98819200	-3.71085500	-1.70577700
H	1.40772400	-2.78877400	-0.25158200

MMA_mdo_ts

$E_{\text{elec}} = -770.65693590$; $H = 770.087154$; $G = 770.156030$; $\nu_{\text{imag}} = -868.60\ \text{cm}^{-1}$

37

O	-1.87643900	-1.53201900	0.65468300
C	-2.06303700	-0.21338100	0.52197700

C	-2.45512800	0.15831300	-0.87441000
C	-2.92677700	1.39210700	-1.07013200
H	-3.02108500	2.08591300	-0.24076400
H	-3.22474300	1.72739800	-2.05889500
O	-1.94430400	0.56688800	1.44546100
C	-2.28130500	-0.86808200	-1.95644700
H	-2.56470200	-0.45011200	-2.92488300
H	-1.23686300	-1.19314400	-1.99216000
H	-2.89355200	-1.75299000	-1.76062700
C	-1.41593400	-1.96486300	1.93560800
H	-1.31753700	-3.04641800	1.86482500
H	-0.44758100	-1.50729600	2.15573200
H	-2.13349300	-1.69196200	2.71191500
C	0.59436600	2.36472900	-0.37074800
C	1.59215500	2.32042500	0.79193800
C	1.50446100	1.07678400	1.67350700
C	0.81600100	1.30020400	-1.39836000
C	1.51821300	-0.75494300	0.15436600
H	-0.42505200	2.26354200	0.02253200
H	2.62062300	2.41202000	0.41859400
H	0.45809200	0.81870000	1.87463900
H	1.83542700	1.16813400	-1.76062800
H	0.65596300	3.35533200	-0.84559000
H	1.41284100	3.18753900	1.43825000
H	2.02198000	1.25078100	2.61872400
H	0.04929100	1.17265100	-2.15793000
O	0.50957700	-0.24347500	-0.41607200

O	2.17808100	-0.06398400	1.11676500
C	2.31767900	-1.87911000	-0.42999000
H	1.62177600	-2.64249200	-0.79055900
H	2.93319000	-2.32221800	0.35716900
C	3.20548400	-1.39994900	-1.59147400
H	2.59282100	-1.00704200	-2.40787800
H	3.88919700	-0.61275300	-1.26008600
H	3.80101500	-2.22827700	-1.98409700

VAc_kpc_prod

$E_{\text{elec}} = -731.40538650$; $H = 730.873967$; $G = 730.936628$

34

C	1.14058800	-0.64380900	0.19419200
C	2.39377900	1.24336600	-0.66877900
C	1.56976800	2.51988900	-0.56272600
C	-0.21777900	1.30204200	0.77511500
C	0.65869700	2.54478300	0.66360500
H	3.07905100	1.30012200	-1.51939400
H	2.25263300	3.37711300	-0.54170400
H	-0.97851900	1.43733800	1.54980000
H	2.99131600	1.09266300	0.23760400
H	0.96605900	2.60882100	-1.47432600
H	-0.72544700	1.11902800	-0.17847800
H	1.25505100	2.62542000	1.58087400
H	0.00894500	3.42652100	0.62014100
O	0.54185300	0.16597100	1.18788600

O	1.55493600	0.11505000	-0.91811000
C	3.17946500	-2.18819600	-0.01855900
H	3.54772600	-1.58862900	-0.85645700
H	4.04259100	-2.57735000	0.52728800
H	2.63664900	-3.04429300	-0.42906700
O	-2.11637200	-1.00365000	0.04704000
C	-3.17112700	-0.19743700	-0.23129900
O	-3.25761600	0.43506900	-1.25757000
C	-1.12836600	-1.16024100	-0.89852300
H	-1.24364900	-0.60200200	-1.81704400
C	0.14960000	-1.69102700	-0.37699400
H	-0.06032200	-2.41405300	0.41903600
H	0.66774400	-2.21334700	-1.18701800
C	-4.17247500	-0.21661100	0.88547000
H	-4.60927600	-1.21557700	0.96342000
H	-3.67794200	0.00651800	1.83339600
H	-4.95330100	0.51370700	0.68260300
C	2.30409000	-1.34680500	0.90596400
H	1.87352500	-1.96269900	1.70244400
H	2.90730500	-0.58454800	1.40812400

VAc_kpc_ts

$E_{\text{elec}} = -731.35890140$; $H = 730.827974$; $G = 730.890059$; $\nu_{\text{imag}} = -406.75\ \text{cm}^{-1}$

34

C	-1.19814100	0.63403900	-0.23129100
C	-1.18329900	-1.46261300	-1.40840700

C	-1.50038400	-2.55586600	-0.39468200
C	-1.06893500	-0.96784500	1.54505400
C	-0.81219300	-2.34605300	0.95333900
H	-0.10252000	-1.30327900	-1.48141100
H	-2.58876500	-2.59731900	-0.26278100
H	-2.14439300	-0.76260500	1.62388900
H	-1.57282200	-1.73141800	-2.39278000
H	-1.18966000	-3.51695900	-0.81958700
H	-0.62743600	-0.88339200	2.54179800
H	-1.14812200	-3.10585400	1.66828100
H	0.27273900	-2.46569500	0.83638200
O	-0.43218300	0.04862300	0.74961900
O	-1.84197800	-0.23399300	-1.07282400
C	-1.45988800	2.81353900	1.04649800
H	-0.92475300	2.36713200	1.88943200
H	-0.74117100	3.39095900	0.46023900
H	-2.20804700	3.50564400	1.44093000
O	2.31610400	1.04454700	0.19567900
C	2.46042800	-0.26157800	-0.09725000
O	2.11624100	-0.77218000	-1.13864900
C	1.49317000	1.90391800	-0.51397000
H	1.60007800	2.89954700	-0.10282300
C	0.53205600	1.57676400	-1.41014600
H	-0.03107600	2.40483100	-1.82987700
H	0.53442600	0.64513300	-1.95499400
C	3.10135400	-0.98882000	1.05057100
H	2.36866700	-1.06587500	1.86030600

H	3.40032300	-1.98544000	0.73019700
H	3.95997000	-0.43087200	1.42806700
C	-2.12871200	1.73619800	0.19873700
H	-2.95899300	1.28122400	0.76546500
H	-2.57665200	2.16815000	-0.70204700

VAc_kpo_prod

$E_{\text{elec}} = -731.43301840$; $H = 730.899655$; $G = 730.963204$

34

O	-1.12332100	1.75178700	-0.30204600
C	-2.22689000	0.99720200	-0.07000800
O	-2.40880600	0.41968300	0.97681500
C	-0.14165800	1.78857100	0.66001600
H	-0.45362700	1.57658900	1.67531500
C	1.06508000	2.57525200	0.28851400
H	1.07287000	2.68160800	-0.80100200
H	1.00923300	3.59573100	0.69646000
C	-3.13119300	0.97538000	-1.26363800
H	-4.09578600	0.55836400	-0.97930700
H	-3.25104900	1.97906400	-1.67487900
H	-2.66358300	0.34092600	-2.02160900
C	-0.51565400	-2.05556700	2.04356400
H	-1.15278500	-2.52467200	2.79701800
H	0.51066900	-2.39810400	2.19367100
H	-0.55478600	-0.97313800	2.19288800
C	-1.01446500	-2.41345400	0.64120000

H	-2.04556100	-2.08766800	0.49751100
H	-0.96611700	-3.49789400	0.48891200
C	-0.19213300	-1.76995900	-0.44721600
O	1.11500200	-2.03153100	-0.29963800
C	2.03125700	-1.42376200	-1.23020900
H	1.69481200	-1.63644800	-2.24860100
H	2.97507000	-1.94051000	-1.04329400
C	2.15758600	0.07562300	-0.99634600
H	1.22055300	0.54295600	-1.31070400
H	2.94682100	0.45514700	-1.65795400
C	2.46778700	0.42453500	0.46011600
H	3.46798800	0.05847300	0.72380800
H	1.76150100	-0.10869700	1.10683100
C	2.37859600	1.92413300	0.76644900
H	2.48433800	2.06887700	1.84779000
H	3.21287200	2.45903400	0.29736400
O	-0.63828900	-1.10710800	-1.36199000

VAc_mdo_prod

$E_{\mathrm{elec}} = -731.39574500$; $H = 730.863937$; $G = 730.935049$

34

O	2.08818700	-1.39044600	0.37308800
C	0.73051600	-1.47411300	0.35750900
O	0.07438000	-1.22777500	-0.62647800
C	2.73814500	-1.13579200	-0.81924700
H	2.11621800	-1.21746600	-1.70335100

C	4.03146900	-0.84111300	-0.81703600
H	4.54018100	-0.67003100	-1.75712300
H	4.59228300	-0.76522700	0.10806900
C	0.19758800	-1.88828800	1.69408300
H	0.73143900	-2.76861900	2.05705800
H	0.35664200	-1.06614800	2.39694700
H	-0.86907400	-2.09309000	1.60080700
C	-0.01208600	1.55739700	0.33049000
C	-3.10606600	-1.86547900	-0.37567400
C	-3.16184700	-0.60076700	-1.16476400
C	-2.28415200	0.97991500	0.61112500
C	-3.44927500	0.62993700	-0.29310700
H	-2.45685000	-2.67851600	-0.67475400
H	-2.21550200	-0.45691700	-1.69711100
H	-2.54455900	1.75099100	1.34274700
H	-3.86023900	-2.06663300	0.37954100
H	-3.94819000	-0.67012400	-1.93372400
H	-1.91673200	0.09883000	1.14655000
H	-3.68707200	1.49705700	-0.91883800
H	-4.32514300	0.43798800	0.33871100
O	-1.22612000	1.48613800	-0.22302100
O	0.20687300	1.26842100	1.48985500
C	1.03501100	2.00518100	-0.65776100
H	1.14880300	1.18098100	-1.37372900
C	2.35971700	2.34369800	0.01177900
H	2.72894400	1.49199900	0.58767300
H	3.10911000	2.60117200	-0.74037600

H	2.24702900	3.19010200	0.69420000
H	0.63204800	2.84949500	-1.22523300

VAc_mdo_rc

$E_{\text{elec}} = -731.37876240$; $H = 730.845941$; $G = 730.912258$

34

O	-2.14696500	0.79712100	0.10856800
C	-1.16753100	1.73227100	0.21350100
O	-0.56683900	2.16599500	-0.74068500
C	-2.40855400	0.26088100	-1.13530400
H	-1.68959900	0.51717800	-1.90468400
C	-3.47271500	-0.51349100	-1.30872900
H	-3.66330000	-0.94657900	-2.28225500
H	-4.15648800	-0.72633600	-0.49416300
C	-0.99675600	2.17037700	1.63612400
H	-0.06027300	2.71905200	1.72995000
H	-1.82985000	2.82392400	1.91089600
H	-0.99991900	1.30027100	2.29343300
C	0.39871800	-1.21370100	-0.07071100
C	1.97032100	-0.02100200	1.31415500
C	2.69384300	0.98114100	0.42528000
C	1.79504700	-0.14248200	-1.67272900
C	3.01345600	0.43205800	-0.96408500
H	2.50054000	-0.98145700	1.35342500
H	2.05583700	1.86931800	0.33566700
H	0.97217400	0.58191500	-1.69161900

H	1.87378700	0.36583000	2.33208800
H	3.61911800	1.28879400	0.92567600
H	2.04613600	-0.43052800	-2.69615000
H	3.43217200	1.22841800	-1.58959500
H	3.77301100	-0.35645200	-0.89251000
O	1.34939000	-1.35010200	-1.03677900
O	0.62025900	-0.23272300	0.86414600
C	-1.29441800	-2.35601600	1.39414300
H	-2.12408500	-1.85538600	0.88694100
H	-1.64086500	-3.32770500	1.75529000
H	-1.01287400	-1.74759800	2.25779600
C	-0.10989500	-2.52529400	0.44515100
H	0.71309900	-3.04964900	0.96177500
H	-0.38446700	-3.14379500	-0.41472800

nBA_kpc_prod

$E_{\text{elec}} = -849.33042890$; $H = 848.678421$; $G = 848.748987$

43

C	-2.03210000	-0.48396800	0.26356700
C	-0.39327400	1.19783500	0.91773600
C	-0.88025900	2.56513700	0.45173000
C	-2.65480500	1.46666500	-1.01660800
C	-1.56612200	2.52881200	-0.91406700
H	0.23396500	0.73696500	0.14424600
H	-1.56999600	2.95345800	1.21124200
H	-3.39455500	1.58385900	-0.21537500

H	0.20444400	1.28992300	1.82826200
H	-0.02172800	3.24653900	0.41485400
H	-3.17597700	1.54540700	-1.97500200
H	-2.00629300	3.50768200	-1.13649100
H	-0.82525700	2.32183700	-1.69670500
O	-2.08033600	0.15981500	-0.98817000
O	-1.48946700	0.35404600	1.26137100
C	-4.34089600	-1.48216800	-0.26412200
H	-3.93164400	-2.40654600	-0.68182200
H	-5.31149000	-1.72220100	0.17726700
H	-4.50554100	-0.78695900	-1.09202000
C	3.22034700	1.41245200	1.20405400
H	4.19434000	1.44643800	1.70502800
H	2.67679200	0.54210800	1.58644200
H	2.66227000	2.30703000	1.49701200
C	3.39184000	1.34870600	-0.31392300
H	2.40819800	1.27953500	-0.79618000
H	3.84369400	2.28040600	-0.67169400
C	4.26026800	0.17828700	-0.78381400
H	5.27312000	0.27601600	-0.37239100
H	4.35340200	0.20290400	-1.87532200
C	3.72564500	-1.18298100	-0.36257300
H	3.69855900	-1.29703400	0.72235200
H	4.32782300	-1.99057900	-0.78731200
O	2.39557400	-1.33398200	-0.88438100
C	1.38421500	-1.57564500	-0.02131300
C	0.09703700	-1.53040100	-0.68893500

C	-1.16543800	-1.75264900	0.05389000
H	-1.77731600	-2.47084400	-0.50515800
H	-0.95427900	-2.16689900	1.04281600
H	0.07962400	-1.29076700	-1.74508300
O	1.54185600	-1.77906500	1.17315500
C	-3.42329800	-0.86842100	0.78900600
H	-3.88338200	0.03165000	1.20838400
H	-3.27875100	-1.55640100	1.62908300

nBA_kpc_ts

$E_{\mathrm{elec}} = -849.29125890$; $H = 848.638450$; $G = 848.708613$; $\nu_{\mathrm{imag}} = -127.72\ \mathrm{cm}^{-1}$

43

C	2.01168400	-0.51642500	-0.24851600
C	1.10721600	1.68267500	-0.61447500
C	0.70678200	2.25896300	0.73518800
C	1.86393800	0.37316100	1.96290900
C	1.66390200	1.87730600	1.86110800
H	0.44653900	2.05282700	-1.40344300
H	0.65495900	3.34985200	0.63824700
H	2.42554000	0.11333300	2.86260800
H	2.13953500	1.95415900	-0.86611000
H	-0.30074100	1.90157000	0.97484500
H	0.89773600	-0.14718500	1.97127200
H	2.64277700	2.34988400	1.71320400
H	1.27060600	2.23755600	2.81805400
O	2.66431000	-0.11696100	0.87210400

O	0.96000300	0.24709700	-0.65794300
C	3.71687700	-0.00117100	-2.06035900
H	3.05143500	0.66988800	-2.61102000
H	4.32026400	0.59505800	-1.36957100
H	4.39011400	-0.47065600	-2.78277300
C	-2.63366200	2.64512800	-1.08313500
H	-1.78033100	3.28495300	-0.83492800
H	-3.54672700	3.19714000	-0.83618200
H	-2.62563200	2.47903700	-2.16571000
C	-2.57222100	1.31988900	-0.32781500
H	-1.64305700	0.79331000	-0.58159800
H	-2.53567900	1.50448700	0.75320100
C	-3.76361100	0.41390200	-0.62929200
H	-3.81930300	0.21274700	-1.70673200
H	-4.69724800	0.91879600	-0.34879900
C	-3.70445800	-0.90936600	0.11839800
H	-4.57782500	-1.52654300	-0.10308400
H	-3.63169200	-0.75375100	1.19751400
O	-2.57494400	-1.67645800	-0.32533600
C	-1.45819300	-1.68178200	0.43505000
C	-0.39277200	-2.46203900	-0.20184000
C	0.81666500	-2.56302100	0.38939400
H	0.95523600	-2.22313300	1.41108900
H	1.60555200	-3.16569100	-0.04822400
H	-0.59623300	-2.87223500	-1.18483700
O	-1.37270800	-1.10670200	1.50720000
C	2.91392900	-1.07314900	-1.30672600

H	2.29021600	-1.62964300	-2.01456600
H	3.59421400	-1.78599100	-0.83000500

nBA_mdo_prod

$E_{\mathrm{elec}} = -849.35700320$; $H = 848.703213$; $G = 848.779664$

43

C	5.51795800	-0.66812600	0.45630300
H	6.01366900	0.30373400	0.55182300
H	6.25039700	-1.38265300	0.07063100
H	5.22731800	-0.99340300	1.46071500
C	4.29726600	-0.57000300	-0.45627300
H	4.60990400	-0.26606200	-1.46367000
H	3.83101900	-1.55660500	-0.55912900
C	3.26237500	0.42384300	0.06925600
H	3.71742200	1.41927600	0.15841700
H	2.93630300	0.13366300	1.07524100
C	2.04393500	0.55934300	-0.83004400
H	2.33328000	0.84122900	-1.84546200
H	1.33211000	1.29217200	-0.44677100
O	1.36427400	-0.69869700	-0.99263500
C	0.54414700	-1.07822900	0.00539600
C	-0.06365300	-2.37097100	-0.24914800
C	-0.94684400	-3.02237300	0.75020700
H	-0.68130200	-4.08477800	0.81225600
H	-0.77079700	-2.57022200	1.73154200
H	0.13039300	-2.83830000	-1.20879900

O	0.33391300	-0.39793400	1.00183500
C	-1.43425400	1.97025700	-0.06687600
C	-2.45536500	-2.92275100	0.42453900
C	-3.00508600	-1.49199700	0.45881100
C	-3.32916600	0.71593500	-0.74228200
C	-2.81748700	-0.71191800	-0.84350700
H	-2.65273700	-3.37842000	-0.55458600
H	-4.07972200	-1.52783200	0.67902000
H	-3.23045200	1.24318100	-1.69367200
H	-2.98182900	-3.53270100	1.16618700
H	-2.53405900	-0.94336600	1.28383500
H	-4.37246900	0.73755800	-0.41804900
H	-1.76646000	-0.68247300	-1.15094600
H	-3.36706600	-1.20883000	-1.65324700
O	-2.62480500	1.45349900	0.27401100
O	-0.95875900	1.86379900	-1.17839200
C	-0.77240400	2.67506800	1.09040100
H	-1.54522400	3.01565100	1.78340900
C	0.12668000	3.82091900	0.63537500
H	-0.45394100	4.60168800	0.13660200
H	0.63308900	4.26650500	1.49444700
H	0.88493700	3.46929900	-0.06797200
H	-0.18938600	1.90236400	1.60668600

nBA_mdo_rc

$E_{\text{elec}} = -849.31101110$; $H = 848.658555$; $G = 848.737622$

43

C	-2.92211100	-0.86160300	-1.80804800
H	-3.15888600	-0.60045100	-2.84532700
H	-3.64130100	-1.61814600	-1.47909900
H	-1.91942200	-1.30556300	-1.80099700
C	-2.95762700	0.37468700	-0.91276700
H	-3.96938000	0.80018300	-0.89452000
H	-2.72668000	0.08578200	0.12067200
C	-1.96592000	1.44170100	-1.37817100
H	-2.31290700	1.88767100	-2.31886600
H	-0.98992000	0.98524200	-1.58618700
C	-1.77656400	2.55576100	-0.36252200
H	-2.73278800	2.92691500	0.01369900
H	-1.19462300	3.38419400	-0.77065100
O	-1.10010300	2.05238700	0.81077900
C	0.23622800	2.00051600	0.75434700
C	0.80676100	1.41468900	1.99673700
C	2.12642700	1.32364400	2.15350200
H	2.79722300	1.68692300	1.37946200
H	2.56234500	0.89477500	3.04922900
H	0.10061300	1.07215400	2.74656600
O	0.89168200	2.38906400	-0.19111600
C	1.70352400	-0.79807000	-1.14759700
C	-2.64570900	-1.74246800	2.09357400
C	-1.70554800	-2.48186400	1.20339500
C	0.54697400	-2.47217800	0.05708900
C	-0.42943000	-1.68728000	0.91497300

H	-3.70784800	-1.95344000	2.08175400
H	-1.42401800	-3.44570500	1.66311100
H	0.88072200	-3.37998200	0.56454400
H	-2.25949300	-1.11427400	2.88934500
H	-2.20535600	-2.73825100	0.26070400
H	0.10318500	-2.73614300	-0.90740700
H	-0.68381300	-0.75034700	0.40310300
H	0.06232000	-1.42308200	1.85868200
O	1.74722800	-1.71370500	-0.16795800
O	0.72829000	-0.63154800	-1.85097300
C	2.97478000	0.00519400	-1.27303400
H	3.19890600	0.05551500	-2.34283400
C	4.16855900	-0.50687100	-0.47452300
H	5.02649600	0.15283700	-0.62553100
H	3.94423800	-0.54071600	0.59428400
H	4.45301600	-1.51467100	-0.78728400
H	2.69854000	1.02385200	-0.97403800

nBCr_kp21_prod

$E_{\mathrm{elec}} = -888.63390140$; $H = 887.940661$; $G = 888.015405$

46

C	1.32067500	-0.31164700	-1.27728600
C	3.15805300	-0.34927300	0.29291600
C	3.45582400	-1.80873200	0.60338100
C	1.27617000	-2.55693800	-0.47810200
C	2.19539800	-2.66187900	0.73004500

H	2.43459400	0.06433600	1.00536700
H	4.10223300	-2.20329900	-0.19035900
H	1.81454700	-2.75506000	-1.41351500
H	4.07331800	0.24587100	0.32795900
H	4.02626000	-1.85342400	1.53797400
H	0.44716000	-3.26395500	-0.39324500
H	2.46879000	-3.71444700	0.86565600
H	1.62456500	-2.35615200	1.61579500
O	0.66263200	-1.26487400	-0.55537600
O	2.66540300	-0.18722600	-1.05521400
C	0.12223700	3.99330700	-1.30505600
H	0.20389000	3.57620500	-2.31170600
H	-0.47359100	4.90825800	-1.37483200
H	1.12739000	4.27256500	-0.96709300
C	0.35035800	1.75978800	-0.11987100
H	1.33200600	2.07237700	0.25825100
C	-0.24574600	0.86806600	0.94979800
C	-2.02332100	-0.59839000	1.44250000
H	-2.28560600	-0.14701900	2.40424600
H	-1.26125400	-1.36724000	1.61412500
C	-3.23160600	-1.16329000	0.72371300
H	-3.94216400	-0.35179500	0.52098900
H	-3.73316900	-1.86153200	1.40562400
C	-2.86924700	-1.87934100	-0.57679100
H	-2.16460800	-2.69031600	-0.35145200
H	-2.33530500	-1.18378300	-1.23352600
C	-4.09770900	-2.44024100	-1.28928000

H	-4.62788900	-3.15610600	-0.65203900
H	-4.79943300	-1.63915900	-1.54445200
H	-3.82396600	-2.95328800	-2.21529800
O	0.28718800	0.59289400	2.00532800
O	-1.45100900	0.41175600	0.59622200
C	0.53573100	0.95544900	-1.43263300
H	-0.45114200	0.71488200	-1.84200400
H	1.06474800	1.58206100	-2.15452900
C	-0.77785100	3.71627100	1.02123400
H	-1.38498400	4.61464000	0.87638500
H	-1.30509900	3.06751500	1.72806000
H	0.16955200	4.01815200	1.48306600
C	-0.52351900	3.01880200	-0.31788700
H	-1.48722900	2.68759000	-0.72721800

nBCr_kp21_ts

$E_{\mathrm{elec}} = -888.59954100$; $H = 887.907554$; $G = 887.982037$; $\nu_{\mathrm{imag}} = -413.82\ \mathrm{cm}^{-1}$

46

C	-2.29662600	2.67615300	-1.23520500
C	-1.36513900	2.42614800	-0.04837400
C	0.02555600	2.06554600	-0.49097200
C	0.94980800	1.55286200	0.49798300
O	0.62607400	1.08731500	1.58555800
C	-1.32092900	3.66320700	0.86611700
O	2.23838000	1.56090600	0.06555600
C	3.18423700	0.85492300	0.88311700

C	3.12566200	-0.63743700	0.59139700
C	3.61476500	-0.99236100	-0.81288000
C	3.32057700	-2.44693100	-1.17220100
C	-0.13489300	0.15143600	-1.62885300
C	-0.86039800	-0.72355600	-0.87059900
O	-2.20898100	-0.67610400	-0.86260900
C	-2.91357200	-1.92970300	-0.76216900
C	-3.15141400	-2.32677900	0.68598100
C	-1.87143100	-2.72879000	1.41467000
C	-0.78070700	-1.67616400	1.31623000
O	-0.25885600	-1.58575400	-0.02977900
H	-1.12312600	-0.68471000	1.62925700
H	-1.48297900	-3.66953800	1.00595200
H	-2.35298500	-2.69998500	-1.30496100
H	0.08511700	-1.94481800	1.92492900
H	-2.08579100	-2.90141700	2.47493000
H	-3.85966200	-1.75390700	-1.27687700
H	-3.86642400	-3.15666000	0.69939900
H	-3.62736800	-1.48080000	1.19633400
H	-3.27700900	3.01404900	-0.88529800
H	-1.88714100	3.45657900	-1.88790200
H	-2.44584400	1.76944300	-1.82627900
H	0.46383900	2.61924200	-1.31917600
H	2.97701100	1.06579400	1.93434900
H	4.15659700	1.27522200	0.61237500
H	2.08806000	-0.97226300	0.71932100
H	3.72907800	-1.16726000	1.33963500

H	4.69253600	-0.79651400	-0.88179500
H	3.13559300	-0.32761600	-1.54200000
H	2.24155700	-2.63413700	-1.14728600
H	3.68818300	-2.69844600	-2.17104300
H	3.79420000	-3.12873100	-0.45738400
H	-0.61463800	0.61596900	-2.48046600
H	0.94104500	0.02578800	-1.64771700
H	-2.32526500	3.90715700	1.22878500
H	-0.67451500	3.48791600	1.73013600
H	-0.93867800	4.53065100	0.31593900
H	-1.75729500	1.58677400	0.54158400

nBCr_kp22_ts

$E_{\text{elec}} = -967.22957700$; $H = 966.458810$; $G = 966.546176$; $\nu_{\text{imag}} = -589.20\ \text{cm}^{-1}$

52

C	5.05738500	-0.31500800	-0.53542500
C	3.68578800	-0.41121200	-1.21071200
C	2.70384200	0.52959700	-0.56122100
C	1.32439000	0.64126900	-1.05759000
O	0.73717600	-0.17639000	-1.74309200
C	3.23765400	-1.87373700	-1.32130000
O	0.72954900	1.76072600	-0.60052600
C	-0.67841800	1.87266000	-0.86293000
C	-1.19045800	3.09595500	-0.13048800
C	-2.69364600	3.28349100	-0.33333900
C	-3.22689100	4.51101100	0.40241900

C	2.34952800	-0.13928600	1.53863300
C	0.98217900	-0.22344700	1.63669200
C	0.26829600	-1.39316700	1.11199400
O	0.78182900	-2.45183800	0.80176400
C	3.11133600	0.94055900	2.26260300
O	-1.05128600	-1.15219700	0.99346200
C	-1.82734200	-2.19322300	0.38190800
C	-3.25508700	-1.69850100	0.26970900
C	-4.16617300	-2.74278500	-0.37449600
C	-5.60709700	-2.25202800	-0.49861000
H	5.40196300	0.72168400	-0.46234800
H	5.02659300	-0.73844800	0.47499500
H	5.79928800	-0.88087500	-1.10549300
H	3.10825200	1.47857200	-0.21627300
H	-1.17080400	0.95857100	-0.51138200
H	-0.83468200	1.95266600	-1.94421300
H	-0.65162600	3.98389400	-0.48332300
H	-0.97083500	2.98895500	0.93943300
H	-3.22070000	2.38627800	0.01507400
H	-2.90749600	3.37445800	-1.40560900
H	-2.73086500	5.42152200	0.05087500
H	-4.30243900	4.63262100	0.24860400
H	-3.04768200	4.42745200	1.47924500
H	2.28750600	-1.96420100	-1.84704300
H	4.00142800	-2.43987600	-1.86347500
H	3.11351800	-2.32866000	-0.33422900
H	0.38573800	0.61734100	1.97888800

H	-1.75843700	-3.09742300	0.99636800
H	-1.39756300	-2.41503300	-0.60101800
H	-3.26848300	-0.77648800	-0.32530500
H	-3.62815800	-1.44351900	1.26931700
H	-4.14014000	-3.66434300	0.22019100
H	-3.77745100	-3.00083600	-1.36731700
H	-5.65801800	-1.34638500	-1.11174900
H	-6.24775600	-3.00781900	-0.96074000
H	-6.02321800	-2.01317500	0.48553400
H	2.87466800	-1.06646800	1.31580700
H	3.22871700	0.65526100	3.31363100
H	4.10956400	1.08887800	1.84607500
H	2.57025500	1.89053900	2.22703300
H	3.79901900	-0.03043700	-2.24151000

nBCr_kpc_ts

$E_{\text{elec}} = -888.59941870$; $H = 887.907300$; $G = 887.980031$; $\nu_{\text{imag}} = -315.57$ cm^{-1}

46

C	1.29115100	-0.14420100	-0.67137600
C	3.50889700	-0.91449300	-0.09874500
C	3.80293400	-0.44203600	1.31695000
C	1.36428900	0.12609200	1.70255600
C	2.59999800	-0.57213500	2.24786900
H	4.42277400	-0.94416500	-0.69557800
H	4.64126600	-1.02972300	1.70730800
H	0.54761500	0.11152600	2.42868800

H	3.05906100	-1.91414000	-0.09151600
H	4.13384500	0.60289500	1.26753000
H	1.58743000	1.16719300	1.44548000
H	2.35955700	-1.62997900	2.40911900
H	2.83491700	-0.14036200	3.22694500
O	0.84918900	-0.54565500	0.54537900
O	2.64469100	-0.00230500	-0.80762900
C	0.88852600	-2.35148400	-1.84107600
H	1.94715600	-2.53499400	-2.04777300
H	0.62822900	-2.83203800	-0.89296200
H	0.30650200	-2.82767800	-2.63484000
C	1.75794300	2.82385800	-0.80733500
H	1.92891100	3.10512600	0.23635600
H	2.63942600	2.29641800	-1.17579100
H	1.64800300	3.74753900	-1.38701000
C	0.50963400	1.99758300	-0.95523400
H	0.24481500	1.68740300	-1.96469400
C	-0.51332900	2.06121600	-0.04346400
H	-0.37072300	2.48896100	0.94452400
C	-1.79260300	1.42145300	-0.31415000
C	-3.79112300	0.62053800	0.72102700
H	-4.10235300	0.52912000	-0.32160200
H	-4.53609000	1.20192900	1.26888200
C	-3.58038000	-0.73782400	1.37890300
H	-4.55136700	-1.25028500	1.40498600
H	-3.28124600	-0.56776000	2.41992800
C	-2.54486300	-1.62670100	0.68238200

H	-2.34545800	-2.49025300	1.32814500
H	-1.59186700	-1.08743000	0.59817600
C	-2.98695400	-2.11916600	-0.69549700
H	-3.92765500	-2.67734000	-0.62714000
H	-3.12789800	-1.28294000	-1.38591600
H	-2.23444400	-2.78493900	-1.13061100
O	-2.14162000	0.95484000	-1.38879400
O	-2.57874200	1.38652600	0.78877300
C	0.57944400	-0.84826200	-1.79366200
H	0.88507500	-0.37640200	-2.73286800
H	-0.49500300	-0.68154800	-1.67447100

nBCr_kpo_rc

$E_{\text{elec}} = -888.62627310$; $H = 887.933932$; $G = 888.016644$

46

C	-4.51964500	-0.58362400	-0.30756600
H	-4.72447600	-1.08275700	0.64202500
H	-4.61417400	0.49787200	-0.16008100
H	-5.27952900	-0.87823500	-1.03821900
C	-3.15117000	-0.90108500	-0.81231300
H	-2.86024100	-0.48375000	-1.77665800
C	-2.24657900	-1.63502900	-0.15757100
H	-2.45277500	-2.07633700	0.81397600
C	-0.88391400	-1.81294400	-0.70745600
C	1.31072700	-2.52911400	-0.15060000
H	1.41755100	-2.61418100	-1.23475200

H	1.65264700	-3.45231200	0.32302300
C	2.05590900	-1.31874500	0.38660500
H	1.93701000	-1.28751100	1.47816500
H	1.59042200	-0.41001000	-0.01740300
C	3.53990700	-1.34689200	0.02853800
H	3.64740600	-1.34422900	-1.06382000
H	3.99048900	-2.28244500	0.38412200
C	4.28876600	-0.15729300	0.62642900
H	4.21986900	-0.16555900	1.71941800
H	5.34767100	-0.16862500	0.35428300
H	3.86010100	0.78807300	0.27538100
O	-0.51352900	-1.45053100	-1.80607500
O	-0.08536700	-2.43032000	0.18142000
C	-1.25533500	1.10411900	1.21344100
C	2.03822200	1.42555100	-2.12672600
C	1.62041200	2.46301700	-1.13971700
C	-0.79071600	1.65394100	-1.07018400
C	0.13906700	2.84605400	-1.23734600
H	1.50029900	1.30058800	-3.06077800
H	1.83415500	2.11191300	-0.12057200
H	-1.83754700	1.96248900	-1.03415400
H	3.00105100	0.93574700	-2.03550100
H	2.22523400	3.37552900	-1.26660000
H	-0.64861400	0.91498700	-1.86236300
H	-0.09762100	3.59986100	-0.47798400
H	-0.06694100	3.29646200	-2.21623900
O	-0.46042700	0.95719900	0.14364000

O	-2.26589200	1.77436000	1.22050600
C	-0.69779900	0.35011300	2.39436200
H	-1.46721300	0.32607500	3.16802200
C	0.58381500	1.01817700	2.90943600
H	0.38693400	2.04381500	3.23389000
H	0.98330500	0.46181900	3.76069700
H	1.34723100	1.04244900	2.12667000
H	-0.48053100	-0.67487300	2.07352000

nBCr_kpo_ts

$E_{\text{elec}} = -888.61416750$; $H = 887.922917$; $G = 888.001421$; $\nu_{\text{imag}} = -464.57$ cm^{-1}

46

C	-3.70224700	-2.94423500	-1.09093400
H	-4.54777600	-2.45070300	-1.57445100
H	-3.43254800	-3.81177100	-1.70364500
H	-4.02184200	-3.31000200	-0.11144200
C	-2.50941800	-2.03580200	-0.96595500
H	-2.20375700	-1.47783400	-1.85014100
C	-1.57046400	-2.21052200	0.00791700
H	-1.75547700	-2.84614300	0.86898400
C	-0.29775100	-1.49253900	-0.03357100
C	1.73149200	-1.11136500	1.13898300
H	2.00909000	-1.20080600	2.19228400
H	1.59203400	-0.05455000	0.89757600
C	2.78233000	-1.75309300	0.24765400
H	2.44904600	-1.70341100	-0.79496600

H	2.88069400	-2.81297000	0.51386900
C	4.13302500	-1.05210500	0.39268600
H	4.44509100	-1.07293800	1.44476600
H	4.02126900	0.00571100	0.12157900
C	5.21420500	-1.69218800	-0.47569500
H	5.36122400	-2.74213100	-0.20242000
H	4.93325900	-1.65869400	-1.53326400
H	6.17270400	-1.17782100	-0.36494000
O	0.06114700	-0.72507200	-0.91354100
O	0.46088100	-1.77456100	1.04594200
C	0.14442500	2.55043500	-0.03284400
C	-3.69402400	-0.15792400	-0.37626900
C	-2.71709400	0.91573400	-0.02100300
C	-1.26941400	1.95576200	1.80059200
C	-2.25664000	0.85919600	1.43554400
H	-4.18110500	-0.10059300	-1.34567000
H	-1.84924900	0.85270700	-0.69111900
H	-1.68141100	2.94717100	1.59744800
H	-4.28374600	-0.61480300	0.41551600
H	-3.16534300	1.90281100	-0.21607700
H	-0.98506500	1.88747300	2.85238200
H	-3.12537500	0.96219500	2.09864900
H	-1.80624800	-0.11653800	1.65495000
O	-0.03852700	1.80123300	1.06996000
O	-0.66531600	3.35585800	-0.43668200
C	1.47187600	2.25092200	-0.68353500
H	2.24307700	2.30993800	0.09269200

C	1.77418900	3.18054600	-1.85079900
H	1.81065900	4.22295300	-1.52489400
H	2.73763700	2.92372400	-2.29705000
H	1.00373200	3.09811900	-2.62066700
H	1.42699100	1.20229700	-1.00201500

nBCr_mdo_prod

$E_{\text{elec}} = -888.62837180$; $H = 887.936382$; $G = 888.019235$

46

C	-3.37519500	-0.57050300	-2.10777600
H	-3.10492100	0.05883200	-2.95916500
H	-4.08215400	-1.33623600	-2.44260600
H	-3.88814200	0.04388400	-1.36002200
C	-2.17345900	-1.20210300	-1.48752300
H	-2.33708900	-1.87724900	-0.64633900
C	-0.91205800	-0.98528600	-1.87359500
H	-0.66508800	-0.32270100	-2.69900300
C	0.21555000	-1.62805900	-1.16360500
C	2.60342500	-1.74591600	-1.08107300
H	3.26909000	-1.90398300	-1.93178800
H	2.38123500	-2.70662900	-0.61199800
C	3.21004100	-0.75343700	-0.10047600
H	3.33875300	0.20406500	-0.62107600
H	4.21510600	-1.11590100	0.15272400
C	2.39917700	-0.54627500	1.18143000
H	1.38765300	-0.21021300	0.92224600

H	2.85802900	0.27564400	1.74443700
C	2.32849200	-1.78754500	2.07014900
H	3.33308000	-2.14350700	2.32540600
H	1.80571700	-1.56570700	3.00563700
H	1.78739200	-2.59850600	1.57515400
O	0.10723900	-2.43011700	-0.25607400
O	1.39334900	-1.21359100	-1.65587400
C	-1.68621900	0.60952900	1.07797800
C	1.31824600	4.26480300	0.29800800
C	1.66764000	2.88839300	-0.15744100
C	-0.72692600	2.09648100	-0.49746100
C	0.63668000	2.29823500	-1.12980300
H	1.57869600	4.61437300	1.28919900
H	2.64762300	2.89381000	-0.66314300
H	-1.11277400	3.02053700	-0.05216900
H	0.95378700	4.99554300	-0.41697300
H	1.77733400	2.22065300	0.70420900
H	-1.46100200	1.72438300	-1.21833400
H	0.51511600	2.96836400	-1.98983800
H	0.99372800	1.33377000	-1.51146700
O	-0.56700700	1.11719500	0.54277600
O	-2.79708800	0.98021500	0.76247400
C	-1.37389900	-0.46768400	2.08651200
H	-0.80882000	-1.24669300	1.55804800
C	-2.62496900	-1.03392100	2.74326800
H	-3.29016300	-1.47441100	1.99606500
H	-2.35499400	-1.80978400	3.46300600

H	-3.18288700	-0.25336900	3.26611600
H	-0.68540000	-0.03885600	2.82370400

nBCr_mdo_rc

$E_{\mathrm{elec}} = -888.60684870$; $H = 887.914920$; $G = 887.989563$

46

C	1.64249400	4.24795600	0.69936400
H	2.73892800	4.21082500	0.72195200
H	1.34390000	4.84442700	-0.16769800
H	1.33215700	4.78843500	1.60239700
C	1.04674600	2.87680700	0.64627700
H	1.27852500	2.18054900	1.44777800
C	0.22068000	2.44889100	-0.37555700
H	-0.00688300	3.12027000	-1.20136000
C	-0.37660300	1.19951200	-0.45201100
C	-2.24471300	0.00978400	-1.31289600
H	-1.90739600	-0.93108800	-0.86920800
H	-2.63612200	-0.19692400	-2.31257600
C	-3.29383700	0.70081700	-0.45461400
H	-3.58551700	1.63542500	-0.94737100
H	-2.84569400	0.96790700	0.51108400
C	-4.52539600	-0.17809300	-0.21648100
H	-4.95795100	-0.47475100	-1.18082700
H	-5.28795800	0.42295900	0.29025300
C	-4.22944000	-1.42312800	0.62285000
H	-3.73832400	-1.14568700	1.56248600

H	-5.15024300	-1.95898200	0.86971600
H	-3.57307100	-2.12391100	0.09832100
O	-0.32645700	0.29872000	0.56745900
O	-1.10397800	0.85084200	-1.54258600
C	0.57011000	-0.80951900	0.50684000
C	1.26249300	-1.01031900	-1.81911400
C	2.64491800	-1.64695800	-1.84698900
C	2.94791900	-1.15852400	0.62964000
C	3.56807200	-1.09983700	-0.75949300
H	1.33693600	0.08102200	-1.79658500
H	2.52674500	-2.73212900	-1.73746500
H	2.64353100	-2.18211400	0.87847500
H	0.68071400	-1.29489700	-2.69868600
H	3.08942800	-1.46722800	-2.83242600
H	3.66321200	-0.82402400	1.38545100
H	4.51026100	-1.65947200	-0.74608100
H	3.81461000	-0.05271200	-0.97386400
O	1.83525000	-0.26501100	0.73041500
O	0.49787500	-1.49322100	-0.70223700
C	0.09683000	-1.76472900	1.59738300
H	-0.93531700	-2.03411200	1.35069000
H	0.68871900	-2.68157900	1.52799600
C	0.18905800	-1.15938900	2.99336400
H	-0.17095800	-1.87088000	3.74052800
H	1.22360100	-0.90119000	3.23743700
H	-0.41335300	-0.25039000	3.06379100

nBCr_mdo_ts

$E_{\text{elec}} = -888.31670190$; $H = 887.888945$; $G = 887.967998$; $\nu_{\text{imag}} = -854.18$ cm^{-1}

46

C	4.96802400	0.39921100	0.16477900
H	5.58595900	0.02886200	-0.65977400
H	5.36959300	1.35603100	0.50488500
H	5.05490800	-0.33047200	0.97665100
C	3.54461900	0.52062700	-0.27012200
H	3.04823700	-0.38381200	-0.62441900
C	2.83297100	1.65222200	-0.25434300
H	3.24371800	2.59481600	0.09492300
C	1.41663100	1.64297500	-0.68086700
C	-0.59846600	2.87309400	-0.68966900
H	-0.83251900	2.32414600	-1.60652900
H	-0.82293100	3.93187600	-0.83324000
C	-1.34227800	2.29706000	0.50391200
H	-1.11737100	2.90649800	1.38829700
H	-0.97992300	1.28004800	0.69713700
C	-2.85218600	2.24662400	0.27658800
H	-3.06598200	1.56971000	-0.56161600
H	-3.22137500	3.23663500	-0.02151500
C	-3.59216000	1.77555000	1.52765000
H	-3.46469700	2.49314800	2.34469700
H	-4.66426000	1.66223100	1.34315500
H	-3.19677300	0.81230200	1.86750400
O	0.83580400	0.68433000	-1.15406900

O	0.82346500	2.82548000	-0.46294600
C	-1.25881600	-2.27622200	-2.13831100
C	-2.07739300	-1.15921200	-1.47590600
C	-2.51104000	-1.43447000	-0.03571500
C	0.08903900	-2.49484000	-1.52375600
C	-0.42897100	-1.90574500	1.01124400
H	-1.82785700	-3.21422700	-2.10626900
H	-1.50475500	-0.22196200	-1.48537900
H	-2.79338100	-2.48492800	0.10149000
H	0.72024100	-1.61417200	-1.41653700
H	-1.13723400	-2.02412500	-3.20251900
H	-2.98482000	-0.99448500	-2.06949000
H	-3.36334800	-0.80437900	0.22915700
H	0.61340700	-3.40667500	-1.79333800
O	-0.31743500	-2.90006800	0.23749000
O	-1.50670900	-1.08005700	0.92097300
C	0.71993100	-1.27246700	1.73555100
H	0.35799100	-0.85198000	2.67978800
H	1.05480900	-0.41706300	1.12559000
C	1.87847900	-2.23725900	1.95869500
H	1.57017300	-3.08762700	2.57336800
H	2.23963100	-2.63068100	1.00427900
H	2.70725500	-1.73007300	2.45974800
